# Practical sparse data-driven constitutive modeling via transfer learning in physics-encoded neural networks

**DOI:** 10.1038/s41598-025-34925-0

**Published:** 2026-01-05

**Authors:** Zhihui Wang, Roberto Cudmani

**Affiliations:** Department of Civil and Environmental Engineering, Technical University of Munich, Munich, 80333 Germany

**Keywords:** Physics-encoded neural networks, Constitutive model, Soil mechanics, Hypoplasticity, Machine learning, Transfer learning, Engineering, Materials science, Mathematics and computing

## Abstract

Data-driven constitutive models, owing to their inherent flexibility, can outperform traditional plasticity-based models in certain aspects. When calibrating these models, ensuring adherence to fundamental mechanical principles allows the calibrated models, referred to as physics-encoded neural networks (PeNNs), to be effectively integrated into finite element method (FEM) software for boundary value problem simulations. However, calibration challenges arise when only limited data are available. Addressing this issue, this study employs transfer learning. Synthetic labeled data, derived from traditional constitutive models were used to pre-train PeNNs. Subsequently, these pre-trained PeNNs are fine-tuned using implicitly labeled data from high-fidelity experimental records. The fine-tuned models are integrated into FEM software as user materials to conduct extensive drained and undrained triaxial test simulations. An analysis of the simulation results highlights the impact of the available volume of experimental data, the quantity of synthetic data, and key configurations in the fine-tuning process, such as the architecture of the fine-tuning model, frozen parameters, and batch size. Results indicate that through robust PeNN models and meticulous modeling, transfer learning can establish a data-driven constitutive model with limited experimental records, achieving superior simulation performance compared to the synthetic model alone. This underscores the potential of combining cost-effective synthetic and experimental data to advance constitutive modeling.

**Supplementary Information:**

The online version contains supplementary material available at 10.1038/s41598-025-34925-0.

## Introduction

In the field of Architecture, Engineering, and Construction, Computer-Aided Design facilitates precise geometric modeling, while Computer-Aided Engineering enables simulation-based analysis of mechanical performance^1,2^. Together, they support integrated design workflows, enhance decision-making and improve the efficiency and reliability of construction and engineering processes^3^. In this workflow, constitutive models provide critical support for simulating complex boundary value problems by effectively describing the stress-strain relationships observed in experiments^4^. They play a key role in various numerical algorithms such as finite element methods (FEM), finite difference methods, smoothed particle hydrodynamics, the material point method, and the particle finite element methods^5–7^. Due to the inherent complexity of geomaterials, it becomes necessary to introduce appropriate simplifications and assumptions^8^ to establish practical phenomenological constitutive models that enable reliable simulations. Over the years, these phenomenological constitutive models have undergone significant developments, evolving from simple linear elastic models, such as Hooke’s Law^9^, to models that reflect nonlinear elasticity, like the modified Duncan-Chang model^10^, and plastic constitutive models that account for irreversible deformations, such as the modified Mohr-Coulomb model^11^. These advancements have further extended to various models based on critical state soil mechanics^12–14^.

Natural geomaterials, with their complex mineral compositions and microstructures, present challenges in characterizing their mechanical behavior^15^. To more accurately simulate the stress-strain relationships observed under various conditions, traditional phenomenological plasticity models have increasingly incorporated additional material parameters and state variables^16^. The proliferation of these parameters inevitably raises issues related to parameter calibration and mutual interactions^17^. Particularly, certain state parameters are challenging to measure directly in experiments and initialize accurately, yet proper initialization is crucial. In light of these challenges, researchers are considering the use of artificial intelligence (AI) to describe constitutive relationships^18–22^, paralleling the efforts in other inverse problems^23^.

In developing constitutive models assisted by artificial intelligence, several factors must be considered: the model’s ability to reproduce experimental phenomena^24^, simplicity and efficiency of implementation^25^. Predominant AI-based constitutive models^26–31^ focus on replicating experimental behavior from a single-step prediction perspective. This approach involves using entire strain loading paths and sub-step information, with strain increments or strain rates as inputs to the machine learning model. However, this method does not ensure consistency with physical or mechanical principles and decouples model development from its application (initial boundary value problem^32^. Nevertheless, it serves as a test for the applicability of machine learning algorithms in constitutive model development^33^.

On the other hand, AI methods, while relying on fewer assumptions and capable of simulating experimental phenomena more accurately through careful design and analysis, require a larger number of parameters and face challenges in exorability and interpretability. Consequently, AI methods that integrate physical information are increasingly valued^31,34–36^. These applications include both soft constraints, which are parameter-dependent and consider physical information during training without guaranteeing its fulfillment by the framework itself, and hard constraints, which enforce certain physical and mathematical assumptions through the model framework itself. Hard constraints may include conditions such as matrix positive definiteness and reversibility^37,38^, input convexity^39,40^, temporal continuity^36^, objectivity^41,42^, thermal consistency^43,44^, and rate independence. Hard constraints are generally preferred despite their potential limitations on flexibility and representational capability.

If the developed data-driven constitutive model ensures strict adherence to fundamental mechanical assumptions, such as objectivity, constant stress under zero deformation rate, and insensitivity to the number of sub-steps in the loading process (rate-independence), it holds the potential for stable simulation of boundary value problems (BVPs). Following this, it is imperative to maintain adequate accuracy to successfully implement such a model in FEM software for BVPs. With access to reliable and sufficiently diverse experimental data, one can in principle construct a constitutive model directly from test observations. However, this approach is often impractical, as typical engineering projects only conduct a limited number of laboratorial triaxial tests, which generally suffice only to calibrate the model parameters.

To facilitate the use of limited, expensive and noisy indoor experiments^45,46^ in the development of data-driven constitutive models, this study employs a transfer learning approach. This method involves retaining some parameters of a pre-trained model while retraining the remaining parameters^47^, ensuring that subsequent updates do not cause catastrophic forgetting of previously acquired knowledge. This significantly accelerates the learning process in environments with limited data availability^48–51^. Multi-fidelity data supports the development of data-driven constitutive models, which are based on physics-encoded neural networks (PeNNs). These models enforce a hard constraint on fundamental physical and mechanical properties. The specific methods and model architectures are discussed in Sect. 2, while Sect. 3 outlines the PeNN calibration process using both low-fidelity and high-fidelity data. The calibrated models are then integrated as User Material (UMAT) into Abaqus for extensive triaxial test simulations. The successive implementation of PeNN-based UMAT in BVP simulation with mixed boundary conditions guarantees its further application in broader geotechnical problems, such as slope stability, foundation settlement, and tunnel excavation. Sections 4 and 5 analyze how the volume of available data, fine-tuning configurations, and parameter optimization setting impact the results. The findings demonstrate that this framework can achieve satisfactory outcomes with limited experimental data, providing guidance for future development of limited data-driven constitutive models.

In this study, $\:T$ represents effective Cauchy stress (true stress) tensor; $\:\dot{T}$ is the objective Jaumann stress rate; $\:D$ denotes the stretching tensor (strain rate tensor). The secondary unit tensor is denoted as $\:I$, while the fourth order unit tensor $\:J$ equals $\:0.5{I}_{ik}{I}_{jl}+0.5{I}_{il}{I}_{jk}$. $\:e$ represents the current void ratio. The following tensorial functions are used: $\:trX$ calculates the trace of tensor $\:X$; $\left\| X \right\|$ indicates the Euclidean norm of $\:X$; $\:\widehat{X}$ scales $\:X$ into $\:X/trT$; and $\:{X}^{*}$ stands for the deviatoric part of tensor $\:X$ as $\:{X}^{*}=X-I\:trX/3$.

## Methodology

In the development of data-driven constitutive models, data plays a pivotal role. Typically, a distinction is made between high-fidelity and low-fidelity data. High-fidelity data, obtained from controlled experiments, are considered reliable due to their direct correlation with real-world conditions, despite containing inherent measurement errors and ubiquitous noise. This type of data faithfully captures the phenomena under study and thus forms the basis for a high-quality model foundation.

Conversely, low-fidelity data originates from simplified, albeit carefully constructed and calibrated, constitutive models. These provide approximate solutions that are cost-effective but less precise. Such simulations tend to overlook complexities present in real-world conditions, potentially leading to significant deviations from experimental data, particularly as phenomenological constitutive models may not fully capture the nuances of soil tests.

In this study, both data types were employed in a complementary fashion: low-fidelity data helped outline general trends, while high-fidelity data supported further calibration of these trends. The initial model, driven by synthetic data, is referred to as the pre-trained model, which is subsequently refined using experimental data.

**Fig. 1:**
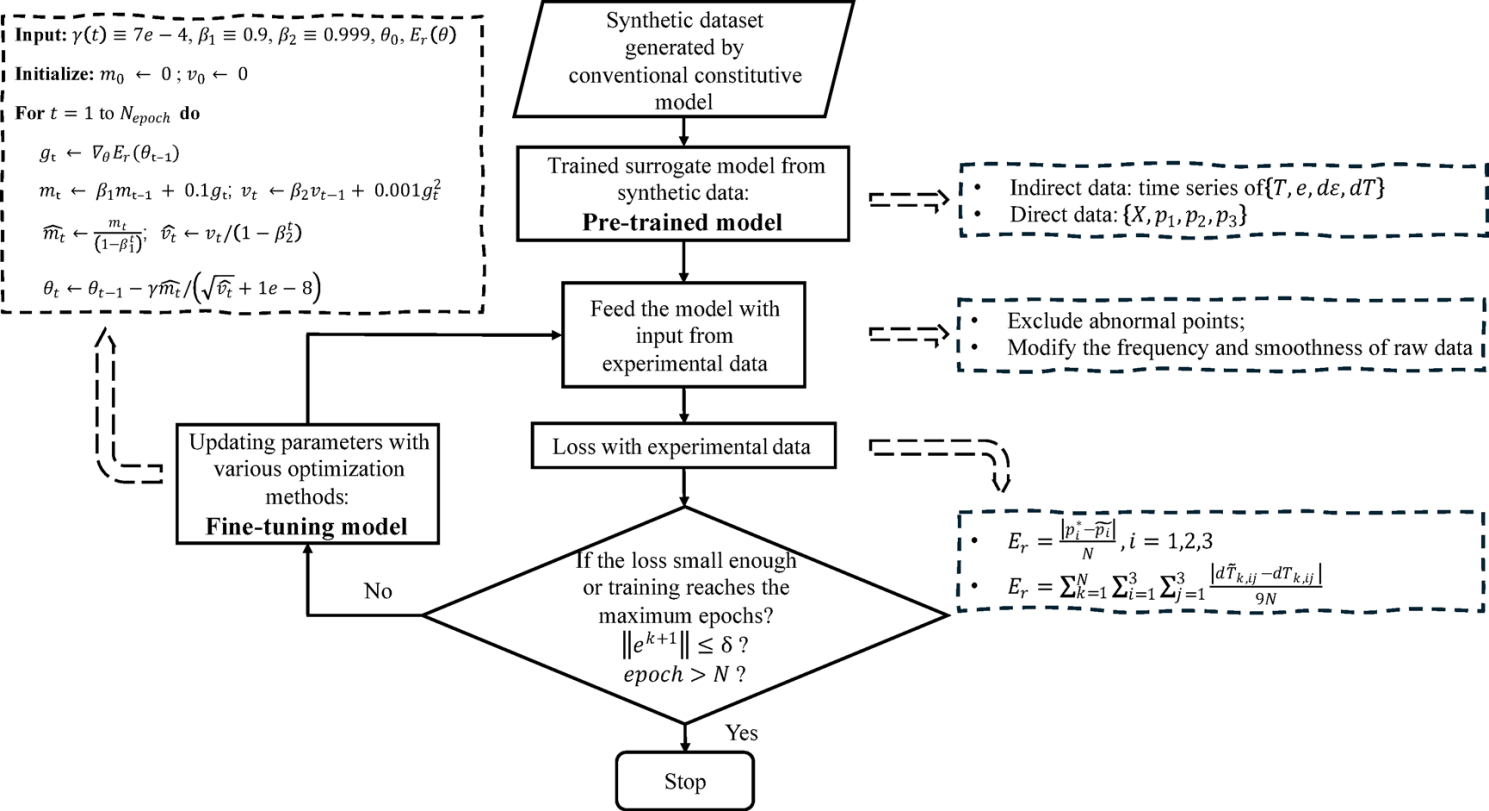
Diagram of utilizing multi-fidelity data in this study.

**Fig. 2:**
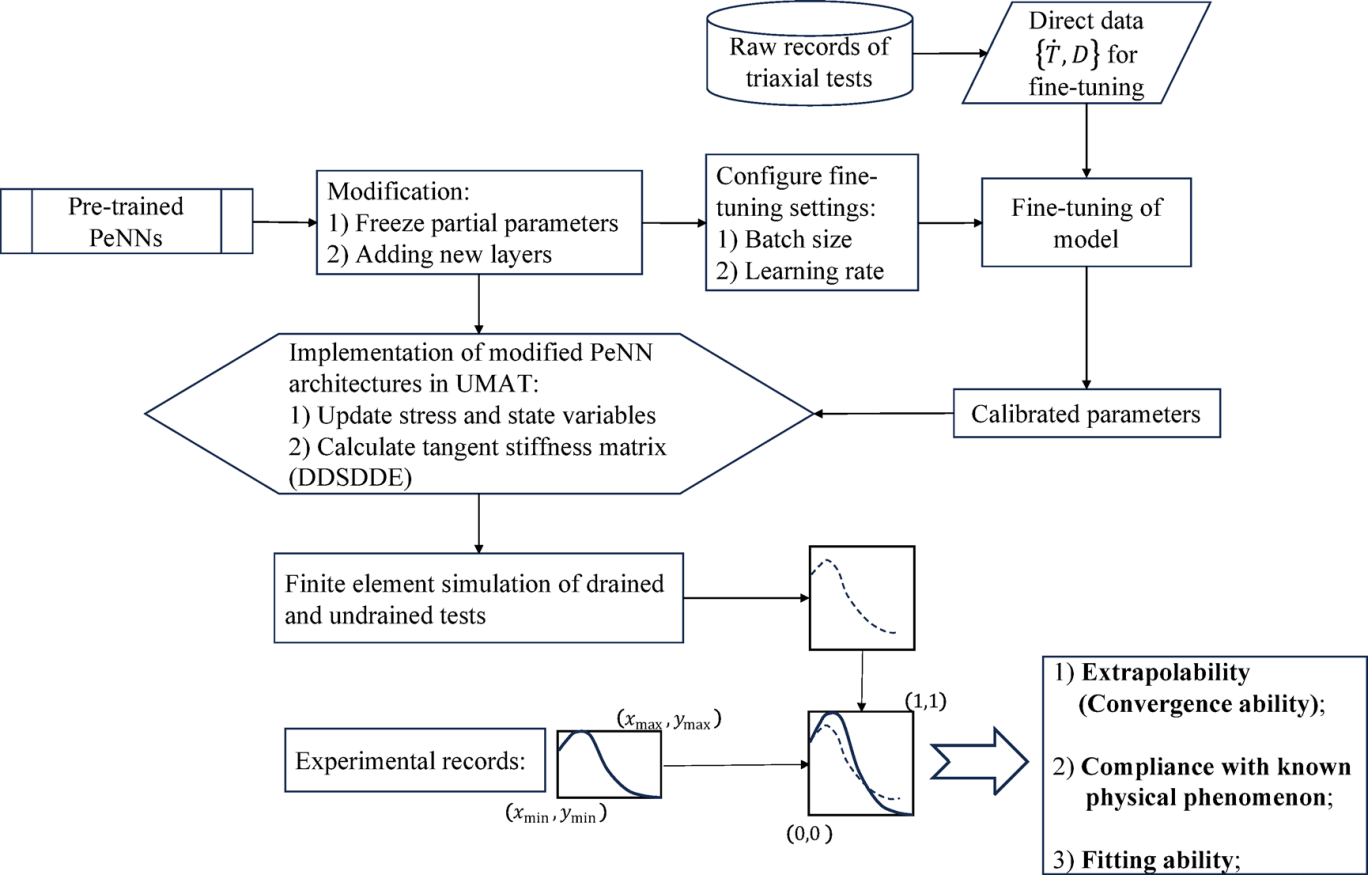
Fine-tuning and implement the fine-tuned models in BVPs.

Figure 1 succinctly illustrates the process. It begins with numerical simulations to generate synthetic data spanning a broad spectrum of stress and state-variable conditions. This synthetic data is then used to pre-train the model, followed by the use of experimental data to fine-tune it. The left portion of the figure presents a detailed description of parameter updating, including specific parameter values, to ensure reproducibility. The specific progress of this fine-tuning and the implementation of the refined models in FEM simulations are detailed in Fig. 2.

###  Reference model

A well-defined pre-trained model provides an excellent starting point for data-driven constitutive models, contingent upon selecting an appropriate constitutive model to generate low-fidelity data^52^. proposed a hypoplastic model framework that has gained widespread adoption and continuous updates. Consequently, the present study employs this hypoplastic model as a reference to pre-train the data-driven model. In this context, the reference model calculates the stress rate by the following expression:

1 $$\:\begin{array}{c}\dot{T}={f}_{s}\frac{1}{\widehat{T}:\widehat{T}}\left({F}^{2}D+{a}^{2}\widehat{T}\otimes\:\widehat{T}:D\right)+{f}_{s}{f}_{d}\frac{Fa}{\widehat{T}:\widehat{T}}\left(\widehat{T}+{\widehat{T}}^{*}\right)\left\| D \right\|\end{array}$$

where $\:F$ reflects current stress state and $\:a$ is a parameter constant; The barotopy factor $\:\left({f}_{s}\right)$ and pyknotropy factor ($\:{f}_{d}$) consider the influence of pressure level and relative density, respectively. The parameter $\:a$ controls the peak friction ratio and is defined as $\:a=\frac{\sqrt{3}\left(3-{{sin}\phi\:}_{c}\right)}{2\sqrt{2}{{sin}\phi\:}_{c}}$, where $\:{\phi\:}_{c}$ is friction angle. The expression of $\:F$ corresponds to the Matsuoka-Nakai failure criterion^53^. Its expression is

2 $$F = \sqrt {\frac{3}{8}tr\left( {\hat{T}^{{*^{2} }} } \right) + \frac{{2 - 3tr\left( {\hat{T}^{{*^{2} }} } \right)}}{{2 - 6tr\left( {\hat{T}^{{*^{3} }} } \right)/tr\left( {\hat{T}^{{*^{2} }} } \right)}}} - \frac{{\sqrt 6 }}{4}\left\| {\hat{T}^{*} } \right\|$$.

The factors $\:{f}_{s}$ and $\:{f}_{d}$ are defined as

3 $$\:\begin{array}{c}{f}_{s}=\frac{{h}_{s}}{n}{\left(\frac{{e}_{i}}{e}\right)}^{\beta\:}\frac{1+{e}_{i}}{e}{\left(\frac{-{tr}T}{{h}_{s}}\right)}^{1-n}{\left[3+{a}^{2}-a\sqrt{3}{\left(\frac{{e}_{i0}-{e}_{d0}}{{e}_{c0}-{e}_{d0}}\right)}^{\alpha\:}\right]}^{-1}\end{array}$$

and

4 $$\:\begin{array}{c}{f}_{d}={\left(\frac{e-{e}_{d}}{{e}_{c}-{e}_{d}}\right)}^{\alpha\:}\end{array}$$

respectively. The parameters $\:{e}_{i}$, $\:{e}_{c}$ and $\:{e}_{d}$ evaluate the current relative position of porosity, and can be calculated based on the pressure level as

5 $$\:\begin{array}{c}\frac{{e}_{i}}{{e}_{i0}}=\frac{{e}_{c}}{{e}_{c0}}=\frac{{e}_{d}}{{e}_{d0}}={exp}\left[-{\left(\frac{-trT}{{h}_{s}}\right)}^{n}\right]\end{array}$$

where $\:\alpha\:,\beta\:,{h}_{s},n,{e}_{i0},{e}_{c0},{e}_{d0}$ are material constants. These parameters were determined by careful calibration^54^.

### Data driven model

In the data-driven model, the stress rate is calculated as:

6 $$\:\begin{array}{c}\dot{T}\left(T,D,\overrightarrow{s}\right)={p}_{1}\left(T,\overrightarrow{s}\right)J:D+{p}_{2}\left(T,\overrightarrow{s}\right)\widehat{T}\otimes\:\widehat{T}:D+{p}_{3}\left(T,\overrightarrow{s}\right)\left(\widehat{T}+{\widehat{T}}^{*}\right)\parallel\:D\parallel\:\end{array}$$

where $\:\overrightarrow{s}$ is the vector containing all state variables. The functions $\:{p}_{i}$, for $\:i=\text{1,2},3$, are all implemented using a Multilayer Perceptron (MLP), described by:

7 $$\:\begin{array}{c}{p}_{i}={f}_{o}\left({W}^{\left[o\right]}{f}_{3}\left({W}^{\left[3\right]}{f}_{2}\left({W}^{\left[2\right]}{f}_{1}\left({W}^{\left[1\right]}x+{b}^{\left[1\right]}\right)+{b}^{\left[2\right]}\right)+{b}^{\left[3\right]}\right)+{b}^{[o]}\right)\end{array}$$

where $\:x$ is the input vector, $\:{W}^{\left[k\right]},k=\text{1,2},3$ is the weight matrix for the $\:k$-th hidden layer; $\:{b}^{k},k=\text{1,2},3$ is the bias vector for the k-th hidden layer; and $\:{f}_{k},k=\text{1,2},3$ is the activation function of of $\:k-$th hidden layer. The output layer weights and biases are $\:{W}^{\left[o\right]}$ and $\:{b}^{\left[o\right]}$, respectively, with $\:{f}_{o}$ as the activation function for the output layer. This framework has been validated as a data-driven surrogate constitutive model^41^. In this study, $\:{f}_{i},i=\text{1,2},3$ are set to bent-identity function, $\:{f}_{o}$ is chosen as linear function. The input vector is defined as:

8 $$\:\begin{array}{c}x=\left\{{T}_{iso},\overrightarrow{s}\right\}=\left\{trT,\left\| {\hat{T}^{*} } \right\|,\frac{{tr}\left({\widehat{T}}^{*}\cdot\:{\widehat{T}}^{*}\cdot\:{\widehat{T}}^{*}\right)}{{\left[{\widehat{T}}^{*}:{\widehat{T}}^{*}\right]}^{\frac{3}{2}}},e\right\}\end{array}$$

Thus, $\:{T}_{iso}\left(T\right)$ includes $\:\left\{trT,\left\| {\hat{T}^{*} } \right\|,\frac{{tr}\left({\widehat{T}}^{*}\cdot\:{\widehat{T}}^{*}\cdot\:{\widehat{T}}^{*}\right)}{{\left[{\widehat{T}}^{*}:{\widehat{T}}^{*}\right]}^{\frac{3}{2}}}\right\}$, which is actually the linear transformations of pressure level, deviatoric stress and Lode angle. The state vector is defined as $\:\overrightarrow{s}=e$. This architecture inherently ensures that, regardless of the MLP model’s parameters, the following fundamental mechanical assumptions are rigorously satisfied:

(1) Objectivity

9 $$\:\begin{array}{c}\dot{T}\left(RT{R}^{T},RD{R}^{T},\overrightarrow{s}\right)=R\dot{T}\left(T,D,\overrightarrow{s}\right){R}^{T}\end{array}$$

where $\:R$ is rotation matrix. This is because $\:{T}_{iso}\left(T\right)={T}_{iso}\left(RT{R}^{T}\right)$.

(2) Constant stress under constant configuration

10 $$\:\begin{array}{c}\dot{T}\left(T,0,\overrightarrow{s}\right)=0\end{array}$$.

Since all terms in $\:\dot{T}$ include $\:D$ as a factor, this condition is naturally met.

(3) Rate independence, namely

11 $$\:\begin{array}{c}\dot{T}\left(T,{k}^{2}D,\overrightarrow{s}\right)={k}^{2}\dot{T}\left(T,D,\overrightarrow{s}\right)\end{array}$$

This is because all terms in $\:\dot{T}$ that include $\:D$ have $\:D$ raised to the first power.

The corresponding tangent stiffness matrix is expressed as:

12 $$\:\begin{array}{c}\frac{\partial\:\dot{T}}{\partial\:D}={p}_{1}\left({T}_{iso},\overrightarrow{s}\right)J+{p}_{2}\left({T}_{iso},\overrightarrow{s}\right)\left(\widehat{T}\otimes\:\widehat{T}\right)+{p}_{3}\left({T}_{iso},\overrightarrow{s}\right)\left(\widehat{T}+{\widehat{T}}^{*}\right)\otimes\:\frac{D}{\parallel\:D\parallel\:}\end{array}$$

Encoding some physical information into the framework and model structure may reduce the model’s flexibility but, like traditional constitutive models, enforces stringent constraints on fundamental mechanical assumptions. Consequently, it effectively lowers the requirements for the number of parameters and the volume of training data. Therefore, in this study, each MLP model uses only three hidden layers, with 24, 12, and 6 nodes, respectively.

## Data and calibration of models

### Low-fidelity data and pre-training

In this study, Karlsruhe fine sand was selected as the research material due to the comprehensive and publicly available experimental records^54,55^. The stress rate in the PeNN model is calculated by determining the $\:{p}_{i}$ parameters based on the current stress and state variables, which are then multiplied by corresponding tensor terms. Thus, to achieve a well-performing PeNN, it is essential to sample $\:{p}_{i}$ across a sufficient range of stress states and relevant state variables.

**Fig. 3:**
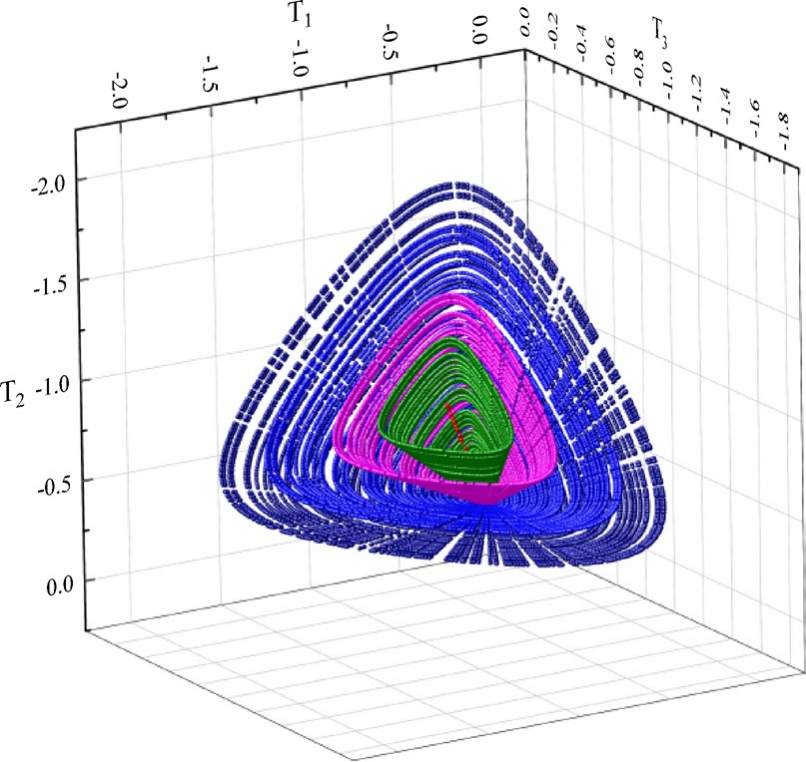
Distribution of sampled points in principal stress space.

Given that the reference model employs the Matsuoka-Nakai yield criterion, the sampling within the $\:T-e$ space was designed as illustrated in Fig. 3. The stress states are sampled along rays representing hydrostatic states to surfaces corresponding to the Matsuoka-Nakai failure criterion. For each stress state shown in the figure, sampling is also performed across the void ratio space to calculate corresponding values of $\:{e}_{i}$ and $\:{e}_{d}$, and points are selected within the interval from $\:{e}_{d}$ to $\:{e}_{i}$ at increments of 0.1. Using the reference model, the actual values of $\:{p}_{1}$, $\:{p}_{2}$, and $\:{p}_{3}$ are computed for these sampling points.

Once the training dataset is constructed, $\:{T}_{iso}\left(T\right)$ is calculated along with the void ratio to serve as inputs. Subsequently, the three MLP networks are trained independently using the Mean Absolute Error (MAE) as the loss function:

13 $$\:\begin{array}{c}{E}_{r}=\frac{\left|{p}_{i}^{*}-\stackrel{\sim}{{p}_{i}}\right|}{N},i=\text{1,2},3 \end{array}$$

where N is the number of sampling points, superscript *denotes the true values, and overhead tilde represents the MLP predicted values. The weight matrices and biases of MLPs are initialized using the Glorot initialization, and these parameters are optimized using the Adam optimizer over a total of 1,000 training epochs. The specific configuration is listed in Table 1.

**Table 1:** Configuration for pre-training the PeNNs.

Configuration	Content	Reason for selection
Activation function	Bent-identity	Infinite order of continuity, infinitely differentiable, meaning its derivatives of all orders exist for all real numbers.
Loss function	Hub loss function	Stronger resistance to noise; and is not so sensitive to outliers
Optimization algorithm	Adam	Effective and widely used
Training epochs	1000	Saving time, loss reduce slowly at then
Learning rate	6e-4	Careful search and used in cooperation with batch size and number of epochs
Batch size	32	Balance the speed and extrapolation ability

**Fig. 4:**
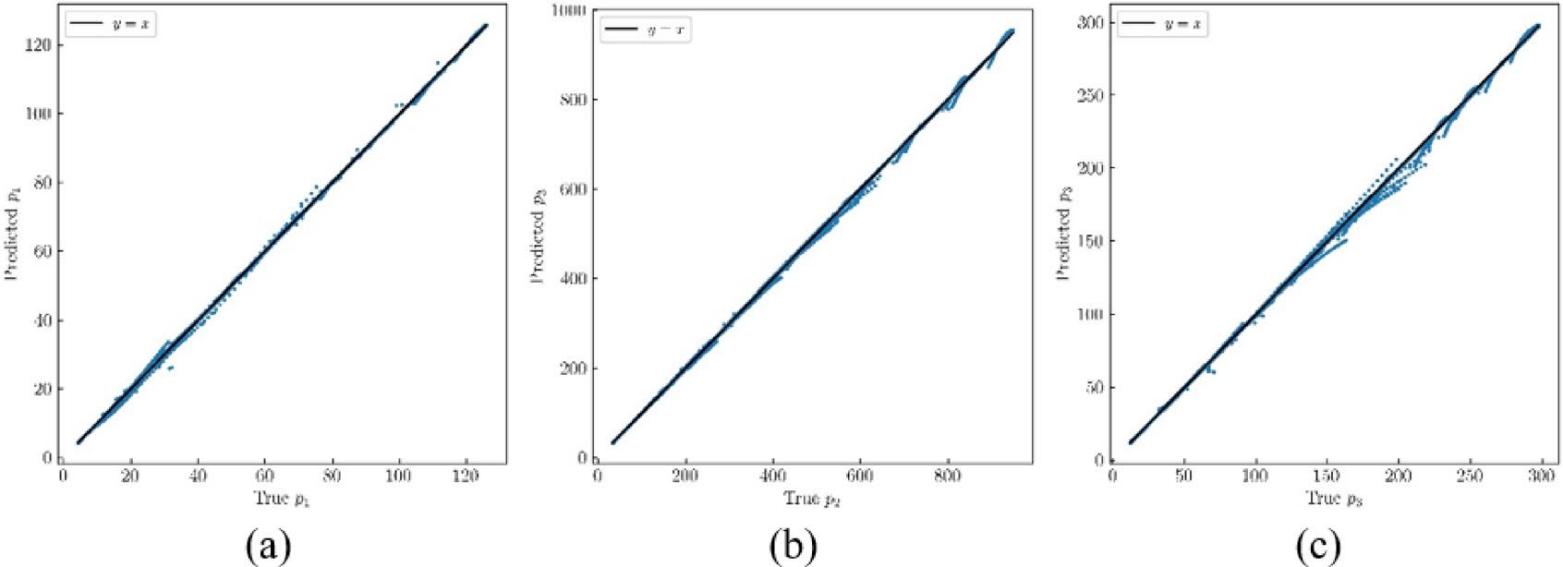
Performance of pre-trained network for (**a**) $\:{p}_{1}$; (**b**) $\:{p}_{2}$; (**c**) $\:{p}_{3}$;

After training, all three models achieved favorable outcomes. For the MLP corresponding to $\:{p}_{1}$, the MAE is 0.0317 and R² Score is 0.9999. The MLP for $\:{p}_{2}$ recorded an MAE of 0.4053 and an R² Score of 0.9999. For the MLP of $\:{p}_{3}$, the MAE is 0.2157, and the R² Score is 0.9999. A comparative visualization of the actual versus predicted $\:{p}_{i}$ values is presented in Fig. 4.

**Fig. 5:**
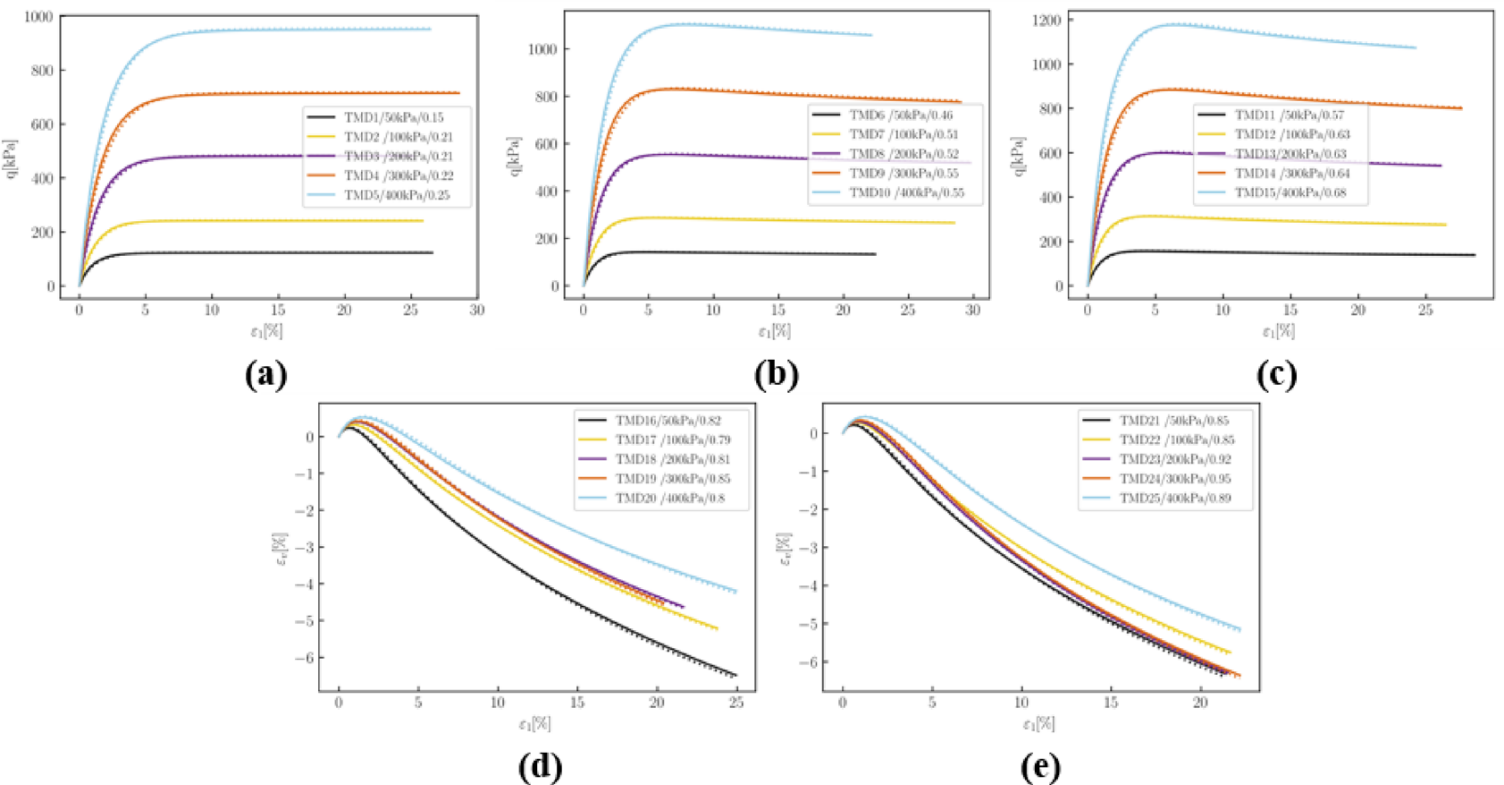
Deviatoric stress$\:\:q$ versus axial strain $\:{\epsilon\:}_{1}$in drained simulations: (**a**) loose samples; (**b**) loose to medium dense samples; (**c**) medium dense samples; (**d**) medium dense to dense samples; (**e**) dense samples.

Upon obtaining the trained parameters, it is necessary to replicate the network structure and the process for calculating the stress rate in Fortran. This involves scripting the UMAT representing data-driven constitutive models, then debugging the UMAT, and finally integrating it into Abaqus for simulating BVPs. Simulations of drained experiments using both the reference model UMAT and the PeNN model UMAT were conducted, yielding the results shown in Fig. 5:

It is evident from Fig. 5 that the pre-trained model’s results (dotted lines) closely align with those of the reference model (solid lines).

### High-fidelity data and fine-tuning

During the fine-tuning phase, experimental data are required to calibrate the data-driven constitutive model. This task is challenging because the experimental records do not explicitly contain the values of $\:{p}_{i},i=\text{1,2},3$; they only implicitly reflect the influence of $\:{p}_{i}$. In other words, the dataset lacks explicit labels for $\:{p}_{i}$, which prevents direct supervised learning during fine-tuning. Thus, the high-fidelity data used here does not mean that actual values of $\:{p}_{i}$ were recorded during the experiments. Therefore, referring to this phase as ‘calibration’ rather than ‘training’ is more appropriate.

Moreover, the experimental records lack a direct, noise-free recording of stress and strain tensors. This means that the values of $\:\dot{T},T,D$ and $\:e$ used for fine-tuning the model are approximated estimates, further complicating the task. Importantly, since data points are recorded discretely, $\:\dot{T}$ cannot be derived directly from time series. Instead, only the cumulative stress increment $\:dT=\int\:\dot{T}dt$ is available, as shown in:

14 $$\:\begin{array}{c}d{T}_{n+1}={\int\:}_{{t}_{n}}^{{t}_{n+1}}\dot{T}dt\approx\:{p}_{1}\left({T}_{iso,n},{\overrightarrow{s}}_{n}\right)J:d{\epsilon\:}_{n+1}+{p}_{2}\left({T}_{iso,n},{\overrightarrow{s}}_{n}\right){\widehat{T}}_{n}\otimes\:{\widehat{T}}_{n}:d{\epsilon\:}_{n+1}\\\:+{p}_{3}\left({T}_{iso,n},{\overrightarrow{s}}_{n}\right)\left({\widehat{T}}_{n}+{{\widehat{T}}_{n}}^{*}\right)\parallel\:d{\epsilon\:}_{n+1}\parallel\:\end{array}$$

Therefore, the model estimates the average secant stiffness, instead of instantaneous tangent stiffness, in the fine-tuning process. Previous research^41^ indicates that calibrated models are still feasible under these conditions. The loss function is designed as follows:

15 $$\:\begin{array}{c}{E}_{r}={\sum\:}_{k=1}^{N}{\sum\:}_{i=1}^{3}{\sum\:}_{j=1}^{3}\frac{\left|d{\stackrel{\sim}{T}}_{k,ij}-{dT}_{k,ij}\right|}{9N}\: \end{array}$$

where $\:\left\{{dT}_{1},{dT}_{2},\cdots\:,{dT}_{k},\cdots\:,{dT}_{N}\right\}$ represents the true stress increment derived from the enhanced experimental data. $\:d\stackrel{\sim}{T}{\left({X}_{k},{\uptheta\:}\right)}_{ij}$ denotes the estimated stress increment for $\:{X}_{k}$, and $\:N$ is the total number of data points in the dataset. Given that this study assumes limited experimental data; to maximize data utilization, some models consider an additional loss component based on $\:dT$ as described below:

16 $$E_{a} = \mathop \sum \limits_{{k = 1}}^{N} \left( {\frac{{\left| {tr\left( {d\tilde{T}_{k} } \right) - tr\left( {dT_{k} } \right)} \right|}}{N} + \frac{{\left| {d\tilde{q}_{k} - dq_{k} } \right|}}{N} + \frac{{\left\| {t_{k} } \right\| \cdot \left\| {\tilde{{t}_{k}} } \right\| - \left| {t_{k} \cdot \tilde{{t}_{k}} ^{T} } \right|}}{{1000 \cdot N \cdot \left\| {t_{k} } \right\| \cdot \left\| {\tilde{{t}_{k}} } \right\|}}} \right)$$

where the overhead tilde means predicted values, $\:q$ is the deviatoric stress, $\:t$ is the main diagonal $\:dT$, $$\:t=\left(\begin{array}{ccc}{dT}_{11}&\:d{T}_{22}&\:d{T}_{33}\end{array}\right)$$ and $\:\stackrel{\sim}{t}$ represents the main diagonal of $\:d\stackrel{\sim}{T}$. The last part evaluates the difference between the directions of $\:dT$ and $\:d\stackrel{\sim}{T}$, where ‘1000’ is used to limit its size. During the fine-tuning process, the model structure remains unchanged, and specific configurations used will be detailed when presenting the results.

### Integrate PeNNs into FEM software

During pre-training and fine-tuning, PeNNs are calibrated using material-point–level data with prescribed strain histories, obtained either from experiments or synthetic tests. In this setting, the constitutive response is evaluated without step-by-step integration as in the solution of a global boundary value problem. Even if the model is not perfectly accurate, its error only affects the update of state variables at the current loading step.

**Fig. 6:**
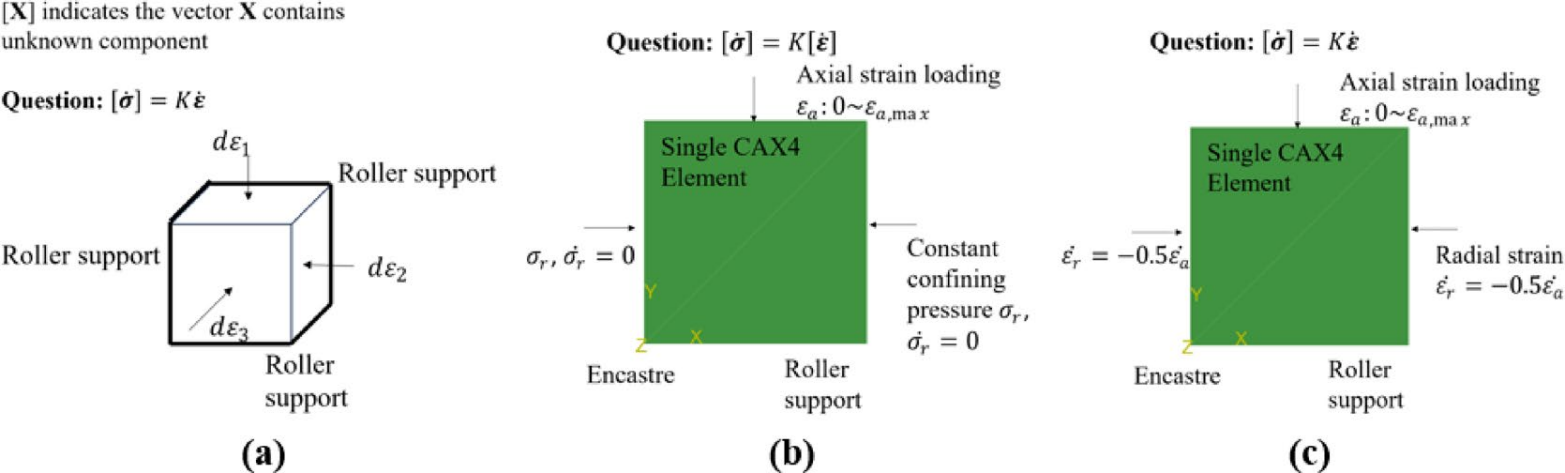
Loading conditions when training with Python and validation with Abaqus: (**a**) training with strain-control; (**b**) drained simulation with mixing control; (**c**) undrained simulation with strain-control.

Once embedded into a finite element code, however, the local strain evolution is no longer prescribed but instead emerges from the global equilibrium solution under the applied boundary conditions and the constitutive stiffness provided by the AI - constitutive model. Moreover, errors in the updates of strain, stress, and other state variables accumulate progressively during the integration process. As a result, the strain and stress paths experienced in finite element simulations may deviate from those used during training. For example, when implemented in Abaqus, the strain history may be directly given or be obtained through a nonlinear iterative equilibrium procedure, as schematically illustrated in Fig. 6.

Therefore, a low calibration error at the material-point level does not necessarily guarantee robust convergence or consistent results in finite element boundary value problems. Objective assessment of data-driven constitutive models should thus be performed within finite element simulations. This part of the task, involving fine-tuning and finite element simulations, requires substantial computational resources, and parallel computing is recommended to accelerate the process.

## Results and discussion

For each fine-tuned model, finite element simulation software was utilized to simulate 25 different drained triaxial experiments, 6 undrained triaxial extension tests, and 6 undrained triaxial compression tests, all based on experimental conditions documented in the database^56^. In all the graphical representations that follow, solid lines denote experimental records, dotted lines represent simulations using the reference model’s UMAT, and dashed lines correspond to simulations with the fine-tuned model’s UMAT. Identical colors indicate the same simulation; labels such as TMD1/50 kPa/0.2 refer to the experiment’s name (e.g., TMD1 indicates triaxial monotonic drained test numbering 1, TMU1 indicates undrained triaxial monotonic test numbering 1), initial confining pressure (50 kPa), and initial relative density (0.2), using the naming conventions from the dataset^56^.

As mentioned in Sect. 2, convergence ability and compliance with physical phenomena are crucial when implementing a data-driven constitutive model in FEM simulations. An evaluation procedure tailored for granular constitutive models is then introduced and described below. Table 2 illustrates this procedure in detail.

The extrapolation ability can be checked through the performance of data-driven UMAT in FEM simulations. To quantify this, the convergence ratio can be defined as the proportion of successful FEM simulations that reach the target loading relative to the total number of simulations conducted. For example, the convergence ratio is 0.5 if only half of the simulations successfully converge to the designed target loading.

**Fig. 7:**
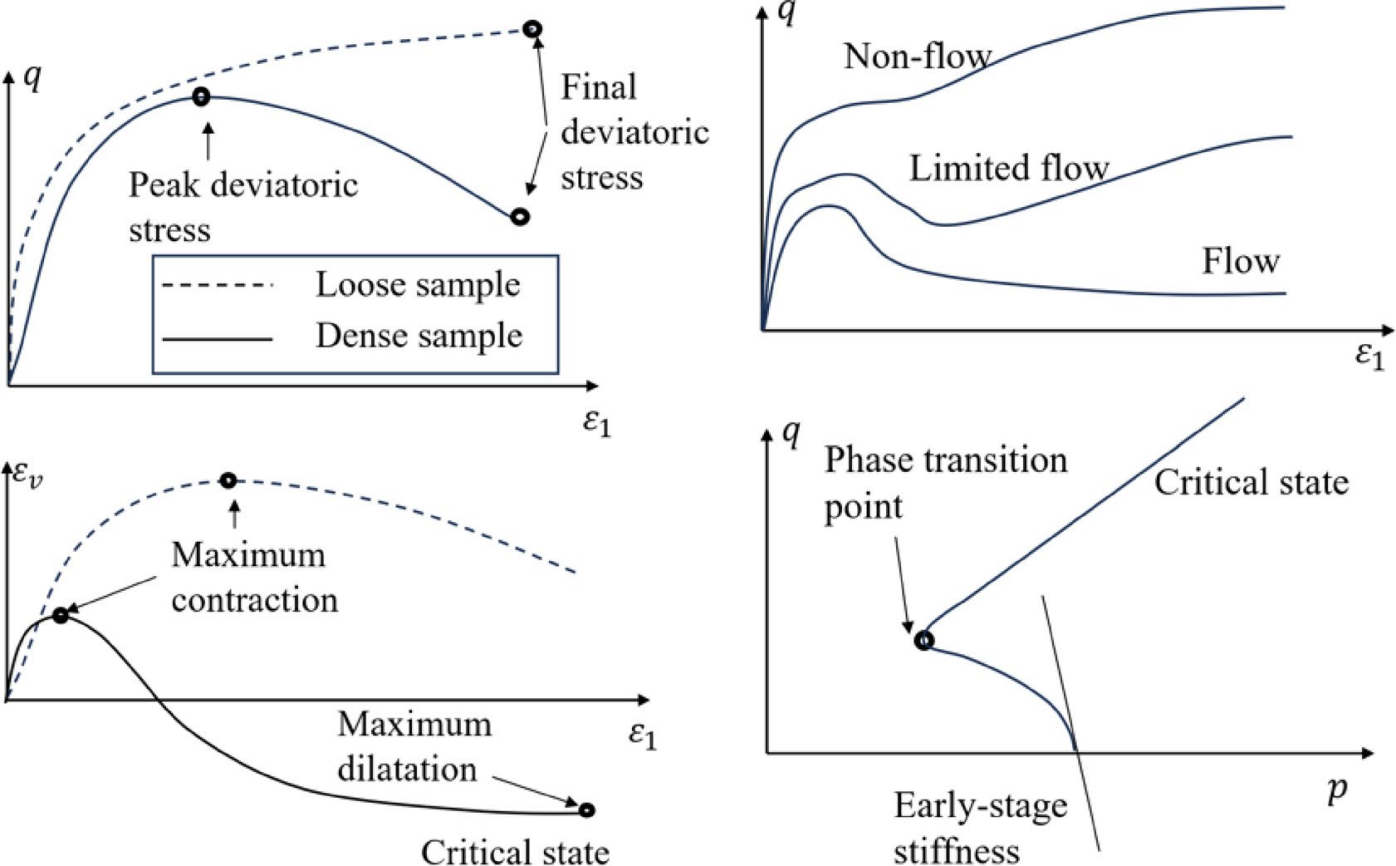
Important experimental phenomena to be checked, left drained test; right: undrained test.

As for the adherence to crucial physical phenomena, this can be evaluated by whether the constitutive model can reproduce crucial phenomenon observed in experiments, as shown in Fig. 7. These phenomena include but are not limited to (1) the ability to reflect critical state in drained and undrained tests under large deformation; (2) limited flow and flow in undrained tests; (3) phase transition in undrained test.

As for the fitting ability, we need to quantify the difference between simulation records and experimental records. The sampling frequency in numerical simulations often differs from that in experimental measurements, leading to inconsistencies in data resolution. This mismatch may result in numerical simulations having more or fewer data points than their corresponding experimental datasets. Consequently, traditional regression loss metrics, such as mean absolute error (MAE) or the $\:{R}^{2}$ coefficient, cannot be directly calculated. To address this, the dynamic time warping (DTW) algorithm is employed to robustly compare temporal sequences that may be misaligned. DTW is widely used to quantify mismatches between numerical and experimental curves. Prior to comparison, all curves are normalized based on experimental data, as illustrated in Fig. 2.

Then the effectiveness of fine-tuning, which aims to enhance performance beyond that of the pre-trained model, can be evaluated using the average improvement ratio defined as:

17 $$\:\begin{array}{c}\stackrel{-}{{W}_{rel}}=\sum\:_{i=1}^{{N}_{c}}\frac{{W}_{ref,i}}{{N}_{c}\cdot\:{W}_{nn,i}} \end{array}$$

where $\:{N}_{c}$ is the number of curves for comparison (the number of triaxial simulations); $\:{W}_{ref,i}$ indicates the normalized DTW distance between the experimental results and those from the reference model for the $\:i-$th curve, and $\:{W}_{nn,i}$ is the normalized DTW distance between the experimental results and those from the data-driven model for the $\:i-$th curve. A larger value of $\:\stackrel{-}{{W}_{rel}}$ indicates that the DTW distance of the data-driven model is significantly lower than that of the reference model, implying a better fit to the experimental data. Moreover, the notation $\:\stackrel{-}{{W}_{rel}}\left(q,p\right)$ indicates that the discrepancy is evaluated based on the $\:q-p\:$plots.

### Influence of fine-tuning configurations

In transfer learning, to preserve knowledge acquired by the pre-trained model and to adapt to the scarcity of target data^57^, it is common practice to use its parameters as initial values while keeping some parameters fixed throughout the training process^50^, meaning some parameters are “frozen.” This section explores how the way of freezing parameters influence outcomes. To minimize confounding variables such as data volume, this analysis specifically highlights the effects of parameter freezing by expanding the test set to include 15 TMD tests (selecting three randomly per relative density level) and 8 TMU tests (four each from extension and compression experiments selected randomly). Additionally, this section compares the impact of different loss terms and batch sizes used during the fine-tuning process. Fine-tuning was conducted over 3000 epochs, with other parameter configurations maintained as per Table 1.

#### Freeze the first layer

**Fig. 8:**
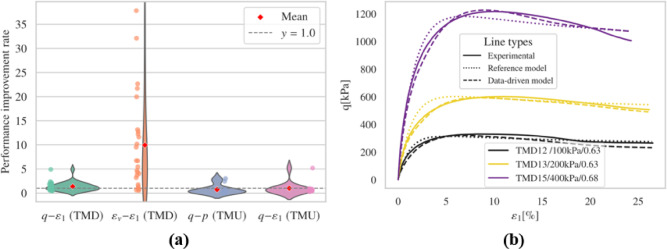
Results (**a**) overall performance; (**b**) representative results.

The results obtained from freezing the input layer’s weights and biases, followed by fine-tuning with experimental data, are shown in this section. For brevity, only representative cases and overall performance are presented in Fig. 8. Complete records are available in the supplementary material.

The fine-tuned model generally predicts the timing and magnitude of peak deviatoric stress more accurately than the reference model in drained triaxial simulations. Regarding dilatancy evaluation, the fine-tuned model exhibits notable improvement in capturing the general $\:{\epsilon\:}_{v}-{\epsilon\:}_{1}$ trend and the evolution of void ratio when compared to the reference model. However, irregular trends were noted in the $\:{\epsilon\:}_{v}-{\epsilon\:}_{1}$ plots for several simulations, including TMD 1, TMD 5, TMD 11, and TMD 16. Figs. S3 and S4 display the effective stress paths and stress-strain relationships for all undrained simulations. Compared to the reference model, the fine-tuned model achieves commendable results but produces unrealistic negative pressures in the TMU 7 and TMU 11 simulations, leading to a convergence problem.

#### Freeze the first two layers

Freezing the weight matrices and bias vectors of both the input layer and the first hidden layer, the fine-tuned model has only 91 adjustable parameters. The effects of considering only the loss $\:{E}_{r}$ associated with $\:dT$ and introducing additional error terms $\:{E}_{a}$ were explored, along with the impacts of batch size and learning rate adjustments. The model underwent 3000 epochs of training, maintaining other configurations as per Table 1.

##### Consider only $\:{E}_{r}$

**Fig. 9:**
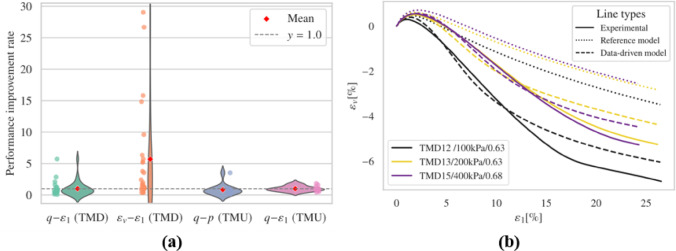
Results (**a**) overall performance; (**b**) representative results.

Figure 9 and Figs. S5-S6 show outcomes of drained test simulations. When considering only $\:{E}_{r}$, the model’s performance in predicting deviatoric stress under dense conditions and at higher confining pressures was not as accurate as the reference model. Although the fine-tuned model showed better volumetric behavior, it produced physically unrealistic rapid contraction behaviors at the end of loading for TMD 25.

The fine-tuned model outperforms the reference model in undrained simulations, particularly evident in the $\:q-p\:$relationships as depicted in Figure S6. Specifically, the fine-tuned model successfully captured limited flow, which is not effectively reproduced by the reference model. However, anomalies occurred in the TMU 11 simulation, where unrealistic tensile states ($\:p\:$< 0) led to non-convergence issues. Interestingly, in several simulations such as TMU 3, TMU 5, and TMU 6, the results closely aligned with the reference model, indicating that retaining more parameters from the pre-trained model can yield performances similar to the reference in certain aspects.

##### Consider only $\:{E}_{a}$

**Fig. 10:**
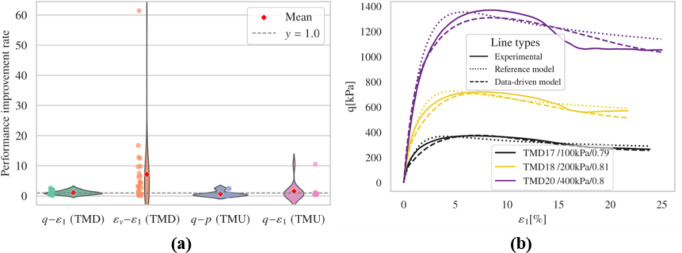
Results (**a**) overall performance; (**b**) representative results.

If only the loss component $\:{E}_{a}$ is included in the fine-tuning process, the outcomes are displayed in Fig. 10 and Figs. S9-S12. Overall, its visual performance exceed that of using only $\:{E}_{r}$. However, it does not encounter convergence issues, and the quantitative analysis pre sented in Sect. 4.1.4 reveals certain advantages.

##### Incorporate further loss items

**Fig. 11:**
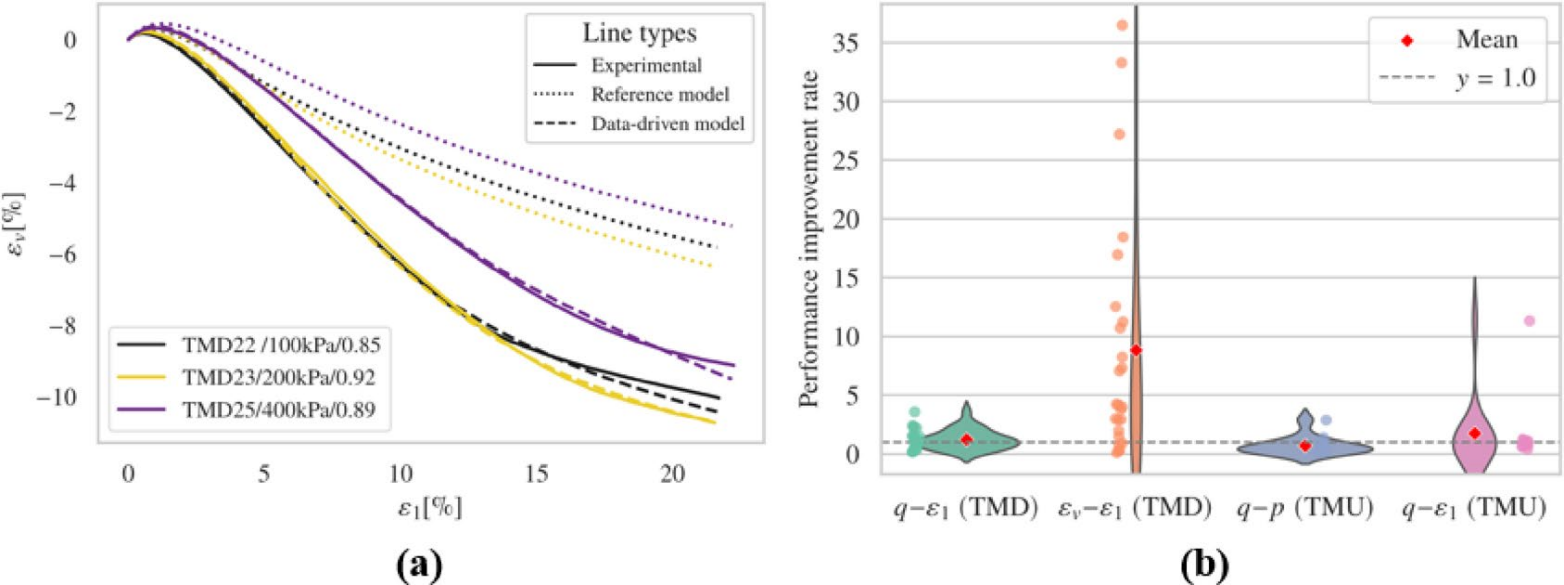
Results (**a**) representative results; (**b**) overall performance.

When additional loss term $\:{E}_{a}$ is included in the fine-tuning process, the outcomes are displayed in Fig. 11 and Figs. S13-S16.

The introduction of an additional loss term, $\:{E}_{a}$, in the fine-tuning process resulted in improved predictions of deviatoric stress, although the volumetric behavior in simulations of loose samples occasionally exhibited suboptimal performance. The undrained test results, as illustrated in Figs. S15 and S16, showed slight degradation; the $\:q-p$ curves effectively reflected initial stiffness but struggled to identify the phase transition points accurately, although they did reflect the critical state line. The deviatoric stress responses over the strain development were similar to those models considering only $\:{E}_{r}$, with several simulations aligning closely with the reference model. However, the inclusion of additional constraints contributed to greater stability, preventing convergence issues and physical inaccuracies.

##### Effect of batch size

**Fig. 12:**
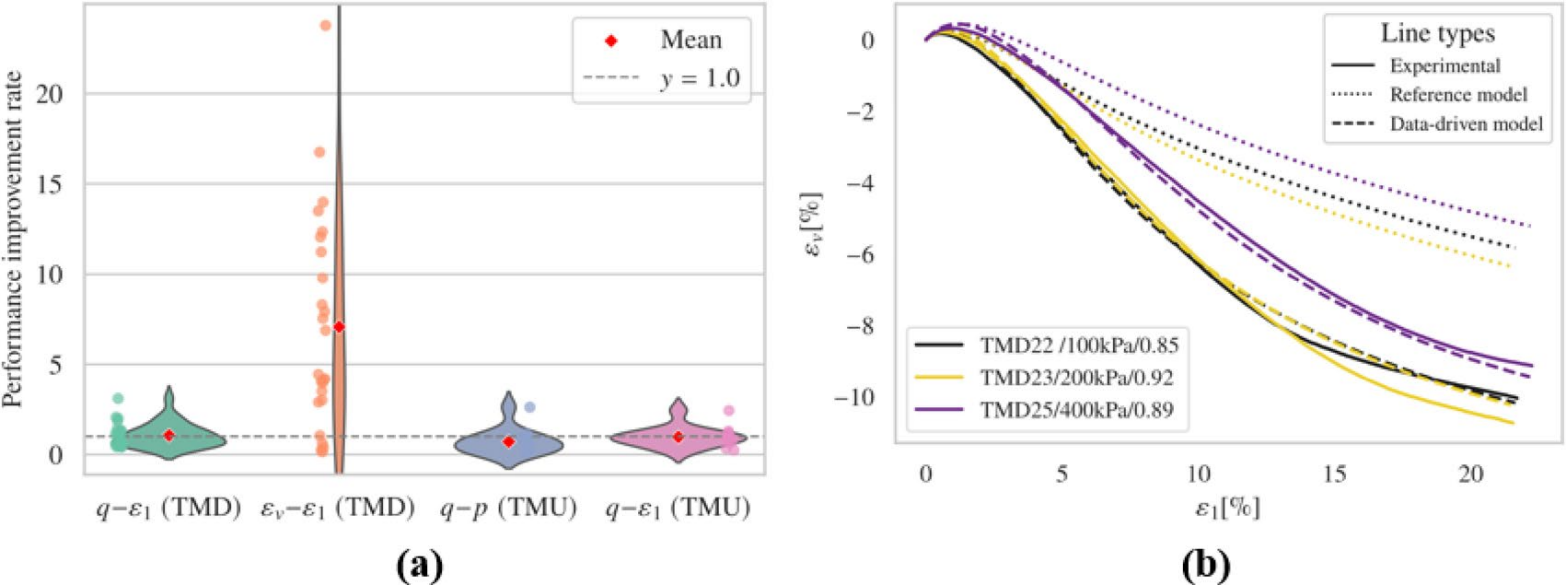
Results (**a**) overall performance; (**b**) representative results.

The choice of batch size influences MLP performance yet selecting the most suitable batch size and learning rate for optimal generalization remains unclear^58^. Typically, the effects of batch size and learning rate are discussed with respect to specific datasets^59^ or particular tasks^60^, which aids in building well-performing models. The interaction between batch size and learning rate is crucial^61^. Generally, a smaller batch size can help achieve global optima in clean data sets, which is why a smaller batch size of 32 was initially used for the calibration of the pre-trained model (refer to Table 1). In this section, a departure from the small batch size and learning rate listed in Table 1 is taken, opting instead for a larger batch size (2048) combined with a lower learning rate (1e-4), with the results depicted in Fig. 12 and Fig. S17-S20.

Following the introduction of the additional loss term $\:{E}_{a}$ and the use of a larger batch size with a smaller learning rate, the performance in drained test simulations was further improved. The behavior in undrained tests remained largely unchanged, still capturing initial stiffness well but failing to accurately capture phase transition points, although the critical state line was still evident.

#### Further checking on the architecture

Further checking was carried out by altering the network structure, including freezing more neurons and freezing all parameters of the pre-trained model while incorporating additional neural layers. New layers were added both before and after the original output layer, effectively reprocessing the $\:{p}_{i}$ parameters ($\:i$=1,2,3) of the reference model. Results indicated that freezing more parameters improved convergence but reduced the model’s fitting performance. Adding new layers worsened outcomes due to decreased flexibility, although this led to more stable convergence and smoother curves in finite element simulations. For brevity, detailed results are presented in the supplementary information.

Combining the findings from this section, it is clear that moderately freezing parameters (as demonstrated by freezing the first two layers), adopting more comprehensive constraints, and adjusting batch size and learning rate according to data quality can significantly enhance the overall performance of the model.

#### Performance comparisons

All models discussed in this section are compared based on their convergence ability under different boundary conditions, adherence to physical phenomena, and curve-fitting performance. The results are presented in Table 2.

**Table 2:** Evaluation of models with different fine-tuning configurations.

Models items	Reference model	Model 41 − 1 ($\:{E}_{r}+{E}_{a}$)	Model 41-2-1 ($\:{E}_{r}$)	Model 41-2-2 ($\:{E}_{a}$)	Model 41-2-3 ($\:{E}_{r}+{E}_{a}$)	Model 41-2-4 ($\:{E}_{r}+{E}_{a}$)
Description	Reference model	Model in Sect. 4.1.1	Model in Sect. 4.1.2.1	Model in Sect. 4.1.2.2	Model in Sect. 4.1.2.3	Model in Sect. 4.1.2.4
Extrapolation ability (convergence ability)						
Convergence ratio	1	35/37	36/37	1	1	1
Compliance with known physical phenomenon in all simulations						
Critical state	Yes	Yes	No	No	Yes	Yes
Limited flow in TMU	No	Yes	Yes	Yes	Yes	No
Phase transition in TMU	Yes	Yes	Yes	Yes	Yes	Yes
Other non-physical phenomena	/	Negative pressure	Negative pressure	/	/	/
Fitting ability (based on DTW distance)						
$\:\stackrel{-}{{W}_{rel}}\left(q,{\epsilon\:}_{1}\right)$ of TMD	1	1.35	1.00	1.11	1.21	1.06
$\:\stackrel{-}{{W}_{rel}}\left({\epsilon\:}_{v},{\epsilon\:}_{1}\right)$ of TMD	1	9.95	5.70	7.18	8.81	7.07
$\:\stackrel{-}{{W}_{rel}}\left(q,p\right)$ of TMU	1	0.74	0.83	0.70	0.74	0.71
$\:\stackrel{-}{{W}_{rel}}\left(q,{\epsilon\:}_{1}\right)$ of TMU	1	1.51	1.00	1.54	1.73	0.97

Overall, the fine-tuned model outperforms the reference model in drained simulations and generally provides improved predictions of the $\:q-{\epsilon\:}^{1}$ relationship under undrained conditions. However, it shows reduced accuracy when predicting the $\:q-p$ relationship under undrained conditions. Results indicate that using both $\:{E}_{r}$ and $\:{E}_{a}$ loss terms is more effective than using a single loss term, resulting in the most significant performance improvements compared to the reference model. Additionally, increasing the batch size brings the model’s performance closer to that of the reference model. Retaining more trainable parameters improves curve-fitting but adversely affects convergence. Therefore, in subsequent studies, the parameters of the first two layers are kept fixed, and both $\:{E}_{r}$ and $\:{E}_{a}$ loss terms are employed during training.

### Limit available data

To emphasize the assumption of limited laboratory data, the experimental setup was limited to five drained triaxial tests (TMD) and two undrained triaxial tests (TMU). Specific initial conditions for the drained tests were $\:\left({p}_{0},{I}_{d0}\right)$= (200 kPa, 0.21); (300 kPa, 0.55); (50 kPa, 0.57); (100 kPa, 0.79); (25: 400 kPa, 0.95). For the undrained tests, initial conditions were $\:\left({p}_{0},{I}_{d0}\right)$= (200 kPa, 0.64); (200 kPa, 0.53). This design involved multiple initial relative densities and confining pressure levels but only one sample per level, replicating the constraints found in real-world testing conditions. The undrained compression and extension tests (axial stretching) also occurred in only one set, covering a very limited range of variables, representing the practical scenarios where only a few tests might be conducted.

Two scenarios were considered: the first excluded any synthetic data from undrained simulations; the second used simulated undrained experiments using the reference model to supplement the drained experiment data records with stress-strain data obtained from simulations. Based on findings in the previous sections, this part of the model froze the first two layers and considered both $\:{E}_{r}$ and $\:{E}_{a}$ during the calibration process. Given the limited number of available drained experiment records, a larger batch size than in previous sections could not be employed; thus, a batch size of 960 was used, while keeping other parameters unchanged from earlier settings.

#### Without synthetic data

**Fig. 13:**
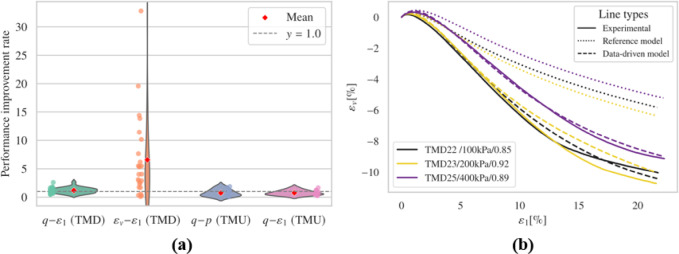
Results (**a**) overall performance; (**b**) representative results.

The results from the drained simulations (Fig. 13 and S21-S22) indicate that a significant reduction in available data somewhat negatively impacted model performance. While the development of deviatoric stress did not show substantial differences, this suggests that capturing shear stress in drained conditions may be comparatively less sensitive to data availability. However, the volumetric behavior was notably affected, with poor simulation outcomes under lower initial confining pressures (50 kPa). The undrained simulations (Figs. S23-S24) were also affected, with the most notable issues occurring in the triaxial extension tests involving loose samples, where excessive shear stress development was observed.

#### With synthetic data from undrained simulation

**Fig. 14:**
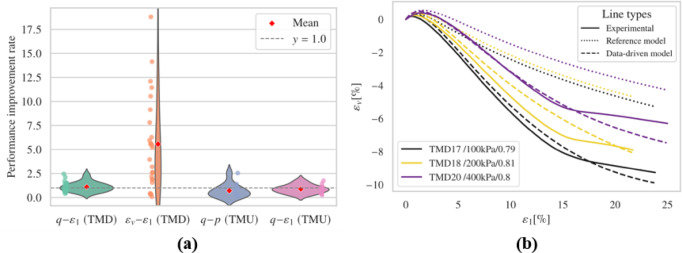
Results (**a**) overall performance; (**b**) representative results.

To address the excessive accumulation of shear stress observed in undrained triaxial extension simulations when only two undrained experimental tests were available, this section augmented the dataset with synthetic data. In addition to the two original undrained test records, ten extra undrained tests were simulated using the reference model. However, only data from the final portion of these simulations, namely the part where axial extension or compression strain exceeded 70% of its final value, was used to fine-tune the model. This approach aims to minimize the contamination of experimental data by low-fidelity synthetic data, while preserving informative samples that lie at or near the critical state. The results of this approach are presented in Fig. 14 and Figs. S25-S28.

Incorporating this additional undrained test data did not meaningfully affect the outcomes of the drained simulations, neither enhancing nor reducing the model’s performance in terms of deviatoric stress development or volumetric behavior.

For the undrained simulations, while the results of the undrained compression tests remained largely unchanged, the excessive growth of shear stress in the undrained extension tests was effectively alleviated. This adjustment highlights the potential benefits of selectively expanding the training dataset to address specific modeling challenges without substantially affecting the overall performance.

### Use only drained tests

This section investigates in more detail the implications of drastically limiting the available dataset for calibrating the PeNNs. In this scenario, the assumption is that merely five drained experimental records are available. Given the further reduction in available data, a batch size of 320 is selected to accommodate the smaller dataset.

#### Without synthetic data

**Fig. 15:**
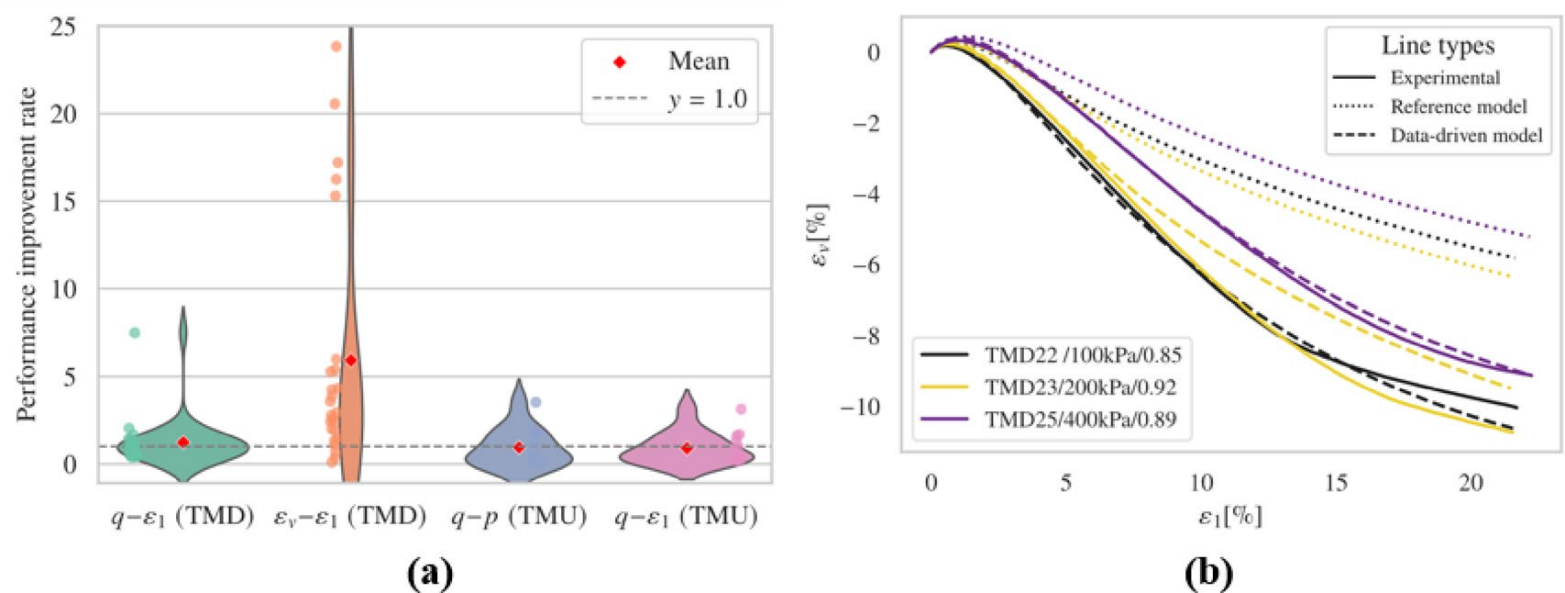
Results (**a**) overall performance; (**b**) representative results.

When only five drained experimental records are available for fine-tuning the models, the results of the triaxial test simulations are shown in Fig. 15 and Figs. S29-S32. The simulation outcomes for drained tests are comparable to the results in the previous section, indicating that excluding undrained experimental data does not significantly impact the simulation of drained tests, especially regarding volumetric behavior. However, the prediction of shear stress was negatively impacted, showing a reduction in performance under conditions of higher initial confining pressure.

In terms of undrained simulations, despite the absence of undrained experimental records in the fine-tuning dataset, the simulations of undrained compression tests still performed well. Nevertheless, the simulations of undrained extension tests showed unphysical shear strength loss when axial extension was significant, highlighting the necessity of including drained extension test data for accurate modeling.

#### With synthetic data of undrained test

Similar to the approach detailed in Sect. 4.2.2, this section incorporates synthetic data generated by undrained simulations to fine-tune the PeNNs.

**Fig. 16:**
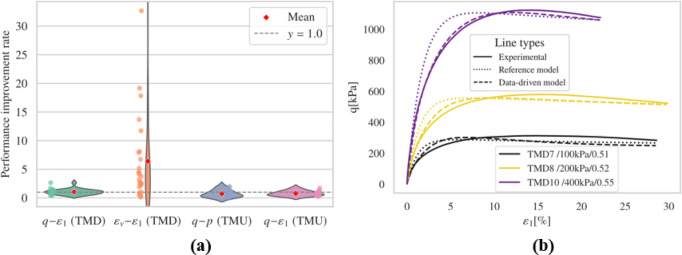
Results (**a**) overall performance; (**b**) representative results.

The outcomes of the drained test simulations are depicted in Fig. 16 and Figs. S33-S34. The introduction of synthetic undrained data has significantly enhanced the performance of the model in predicting deviatoric stress in drained tests. However, there were no significant changes observed in volumetric behavior.

The simulation results for undrained tests, as shown in Figs S35-S36, indicate a clear improvement compared to models trained solely on drained experiment data. The inclusion of even partial undrained test data strongly improves the simulation results for undrained extension tests, closely matching the experimental trends. This confirms that synthetic data can effectively supplement limited real data. This is particularly beneficial in scenarios where experimental data are are limited or expensive, enabling robust model performance even with very limited real-world data.

### Influence of data in fine-tuning process

To investigate the impact of the available experimental data volume and the incorporation of synthetic data on the fine-tuned models, we compared models fine-tuned with different datasets under identical conditions. Specifically, we analyzed the models presented in Sect. 4.1.2.3, Sect. 4.2.1 and 4.2.2, and Sect. 4.3.1 and 4.3.4 and summarized the results in Table 3.

**Table 3:** Evaluation of models with different fine-tuning data.

Models items	Reference model	Model 41-2-3	Model 4-2-1	Model 4-2-2	Model 4-3-1	Model 4-3-2
Description	Reference model	Section 4.1.2.3 (15 TMD + 8 TMU + Synthetic data)	Section 4.2.1 (5 TMD + 2 TMU)	Section 4.2.2 (5 TMD + 2 TMU + Synthetic data)	Section 4.3.1 (5 TMD)	Section 4.3.2 (5 TMD + Synthetic data)
Extrapolation ability (convergence ability)						
Convergence ratio	1	1	36/37	1	31/37	1
Compliance with known physical phenomenon in all simulations						
Critical state	Yes	Yes	Yes	Yes	No	Yes
Limited flow in TMU	No	Yes	No	No	No	No
Phase transition in TMU	Yes	Yes	Yes	Yes	No	Yes
Other non-physical phenomena	/	/	/	/	Collapse	/
Fitting ability (based on DTW distance)						
$\:\stackrel{-}{{W}_{rel}}\left(q,{\epsilon\:}_{1}\right)$ of TMD	1	1.21	1.18	1.11	1.21	1.04
$\:\stackrel{-}{{W}_{rel}}\left({\epsilon\:}_{v},{\epsilon\:}_{1}\right)$ of TMD	1	8.81	6.57	5.57	5.91	6.41
$\:\stackrel{-}{{W}_{rel}}\left(q,p\right)$ of TMU	1	0.74	0.75	0.71	0.94	0.73
$\:\stackrel{-}{{W}_{rel}}\left(q,{\epsilon\:}_{1}\right)$ of TMU	1	1.73	0.76	0.88	0.89	0.78

As shown in Table 3, a reduction in available experimental records visibly diminishes the performance of the fine-tuned models. Introducing synthetic data appropriately can mitigate this decline. When the available experimental data includes only a minimal number of undrained test records, the model’s simulation performance deteriorates, exhibiting issues such as non-convergence, inability to capture limited flow behavior, and reduced fitting accuracy. If the available data contains no undrained test records, the model’s convergence substantially worsens, fails to accurately reflect the critical state, and even produces unrealistic collapse behavior in undrained extension simulations.

However, incorporating synthetic data during the fine-tuning process can enhance model performance. For instance, Model 4.3.1, which does not include any synthetic or experimental data related to undrained behavior, is unable to simulate phase transition point in undrained extension simulation, as clearly demonstrated in the resulting figures. This indicates poor extrapolation capabilities. However, quantitative analysis reveals that Model 4.3.1 performs better in undrained conditions compared to the model discussed in Sect. 4.3.2, which includes synthetic undrained data. Even when using an alternative similarity metric, the Fréchet distance, Model 4.3.1 outperforms the model 4.3.2 (0.87:0.77 for $\:q-p$ plots and 0.72:0.72 for $\:q-{\epsilon\:}_{1}$ plots). However, this quantitative superiority does not correlate with the actual performance observed.

These comparisons highlight that relying solely on quantitative metrics for fitting ability is insufficient. It is essential to first evaluate the convergence behavior of FEM simulations and the adherence to physical phenomena. In scenarios with limited experimental data, the introduction of synthetic data during fine-tuning can enhance both the convergence performance and the physical accuracy of the models.

## Further discussion

### Insights on fine-tuning configurations

From a model perspective, maintaining only a subset of the network’s parameters trainable during fine-tuning focuses optimization on these active parameters. This approach requires these retained parameters to compensate for the discrepancies between conventional reference models and experimental data. Consequently, the sensitivity of the model to these parameters significantly increases, with their performance directly influencing the convergence and overall performance of the model during boundary value problem simulations.

From a data perspective, the experimental data is inevitably noisy. Employing a small batch size tends to increase the impact of the last few batches’ data quality during the fine-tuning process. Consequently, although using a smaller batch size can enhance model performance, it sometimes results in outputs that violate physical laws due to data quality issues. In contrast, employing a larger batch size can speed up training and improve stability in BVPs, albeit sometimes at the cost of reduced curve-fitting accuracy. Therefore, if the quality of experimental data is exceptionally high, a smaller batch size can facilitate superior model performance. Conversely, in the presence of significant noise, a larger batch size can contribute to more stable model performances.

Of course, both the combinations of experimental data and the model parameters are subject to uncertainty. However, elucidating the underlying mechanisms is technically challenging. For example, selecting 10 tests out of 25 experimental datasets yields $\:C\left(\text{25,10}\right)\:$= 3,268,760 potential combinations. Additionally, when employing Bayesian inference to update neural network parameters, the large number of parameters often results in a extremely small likelihood values, making sampling computationally infeasible. Future research will be necessary to address and refine such analyses.

### Model slicing

**Fig. 17:**
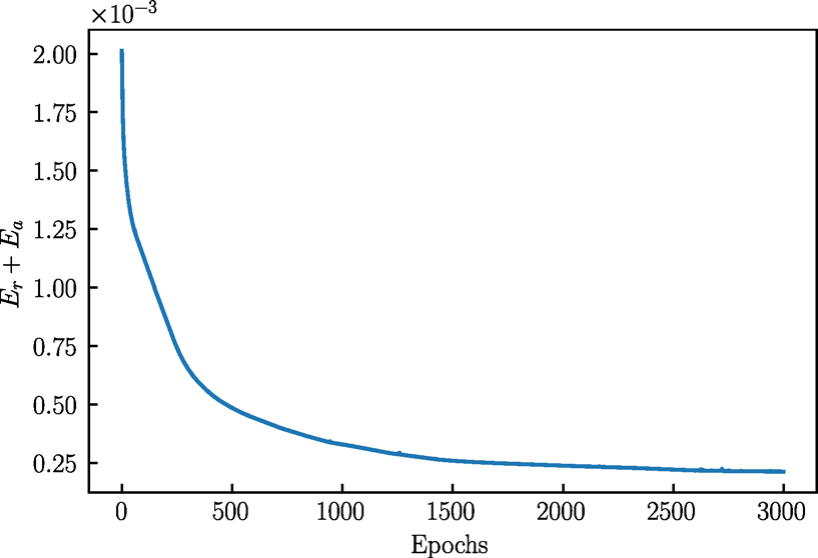
Fine-tuning loss over epochs.

To highlight the necessity of evaluating a data-driven constitutive model in the form of BVPs and examine the convergence speed of the fine-tuning process, the performance of a model (configuration from Sect. 4.2.2) was assessed at various fine-tuning epochs. This involved a detailed evaluation of the parameter calibration process across different slices of epochs. The evolution of the loss over epochs is shown in Fig. 17, demonstrating the effectiveness of the training.

Model parameters were extracted at the 10, 50, 150, 500, 1000, and 2000 epoch marks for simulations. From the $\:q-{\epsilon\:}_{1}$, $\:{\epsilon\:}_{v}-{\epsilon\:}_{1}$ relations in drained simulations, one set from each was selected for display along with the $\:q-p$ relation from the undrained tensile tests.

**Fig. 18:**
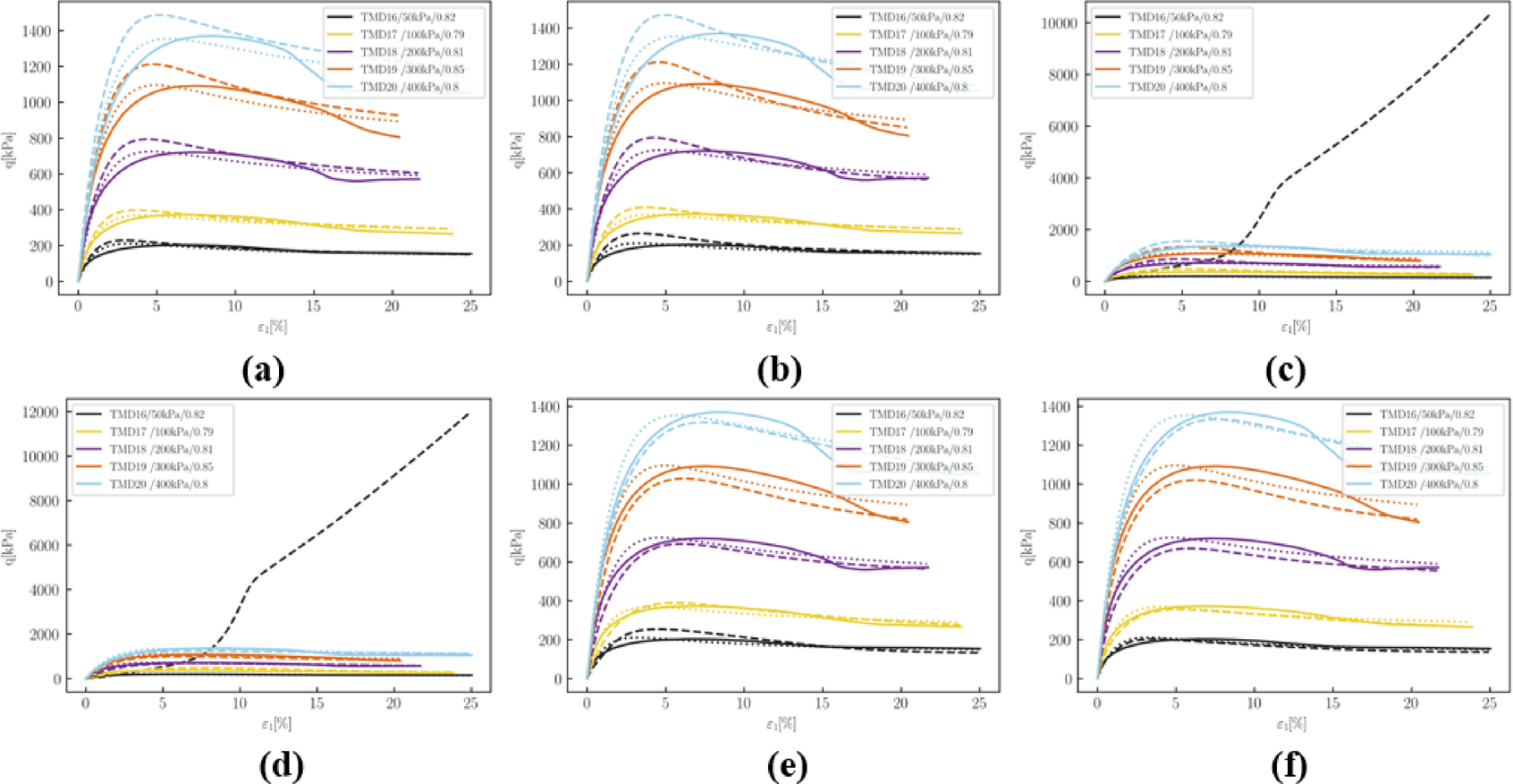
Deviatoric stress q versus axial strain $\:{\epsilon\:}_{1}$in drained simulations: (**a**) 10 epochs; (**b**) 50 epochs; (**c**) 150 epochs; (**d**) 500 epochs; (**e**) 1000 epochs; (**f**) 2000 epochs.

Figure 18 illustrates that at 150 and 500 epochs, the simulated deviatoric stress for TMD 16 exhibited abnormal developments. Peak deviatoric stresses of drained simulations tend to be overestimated before the deviation in TMD 16 while slightly underestimated after correction.

**Fig. 19:**
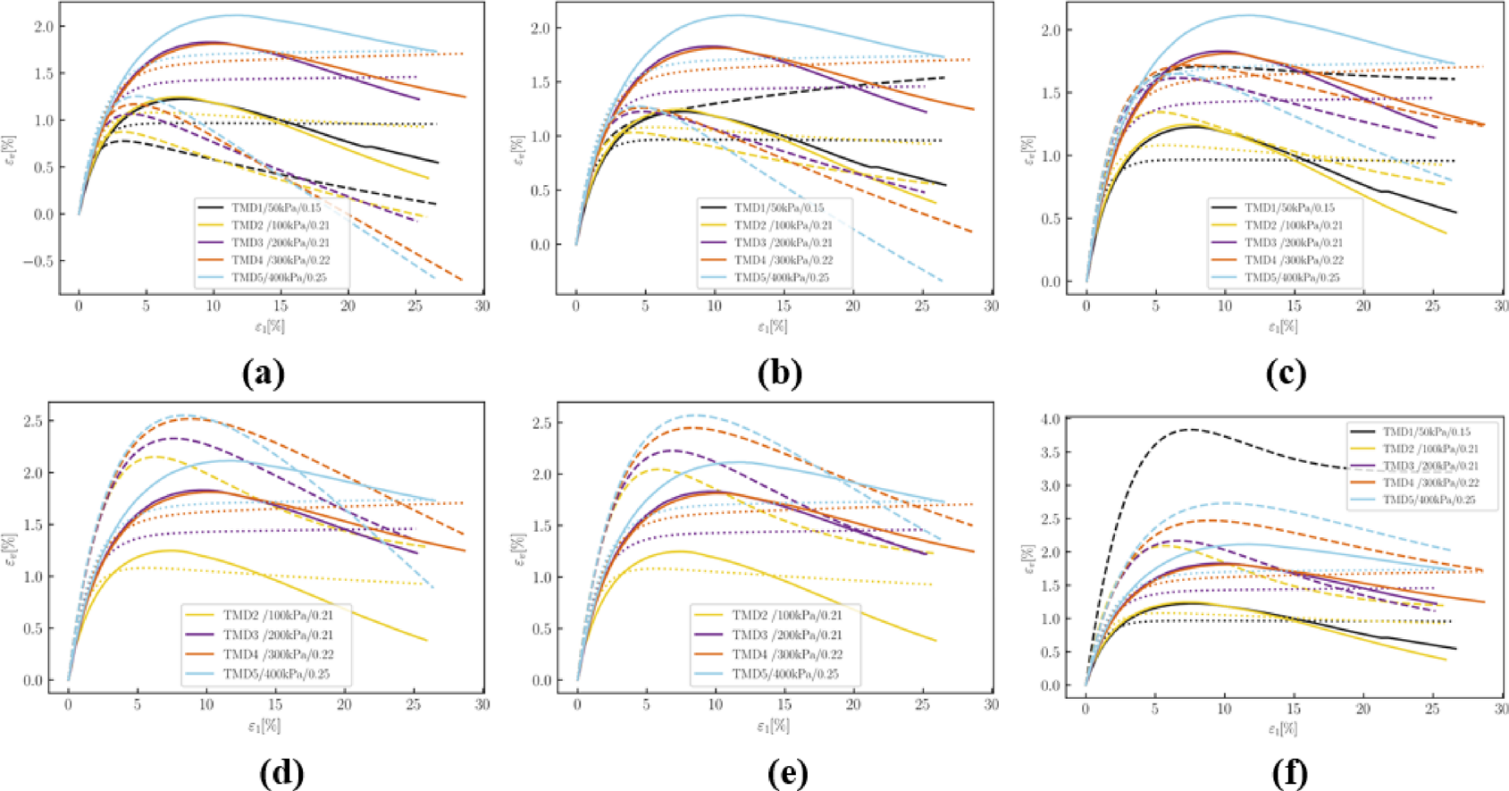
Volumetric strain $\:{\epsilon\:}_{v}$ versus axial strain $\:{\epsilon\:}_{1}$in drained simulations: (**a**) 10 epochs; (**b**) 50 epochs; (**c**)150 epochs; (**d**) 500 epochs; (**e**) 1000 epochs; (**f**) 2000 epochs.

Regarding volumetric behavior, at the 500 and 1000 epochs, the parameters did not successfully make the simulation for TMD 1 converge, thus no results are displayed. Figure 19 indicates that corrections in volumetric relationships are slower than those in shear stress. Except for TMD 1, simulations quickly approach experimental data.

**Fig. 20:**
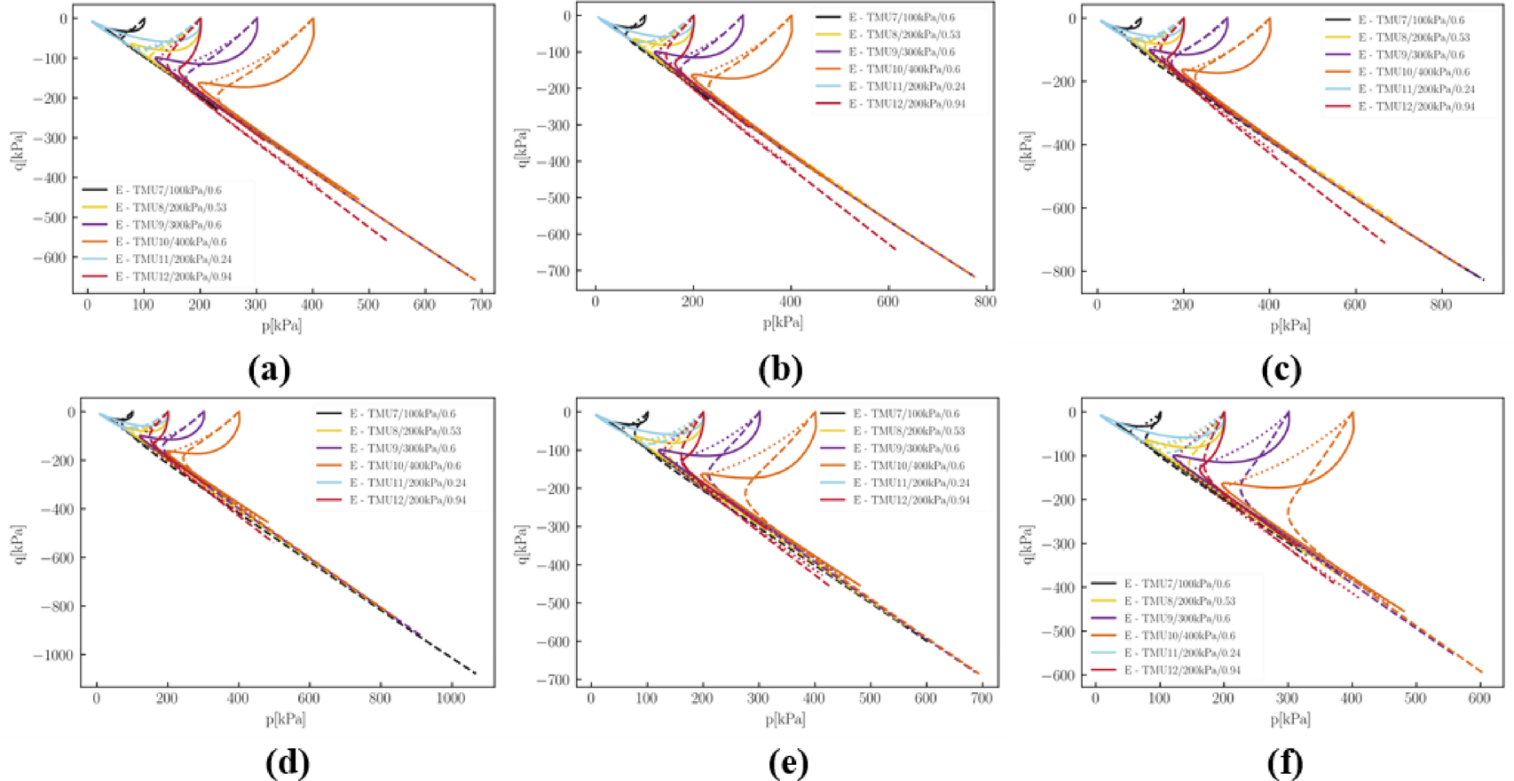
Volumetric strain $\:{\epsilon\:}_{v}$ versus axial strain $\:{\epsilon\:}_{1}$in drained simulations: (**a**) 10 epochs; (**b**) 50 epochs; (**c**) 150 epochs; (**d**) 500 epochs; (**e**) 1000 epochs; (**f**) 2000 epochs.

Figure 20 shows that the evolution of the $\:q-p$ relationship in undrained tensile tests is more complex. The development of deviatoric stresses does not consistently improve. To slow down the development of deviatoric stress, the pressure level at the phase transition point was incrementally increased.

The results indicate that although the loss term consistently decreased, this does not necessarily reflect improving model convergence or stability. It is reasonable since, at epoch 0, corresponding to the pre-trained model, both convergence ability and stability are quite satisfactory. This implies that to ensure good convergence, the model parameters should either remain close to those of the pre-trained model with fewer training epochs or sufficient corrections guided by experimental data.

Therefore, the evolution of loss during model calibration is not directly correlated with performance during simulations. This necessitates further study on effective calibration of data-driven models.

### Non-symmetric cases

The FEM simulations of drained and undrained tests are already extrapolation application because the stress state and loading increment cannot match exactly with the training data. However, we will further consider stress states and loading conditions far different from training data herein.

**Fig. 21:**
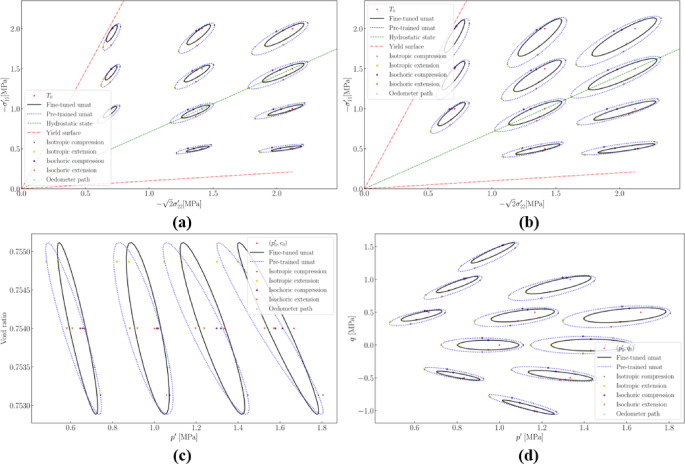
Response envelops in (**a**) principal stresses space (relative density = 0.3); (**b**) principal stresses space (relative density = 0.8); (**c**) $\:e-p$ space (relative density = 0.8); (**d**) in $\:q-p{\prime\:}$ space (relative density = 0.8).

When experimental data are highly limited, selectively incorporating data that contains specific physical information can enhance the performance of a fine-tuned model. For example, in Sect. 4.3.2, which utilizes the minimal amount of experimental data, the model’s extrapolation capability is further evaluated by employing a response envelope to thoroughly examine its responses within the $\:\left(p,\:q,\:e\right)$ state space. For a deformation probe with unitary length and a polar angle $\:{\alpha\:}_{D}$ ranging from 0 to 2π, the trace of the final state after applying this loading is plotted as a response envelope, as shown in Fig. 21.

Initially, we quantitatively assess the quality of the fine-tuned model. For any initial $\:(T,\:e)$ state, the response envelopes of both the pre-trained and fine-tuned models in the$\:\:(p,\:q)$, $\:(p,\:e)$, and principal stress spaces exhibit no intersections or distortions. This indicates that responses to different loading directions from the same $\:(T₀,\:e₀)$ state do not overlap, ensuring stable computation of strain increments under stress-controlled and mixed-controlled boundary conditions. The presence of intersections or distortions, such as pinching, would result in unsolvable or unstable solutions. Additionally, comparisons with the yield surface demonstrate that the fine-tuned model adheres to fundamental physical laws; for states at or near yield in the principal stress space, the post-loading stress state in any direction does not surpass the yield surface.

Comparative analysis reveals different responses between the fine-tuned and pre-trained models, indicating that the fine-tuning process effectively modifies the model’s behavior. Note that the strain increment used for plotting these figures, $\left\| {\Delta \epsilon } \right\|$, is relatively large, which magnifies the differences between the models. A closer inspection shows that the discrepancies primarily occur in the extension region, such as isotropic extension and associated directions, where the models differ the most. However, such scenarios are rare, and for granular materials like sand, which lack cohesion, isotropic extension is unlikely to occur in practice.

## Conclusion

In the development of data-driven constitutive models, obtaining appropriate initial values for model parameters is crucial, particularly in the absence of adequate experimental data and the explicitly defined target outputs (e.g. $\:{p}_{i}$ in this study) from the PeNN model. This study employs a transfer learning approach to efficiently develop data-driven constitutive models under conditions of limited experimental data. This method utilizes extensive synthetic data to provide comprehensive initial coverage, ensuring the model is well-informed across diverse conditions before incorporating experimental data for further refinement. During the model development process, UMATs were implemented in Abaqus, and hundreds of simulations were conducted to systematically evaluate these models. A set of evaluation frameworks for granular constitutive models based on data-driven methods was proposed and utilized. The findings can be concluded:

1. The performance of a data-driven constitutive model must ultimately be verified through finite element simulations. Although loss values and fitting accuracy during training may look satisfactory, they do not always reflect how well the data-driven constitutive model performs in actual boundary value problems. Since both conventional and data-driven constitutive models can meet converge problems in FEM simulation, a model that fits training data well may still fail to converge or produce physically unrealistic results during FEM simulations. Therefore, before evaluating quantitative fitting metrics, it is essential to examine whether the model can handle different loading conditions and reproduce key physical behaviors observed in experiments, such as critical state, limited flow, and phase transition.
2. The method of parameter freezing, the richness and representativeness of data available for fine-tuning, and the configuration of hyperparameters during this process will influence the performance of the fine-tuned model. Properly introducing synthetic data, freezing more parameters from pre-trained model and using larger batch size can improve simulation convergence, but may bring negative effect to fitting ability. In practice, selecting an appropriate configuration requires balancing data availability, training cost, model accuracy, and the convergence performance of FEM simulations.
3. Generally, if the experimental data are clean enough and of high quality, the PeNN model does not require extensive data to accurately reproduce the mechanical response. Conversely, if the quality of experimental data is poor, high-quality reference models can generate surrogate data as a compensation. Through meticulous fine-tuning, even a small amount of experimental data can effectively drive a PeNN-based constitutive model. Fine-tuning should employ robust loss information and larger batch sizes to balance model performance and stability.

While experimental curves provide valuable insights, they do not directly represent material point behavior, as they are influenced by factors such as sample heterogeneity, boundary conditions, loading modes, and strain localization. A physically robust constitutive model should ideally decouple these structural and experimental effects from intrinsic material behavior. Developing such models remains an important direction for future research. It should be also noted that the performance described does not reflect the upper limits of this framework. Employing neural networks with more parameters and conducting more detailed hyper-parameter tuning could potentially enhance the performance.

## Supplementary Information

Below is the link to the electronic supplementary material.

Supplementary Material 1

## Data Availability

The datasets analyzed during the current study are available in https://www.torsten-wichtmann.de/. The original publication is available at http://link.springer.com (DOI: 10.1007/s11440-015-0402-z.
