# Supplementary material for: Practical sparse data-driven constitutive modeling via transfer learning in physics-encoded neural networks
Source: Sci Rep. 2026 Jan 5;16:636. doi: 10.1038/s41598-025-34925-0 (PMC12775455; doi:10.1038/s41598-025-34925-0)
Supplement: Supplementary file 1 — Supplementary Material 1 [file 41598_2025_34925_MOESM1_ESM.docx]

## Supplementary material

### Results of Section 4.1.1


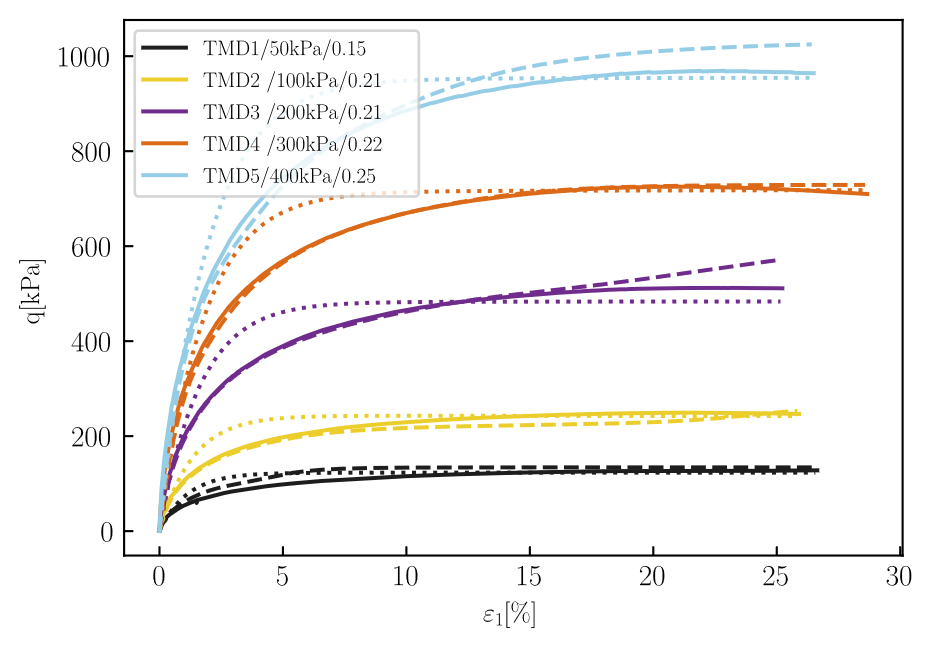

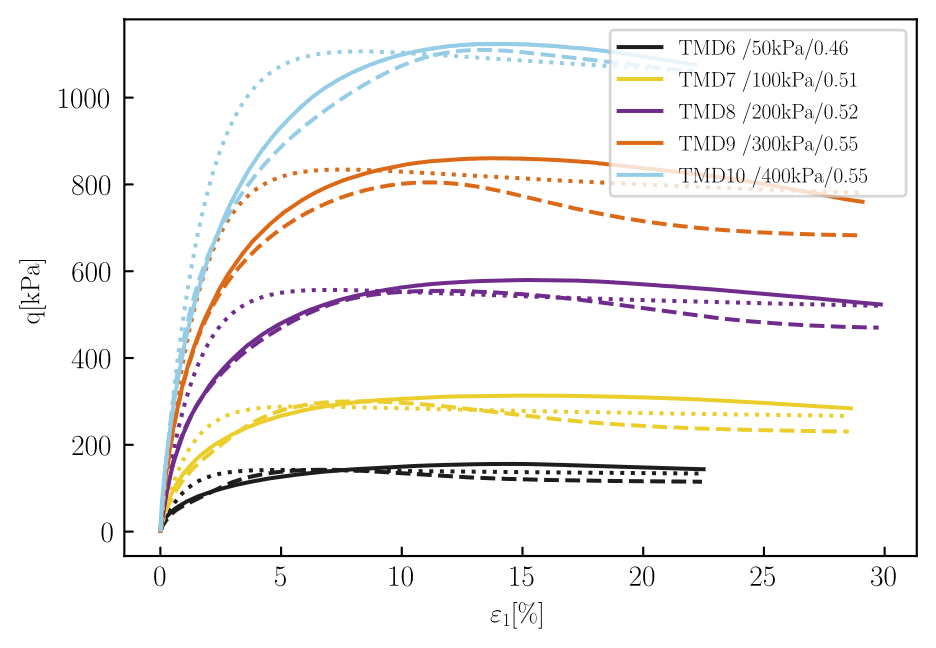

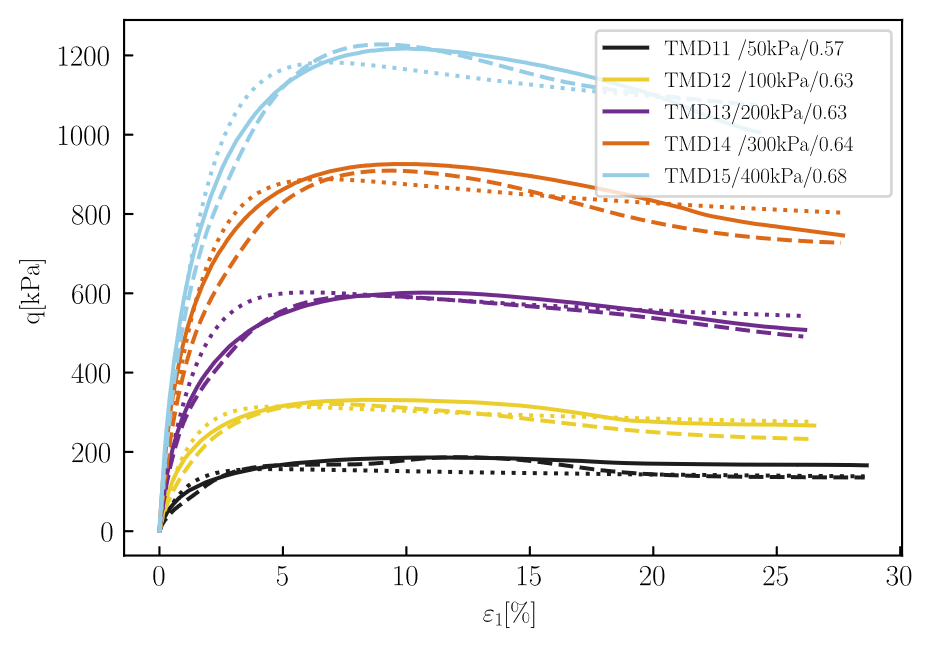


**(a)** **(b)** **(c)**


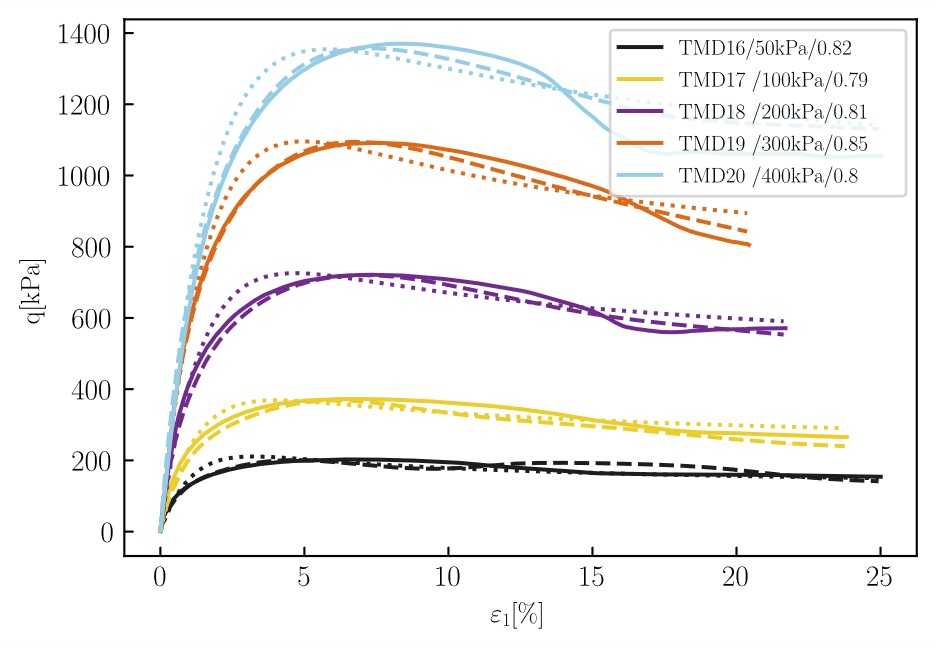

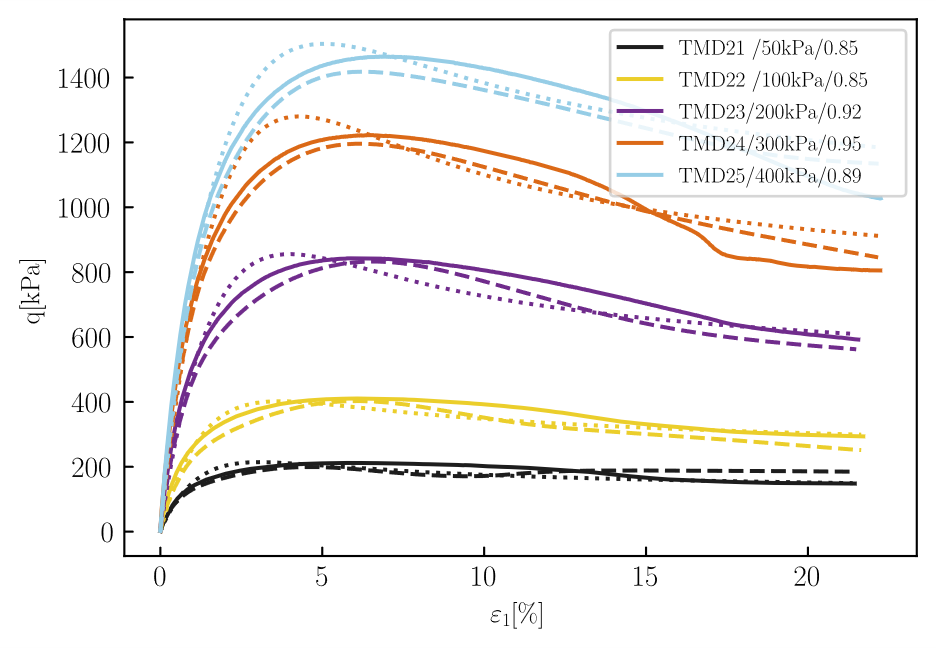


**(d)** **(e)**

**Fig. S1.** Deviatoric stress$q$ versus axial strain $\varepsilon_{1}$in drained simulations: (a) loose samples; (b) loose to medium dense samples; (c) medium dense samples; (d) medium dense to dense samples; (e) dense samples


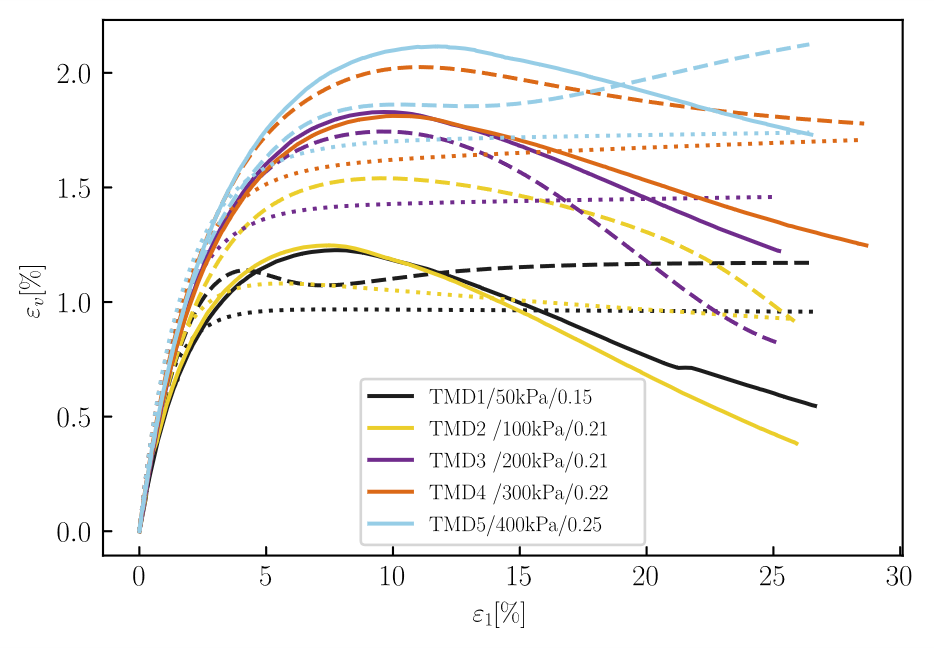

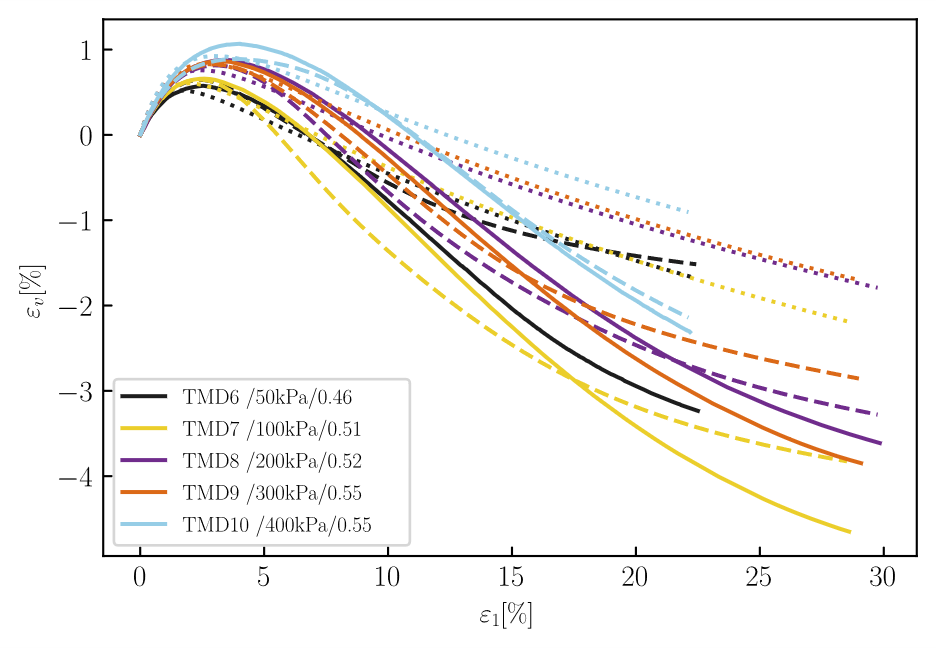

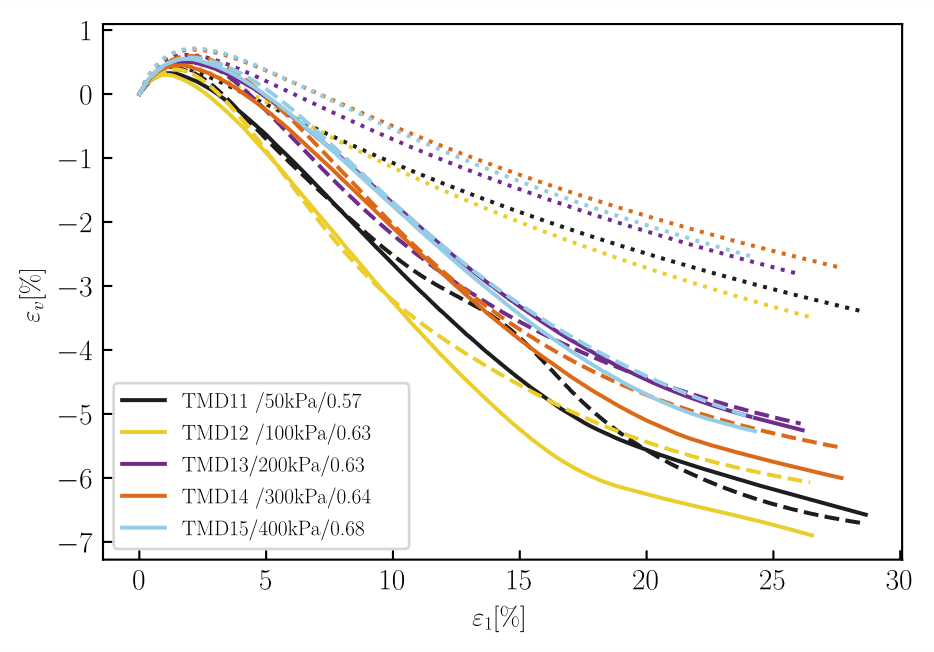


**(a)** **(b)** **(c)**


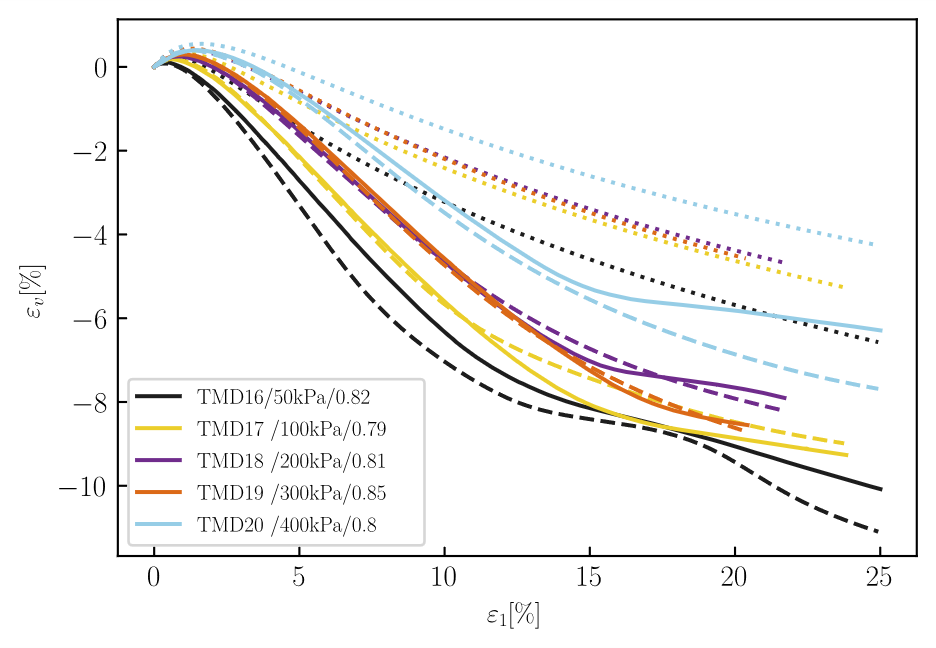

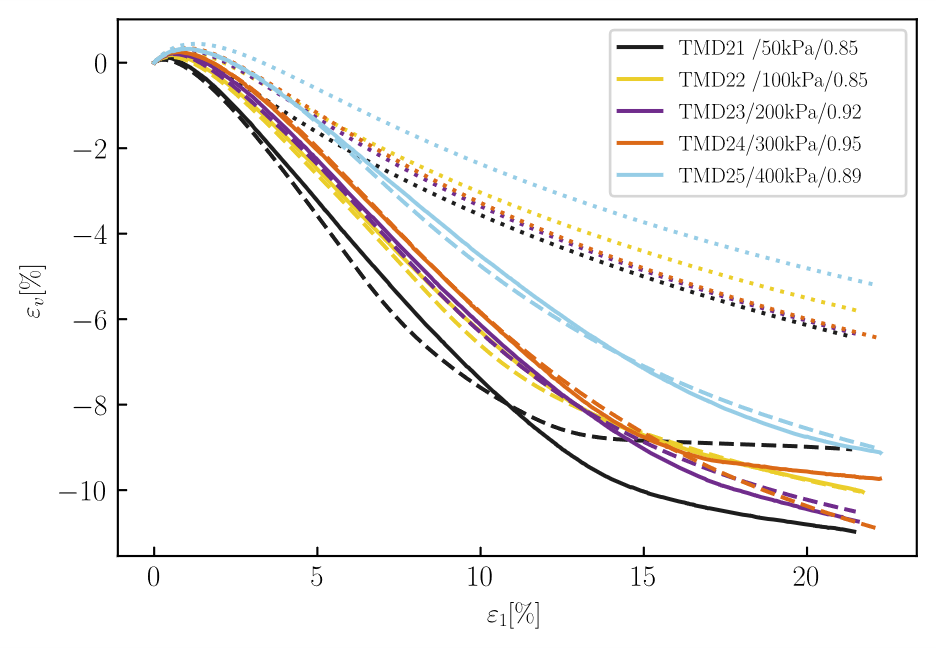


**(d)** **(e)**

**Fig. S2.** Volumetric strain $\varepsilon_{v}$ versus axial strain $\varepsilon_{1}$in drained simulations: (a) loose; (b) loose to medium dense; (c) medium dense; (d) medium dense to dense; (e) dense samples


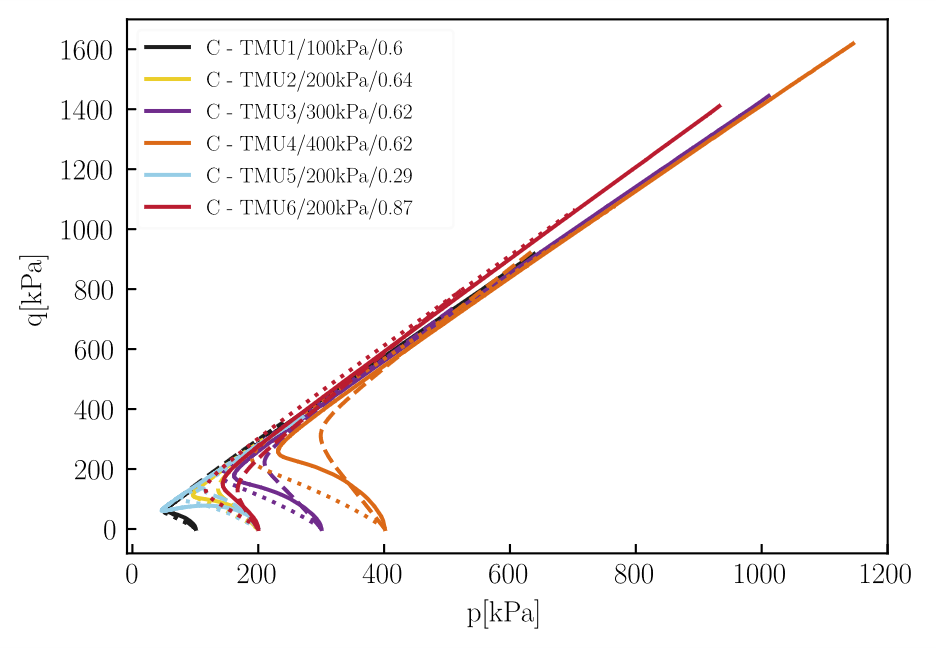

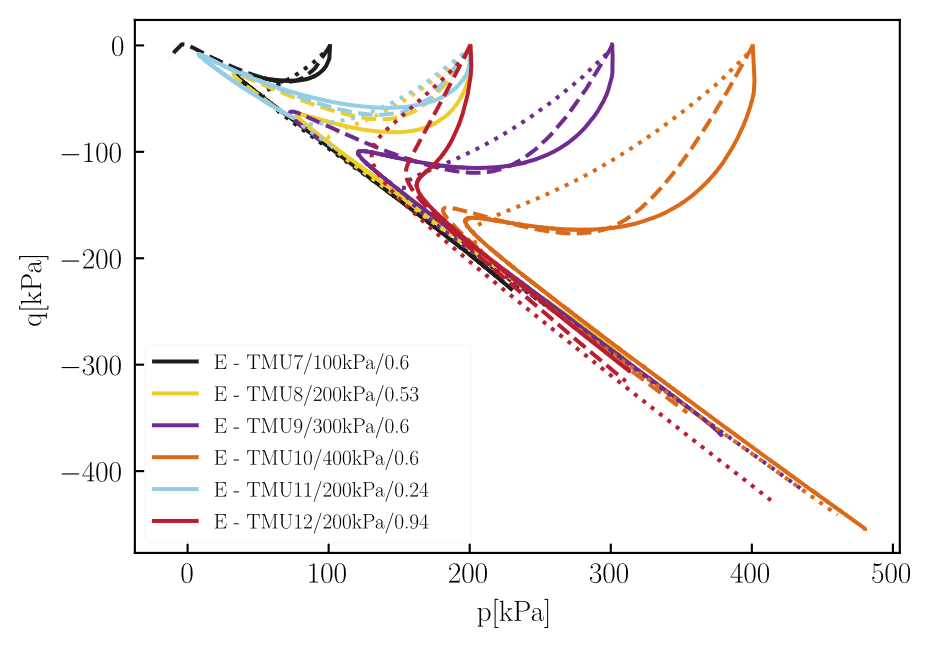


**(a)** **(b)**

**Fig. S3.** Effective stress paths in undrained simulations: (a) compression; (b) extension


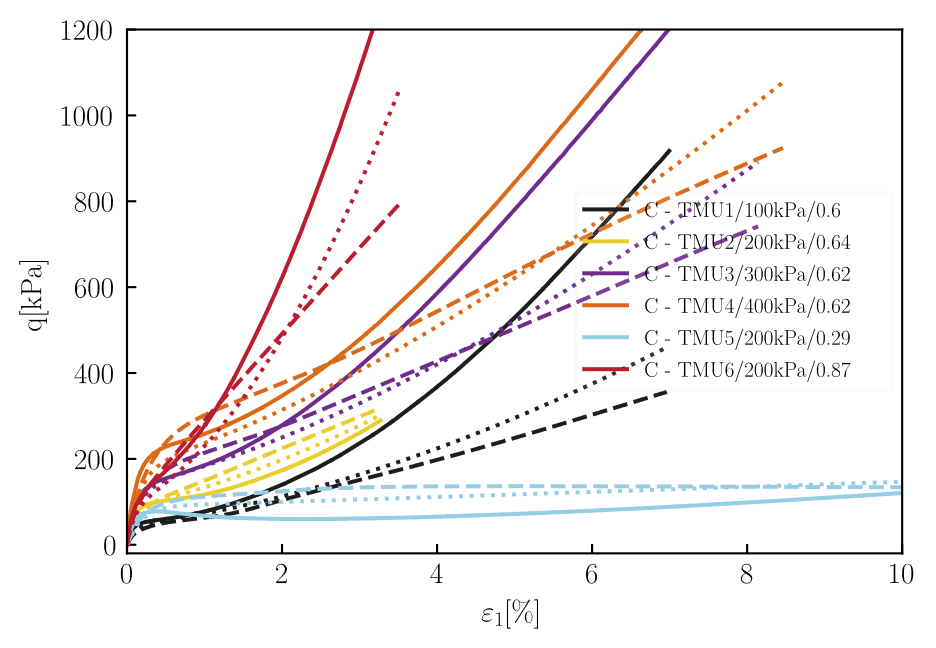

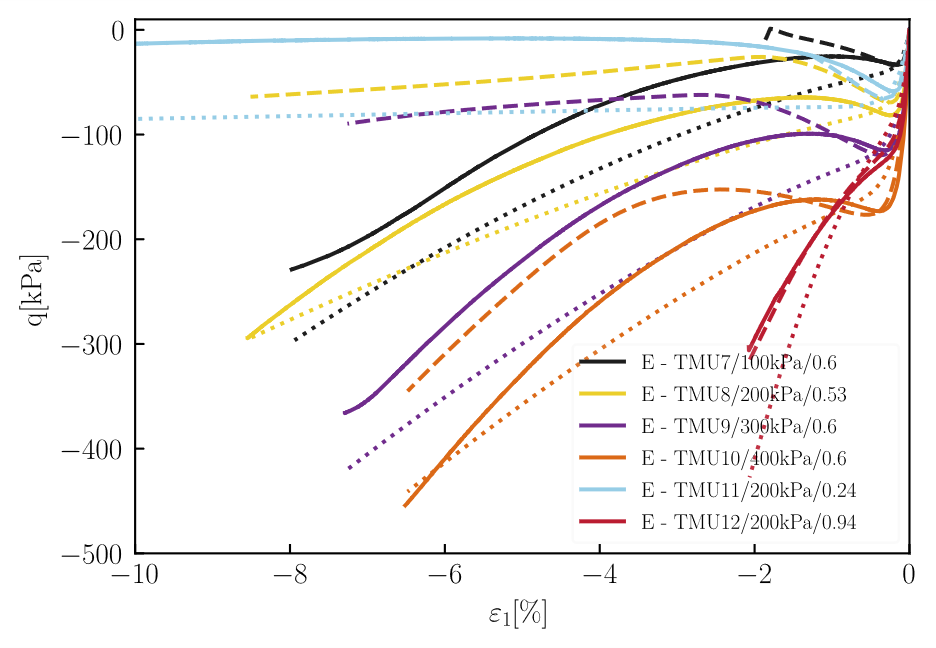


**(a)** **(b)**

**Fig. S4.** Stress-strain relationships in undrained simulations: (a) compression; (b) extension

### Results of Section 4.1.2.1


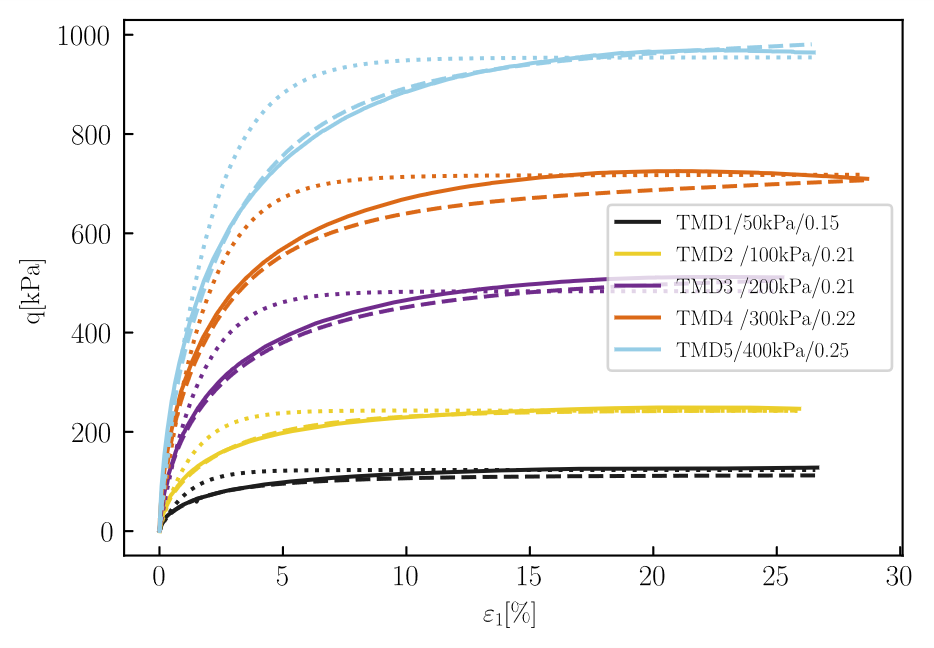

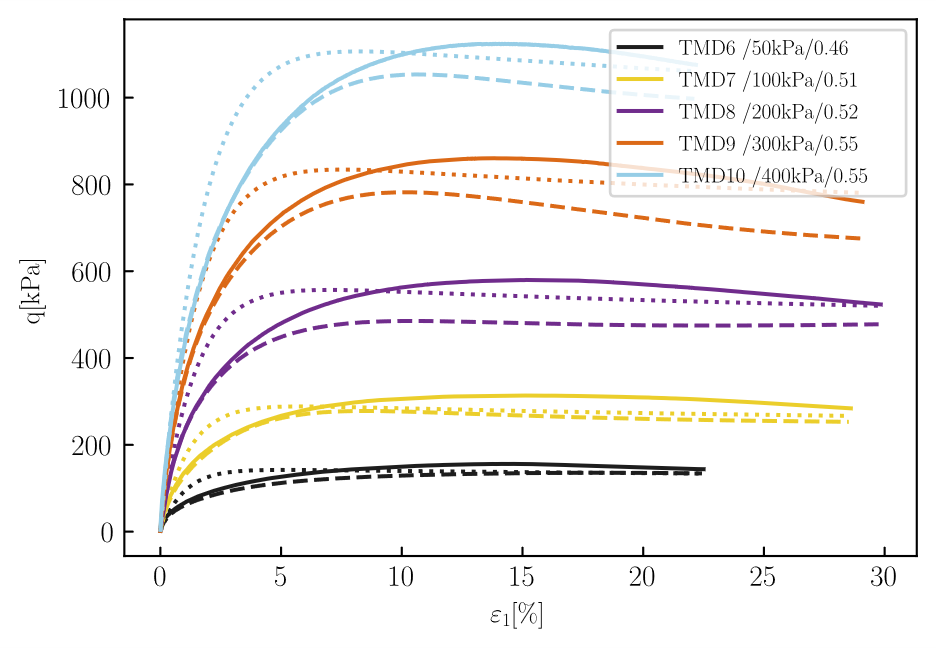

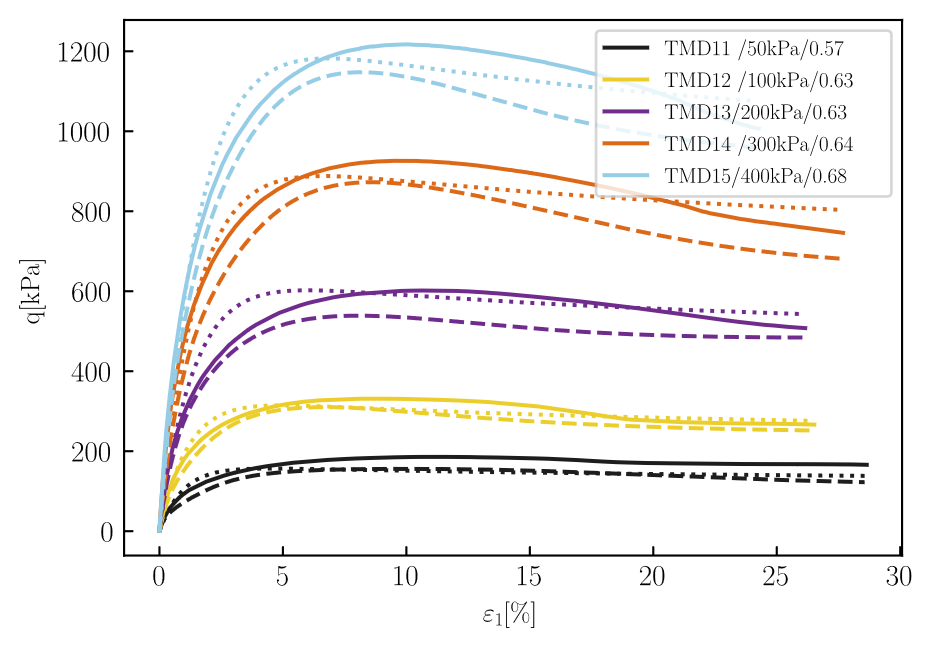


**(a)** **(b)** **(c)**


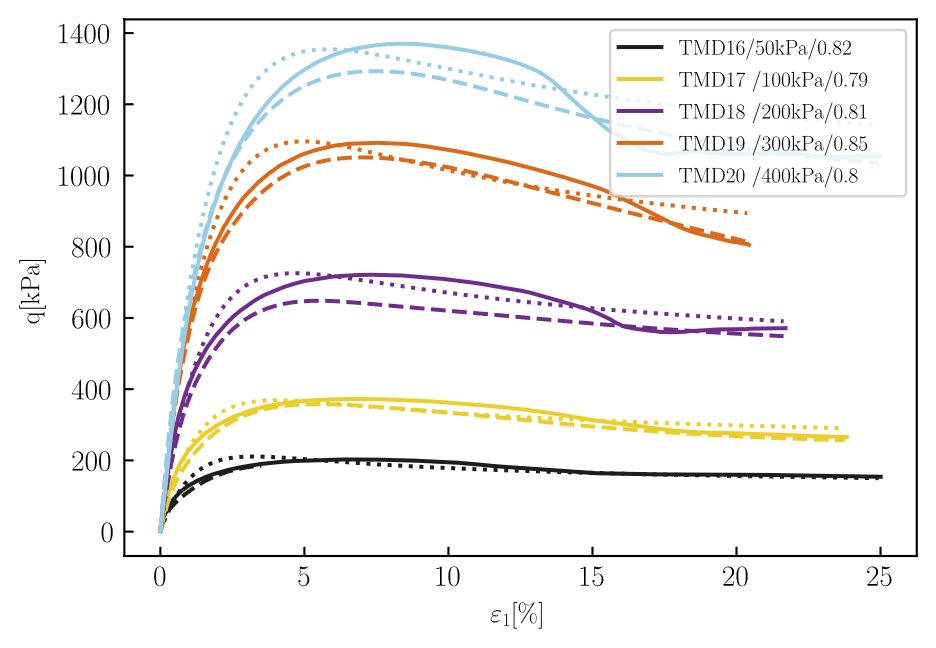

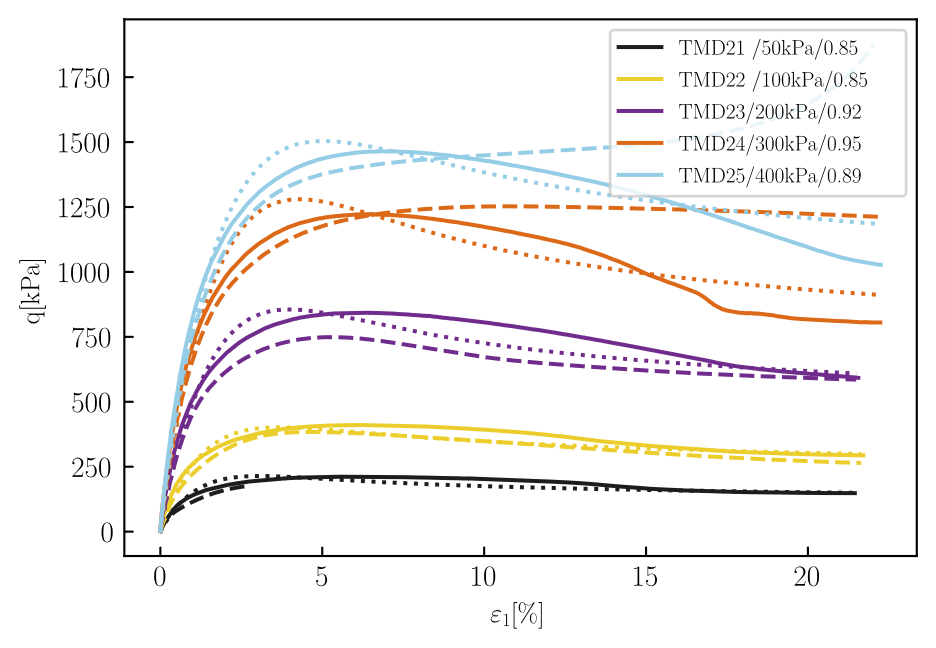


**(d)** **(e)**

**Fig. S5.** Deviatoric stress responses in drained simulations: (a) loose; (b) loose to medium dense; (c) medium dense; (d) medium dense to dense; (e) dense samples


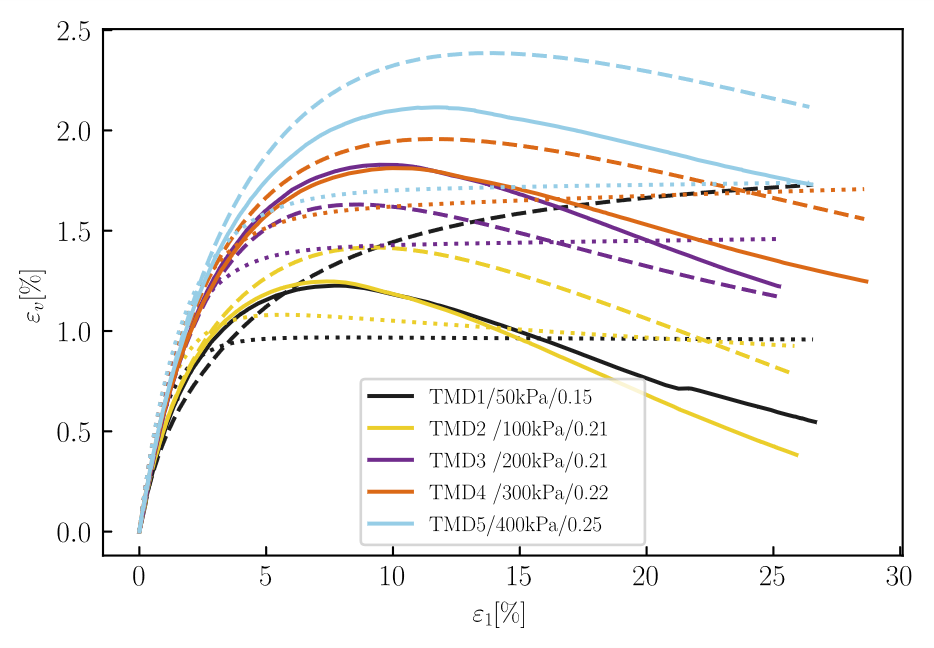

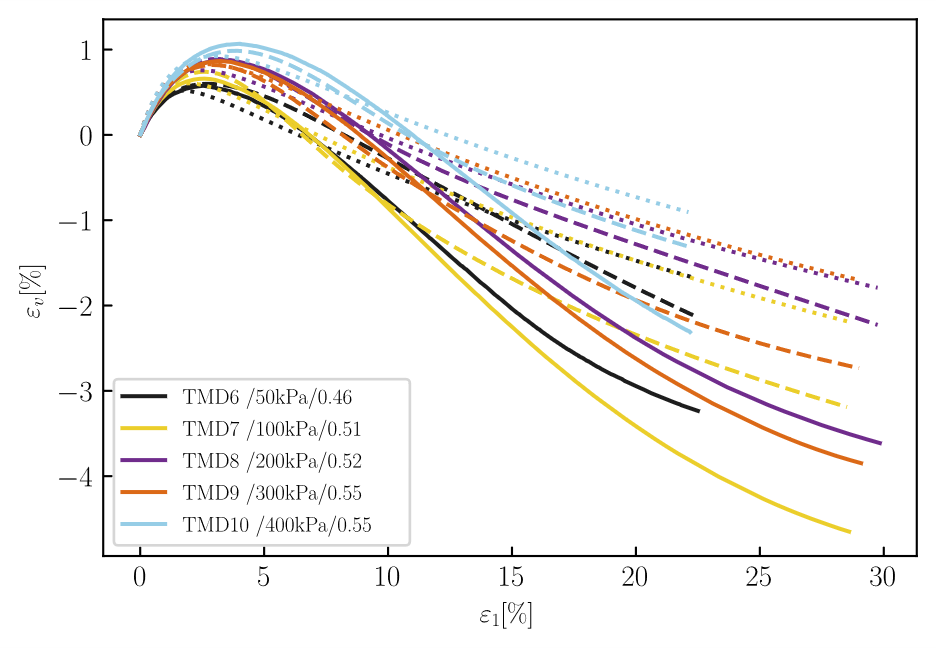

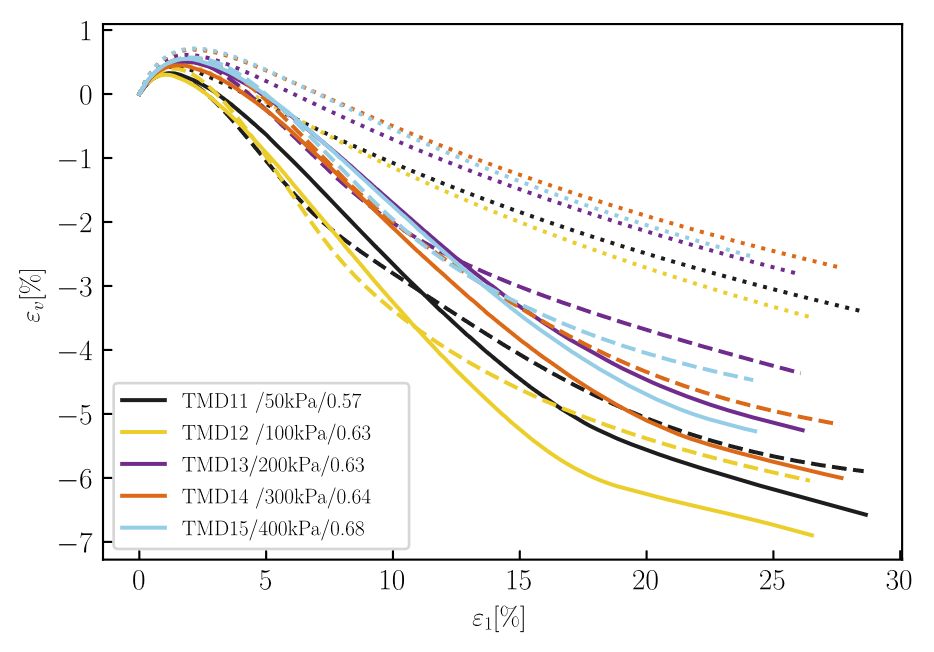


**(a)** **(b)** **(c)**


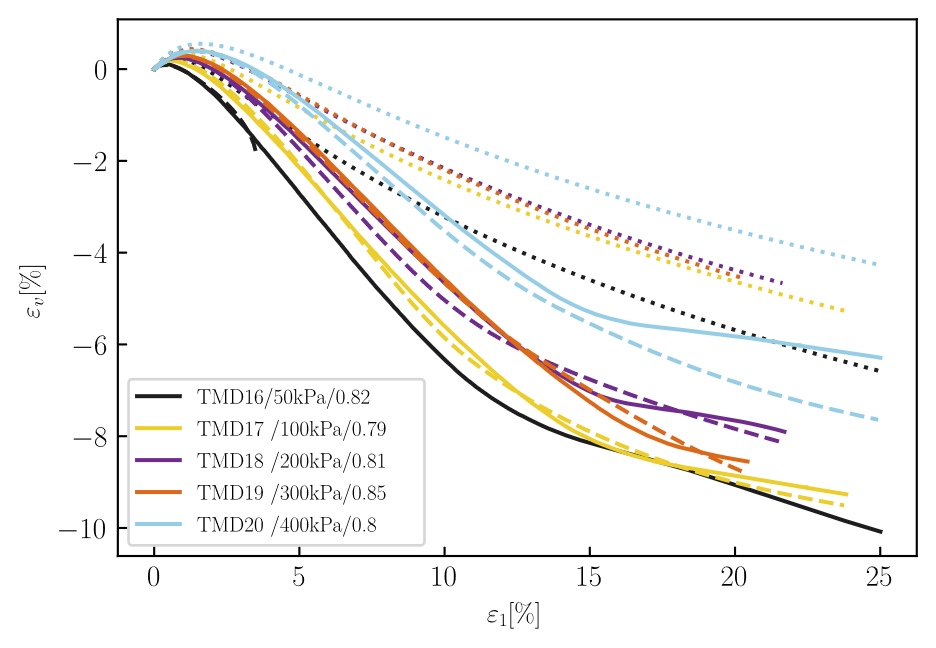

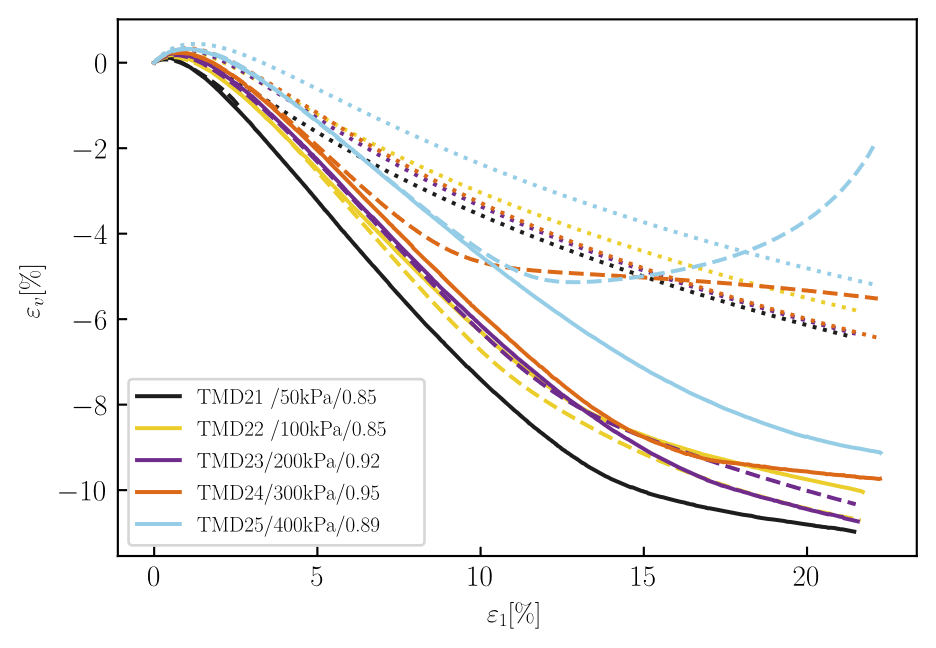


**(d)** **(e)**

**Fig. S6.** Volumetric strain $\varepsilon_{v}$ versus axial strain $\varepsilon_{1}$in drained simulations: (a) loose; (b) loose to medium dense; (c) medium dense; (d) medium dense to dense; (e) dense samples


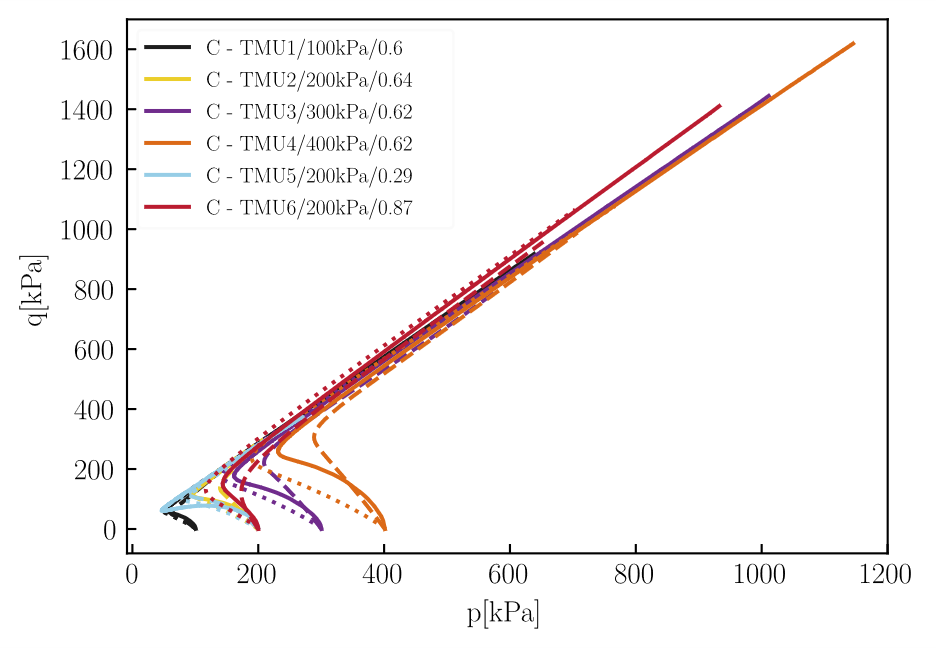

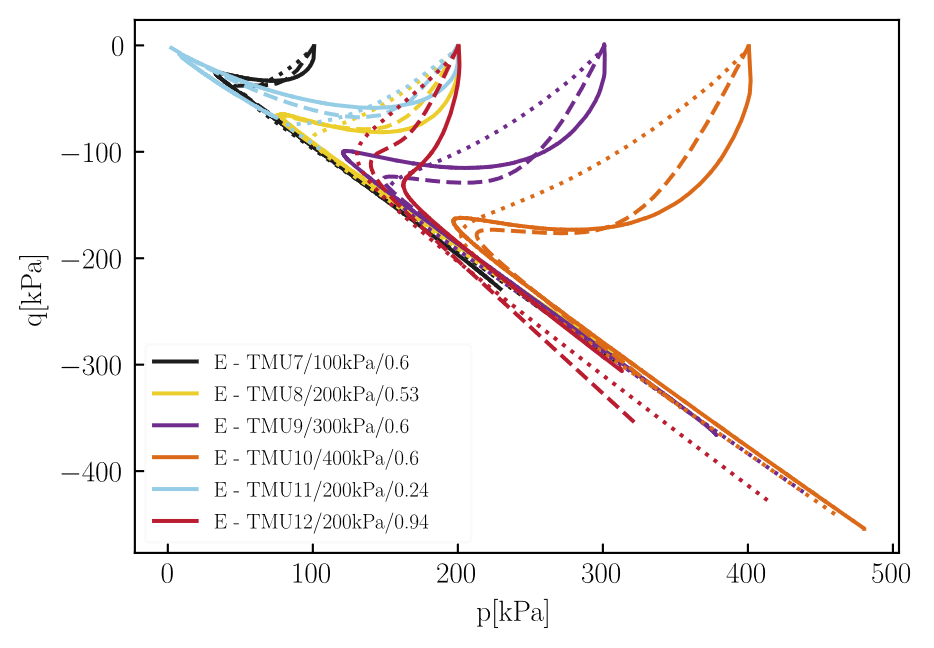


**(a)** **(b)**

**Fig. S7.** Effective stress paths in undrained simulations: (a) compression; (b) extension


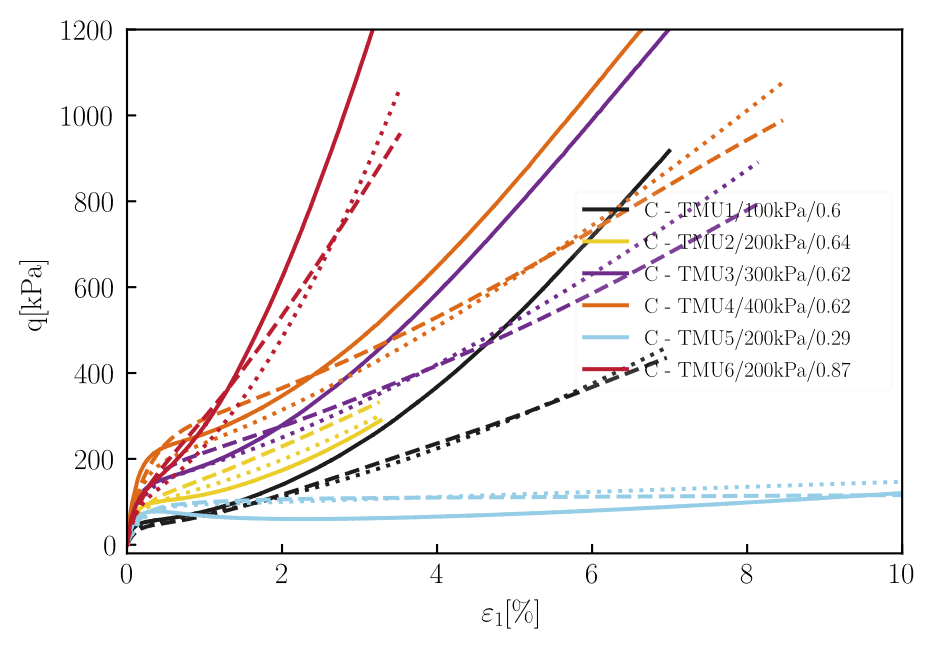

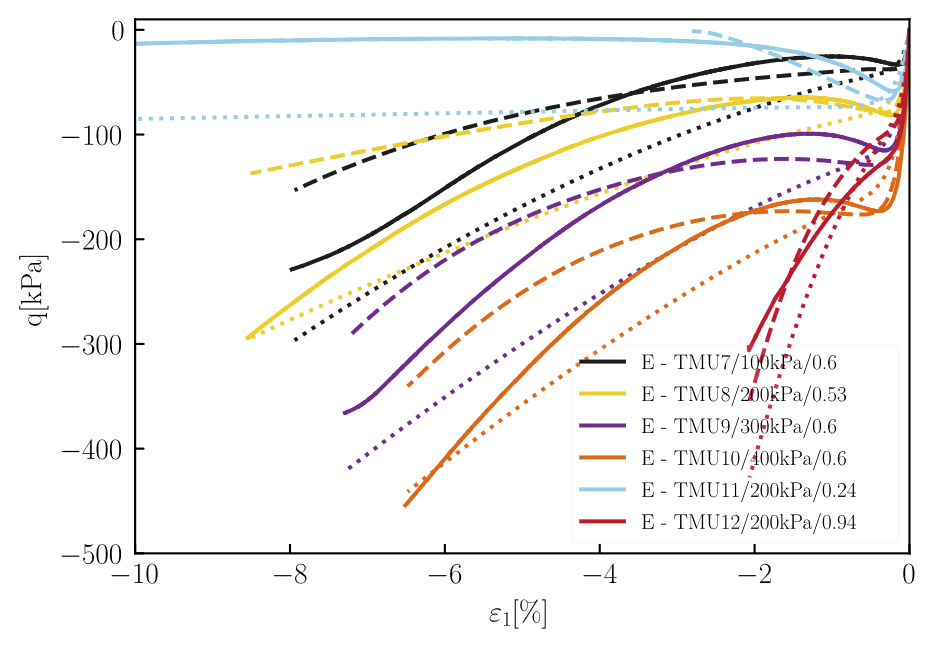


**(a)** **(b)**

**Fig. S8.** Stress-strain relationships in undrained simulations: (a) compression; (b) extension

### Results of Section 4.1.2.2


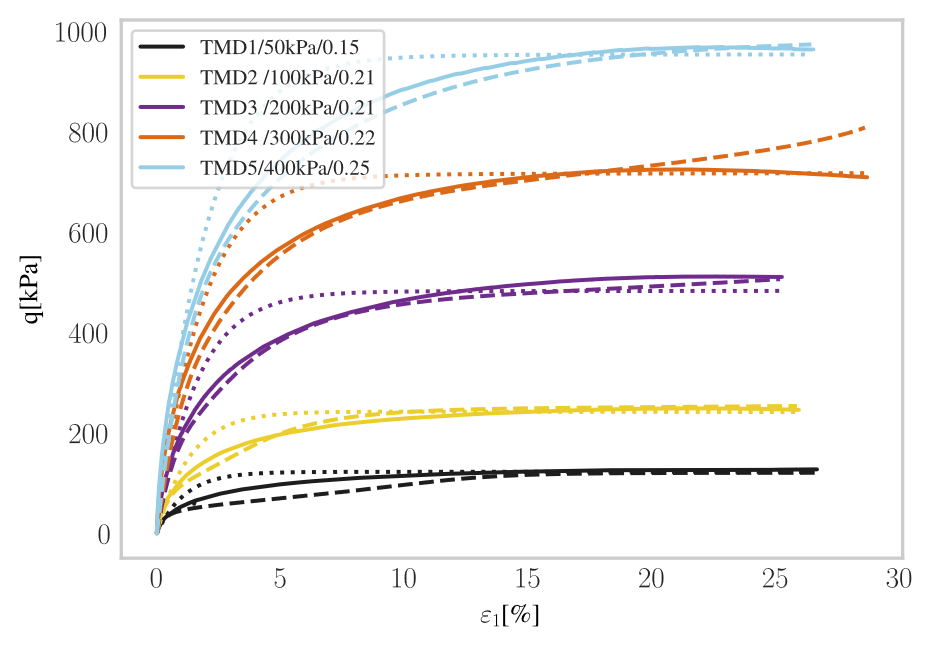

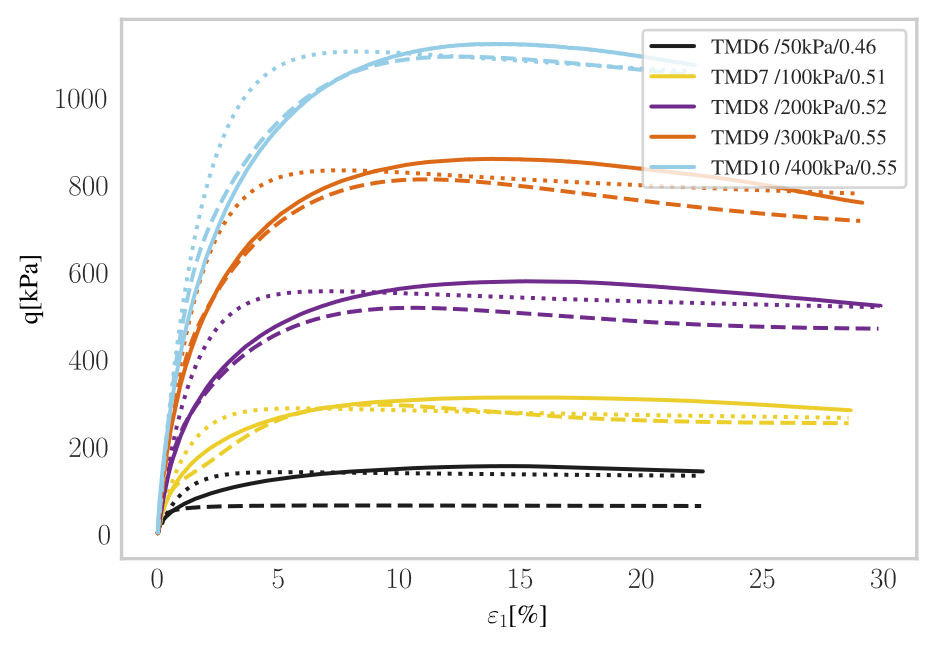

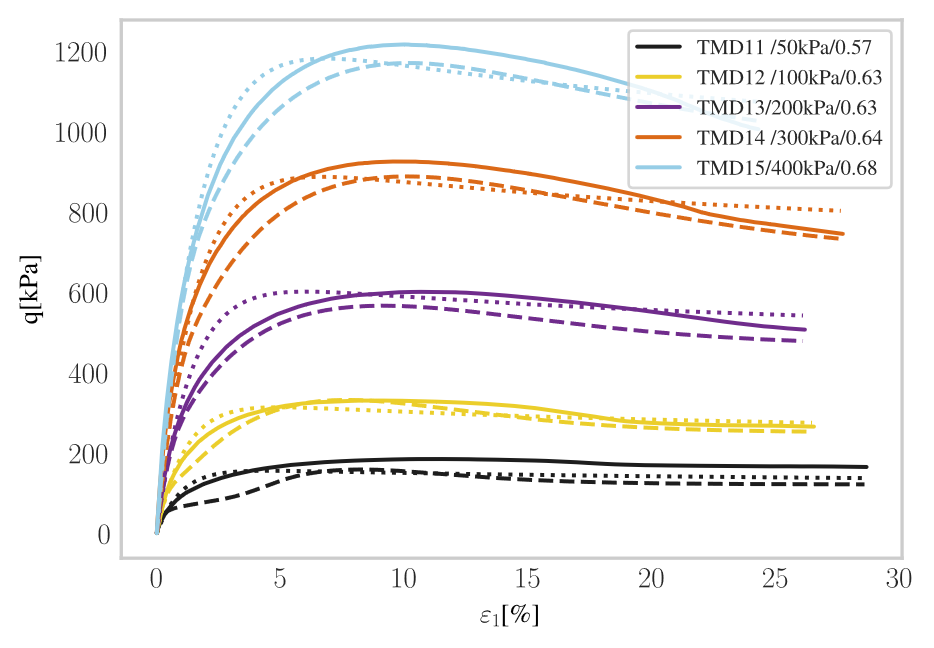
**(a)** **(b)** **(c)**


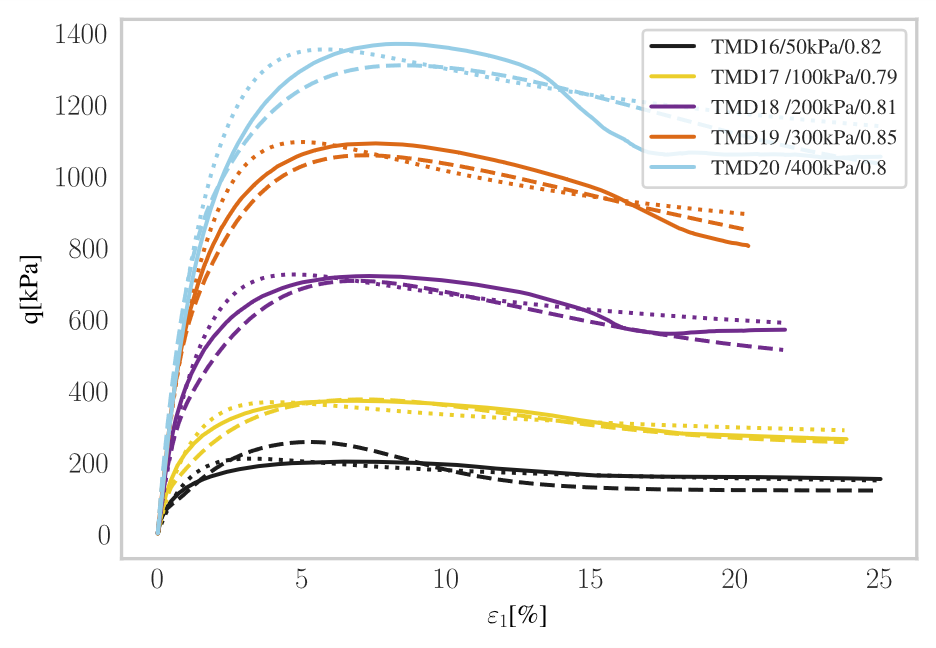

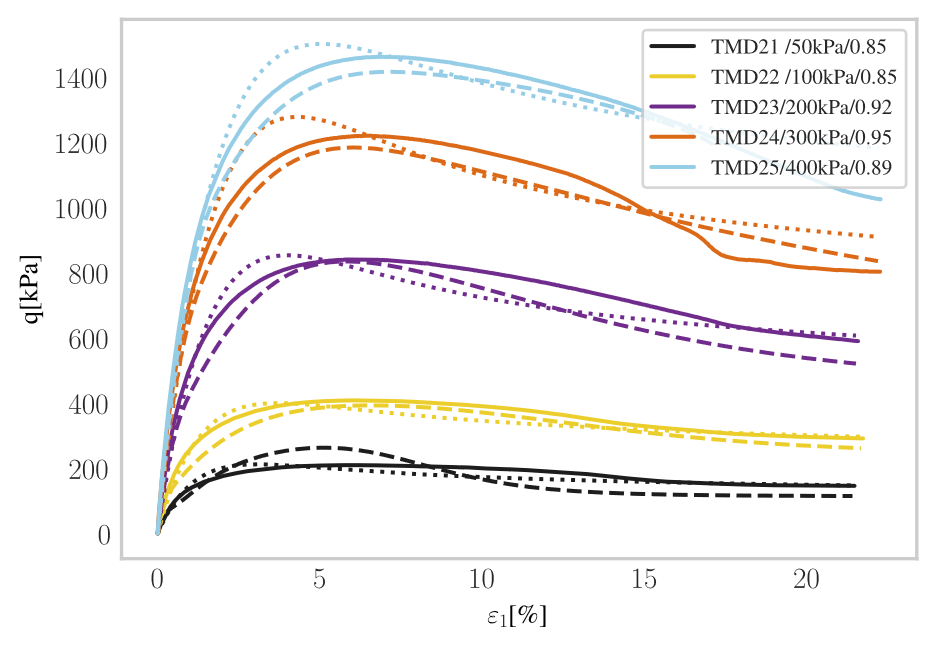


**(d)** **(e)**

**Fig. S9.** Deviatoric stress responses in drained simulations: (a) loose; (b) loose to medium dense; (c) medium dense; (d) medium dense to dense; (e) dense samples


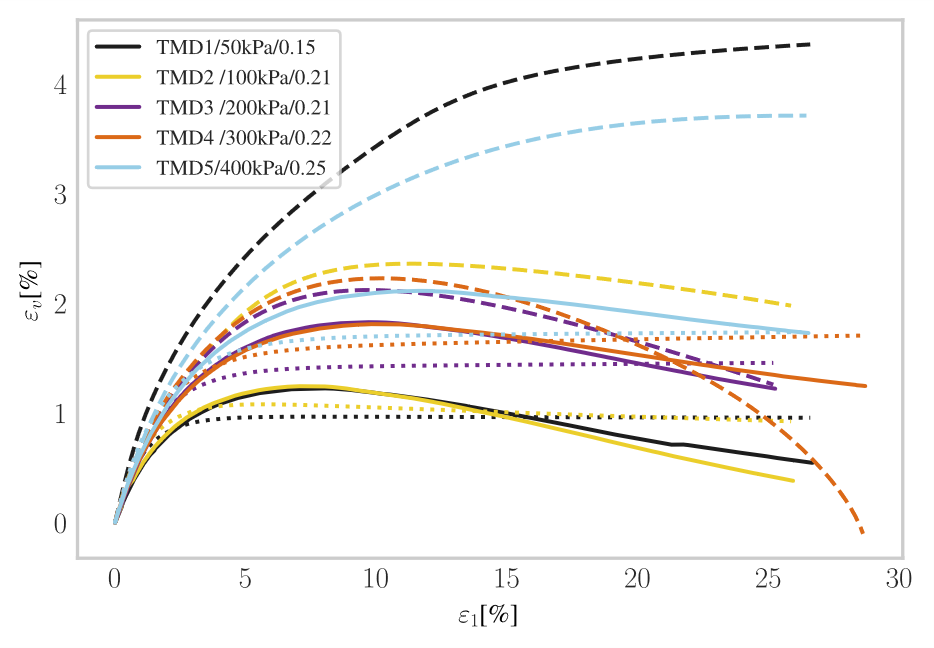

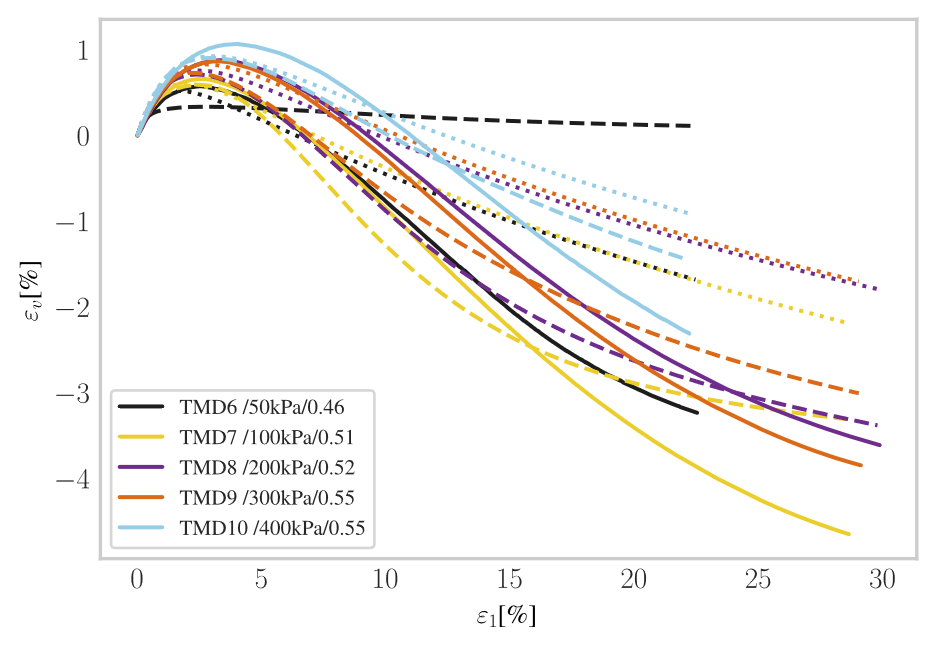

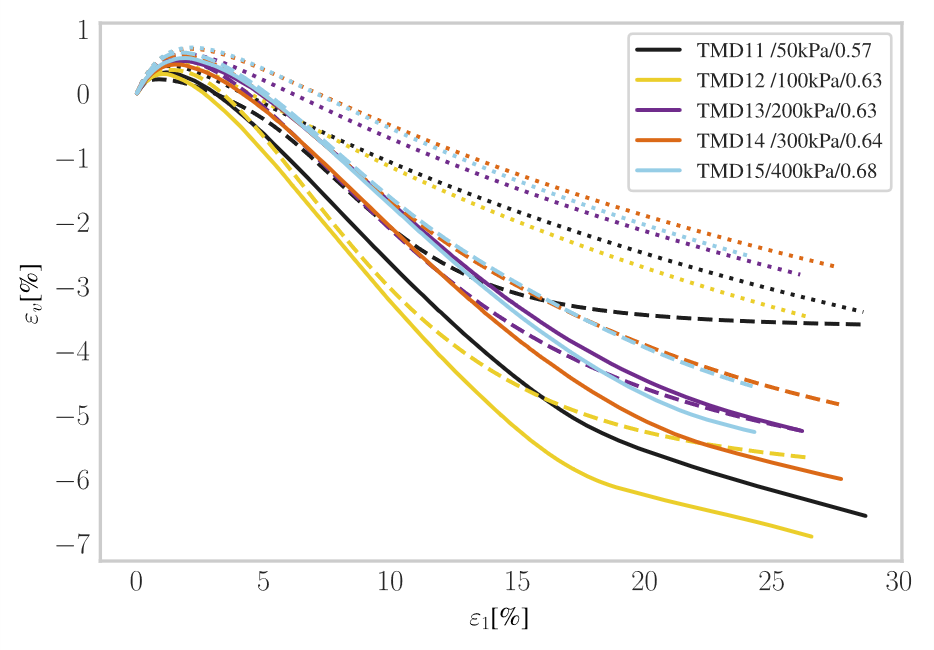
**(a)** **(b)** **(c)**


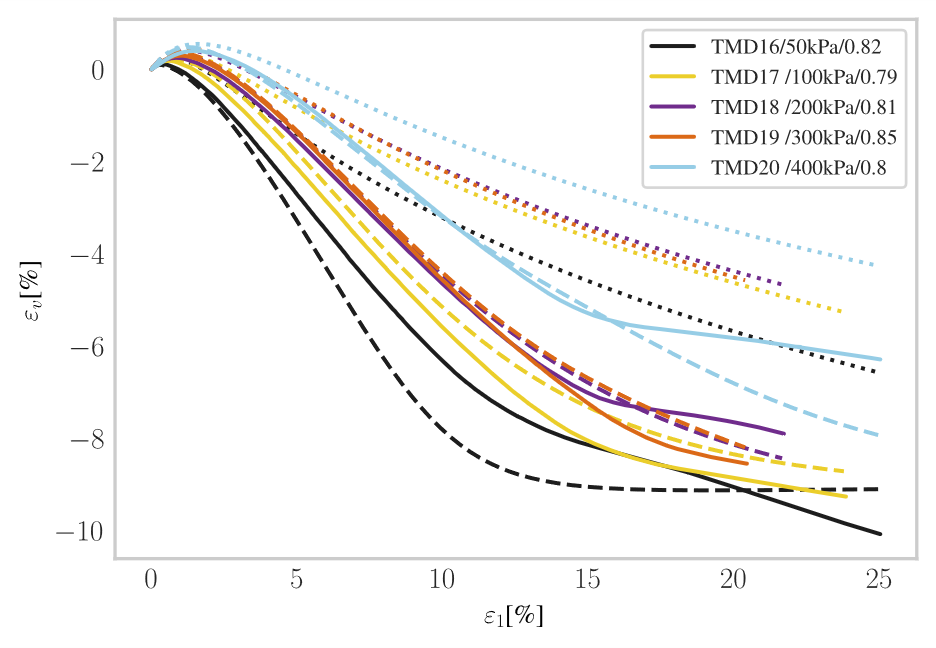

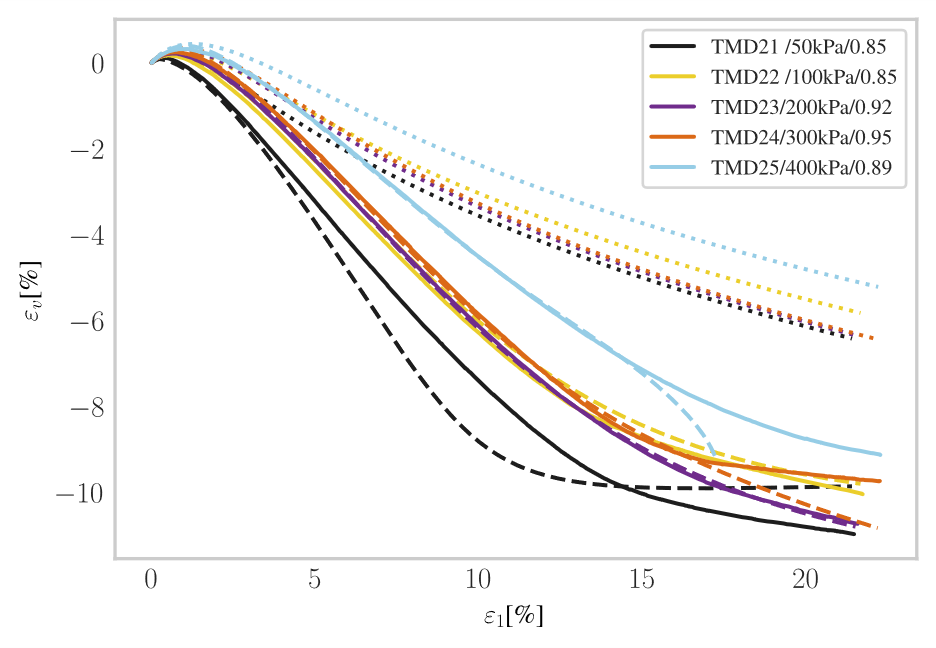


**(d)** **(e)**

**Fig. S10.** Volumetric strain $\varepsilon_{v}$ versus axial strain $\varepsilon_{1}$in drained simulations: (a) loose; (b) loose to medium dense; (c) medium dense; (d) medium dense to dense; (e) dense samples


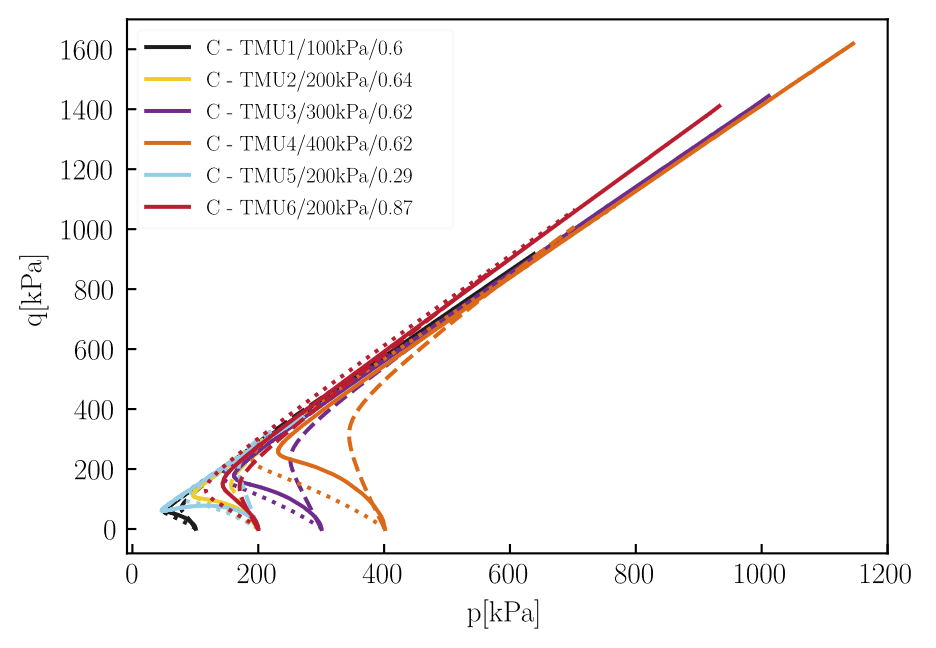

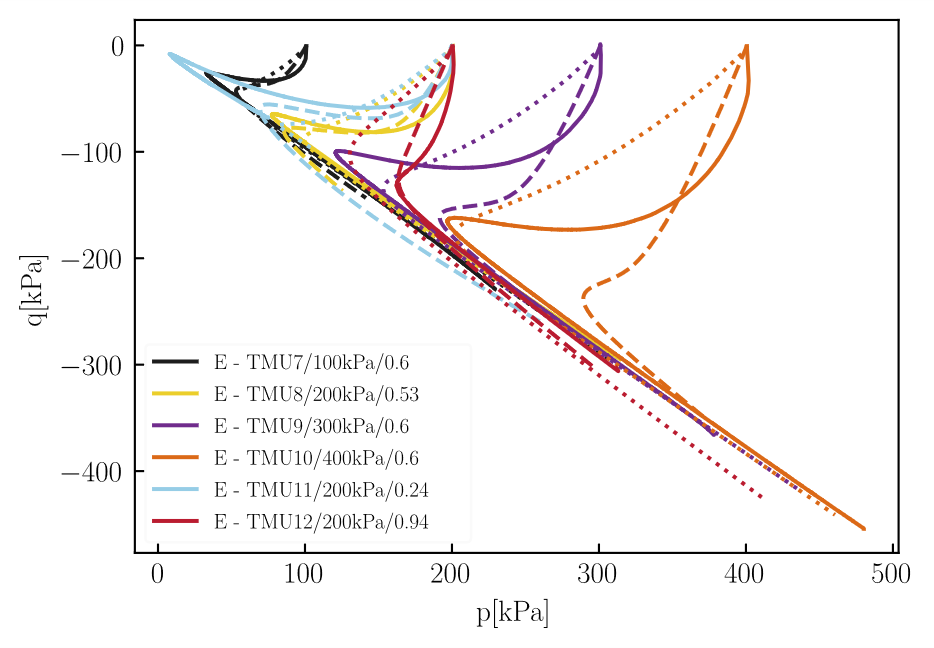


**(a)** **(b)**

**Fig. S11.** Effective stress paths in undrained simulations: (a) compression; (b) extension


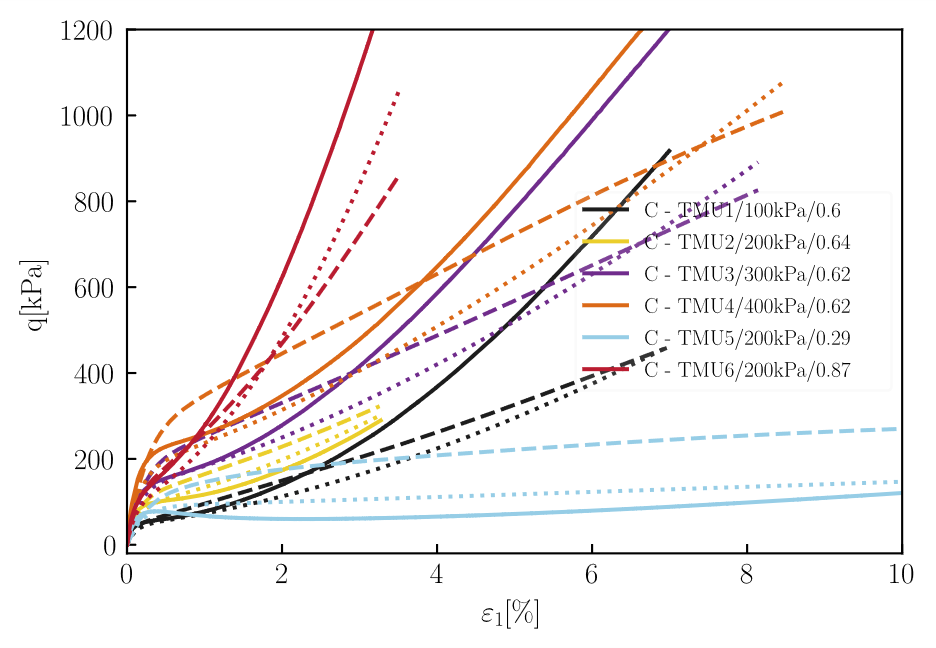

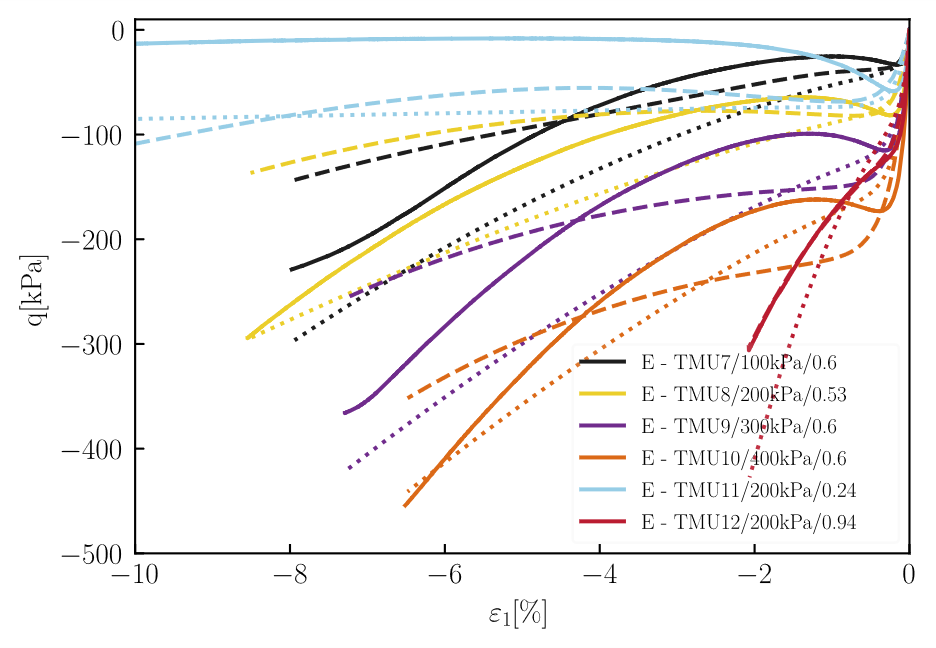


**(a)** **(b)**

**Fig. S12.** Stress-strain relationships in undrained simulations: (a) compression; (b) extension

### Results of Section 4.1.2.3


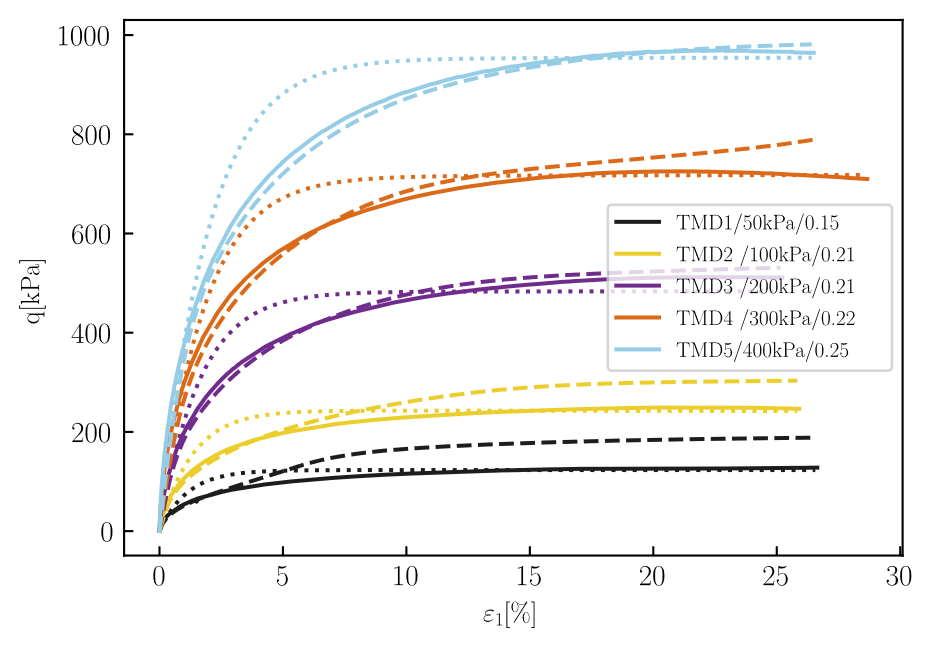

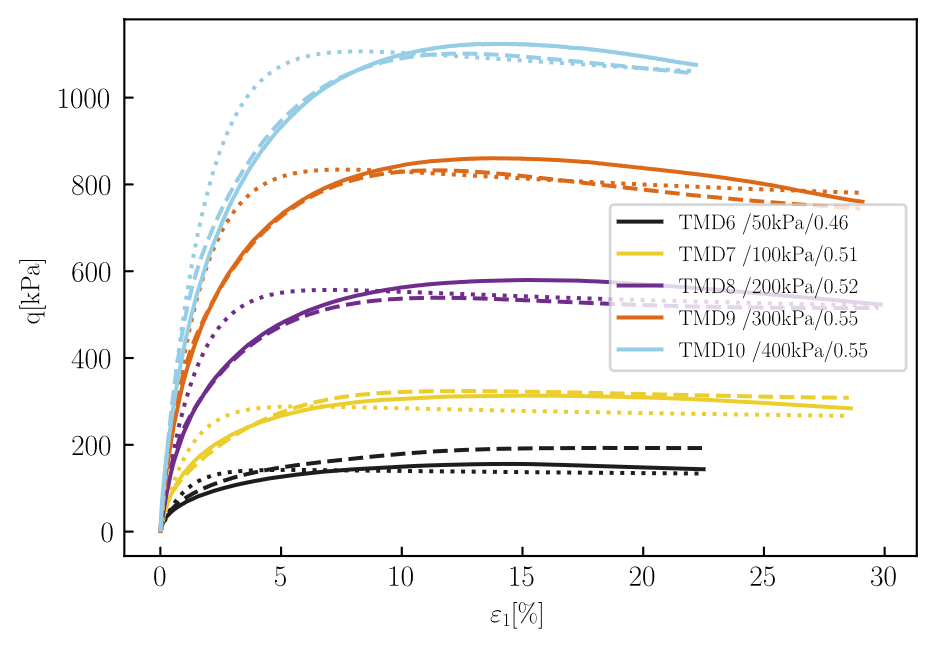

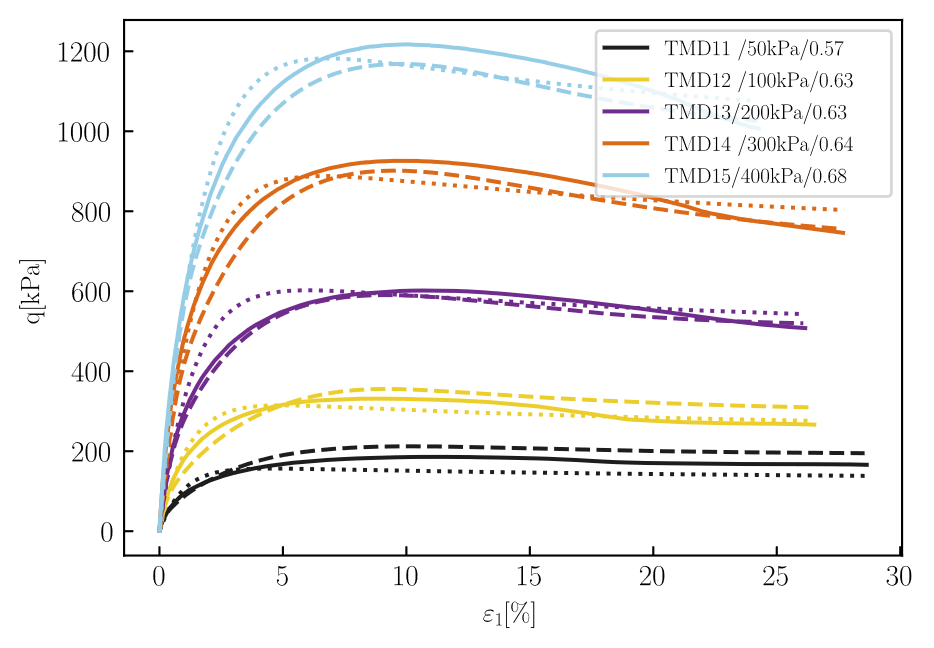


**(a)** **(b)** **(c)**


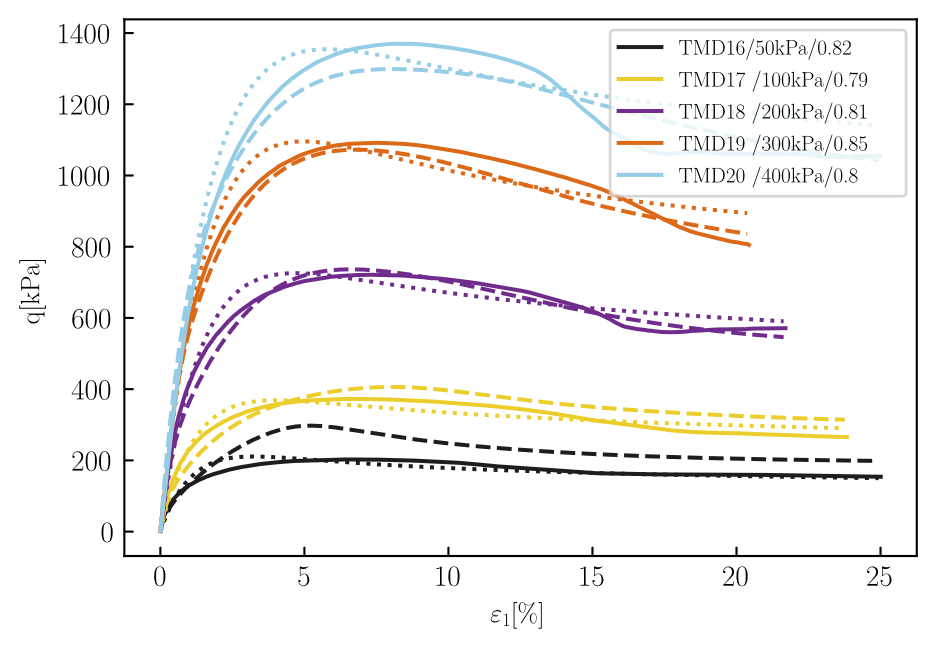

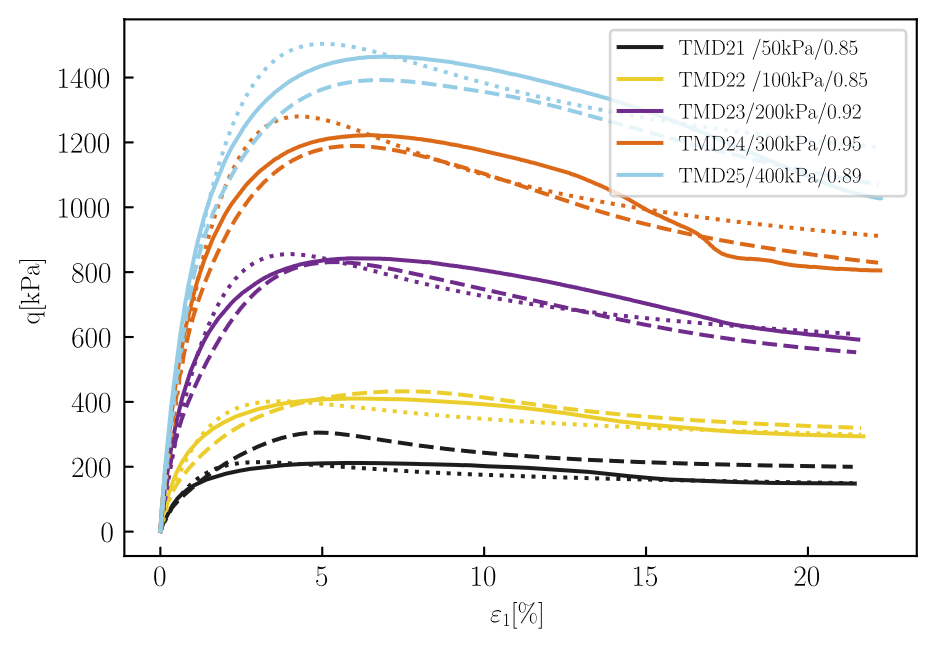


**(d)** **(e)**

**Fig. S13.** Deviatoric stress responses in drained simulations: (a) loose; (b) loose to medium dense; (c) medium dense; (d) medium dense to dense; (e) dense samples


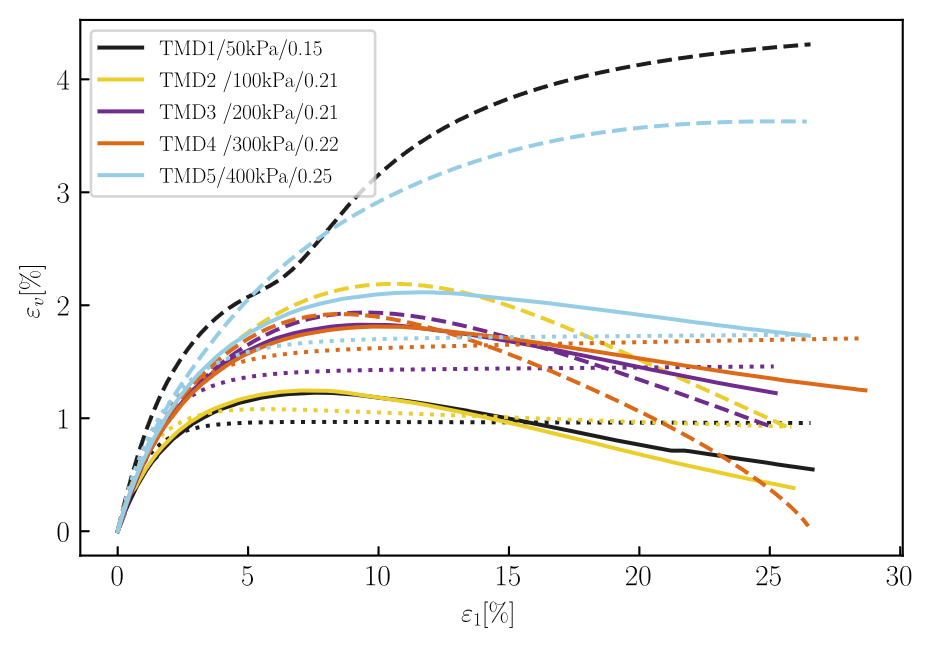

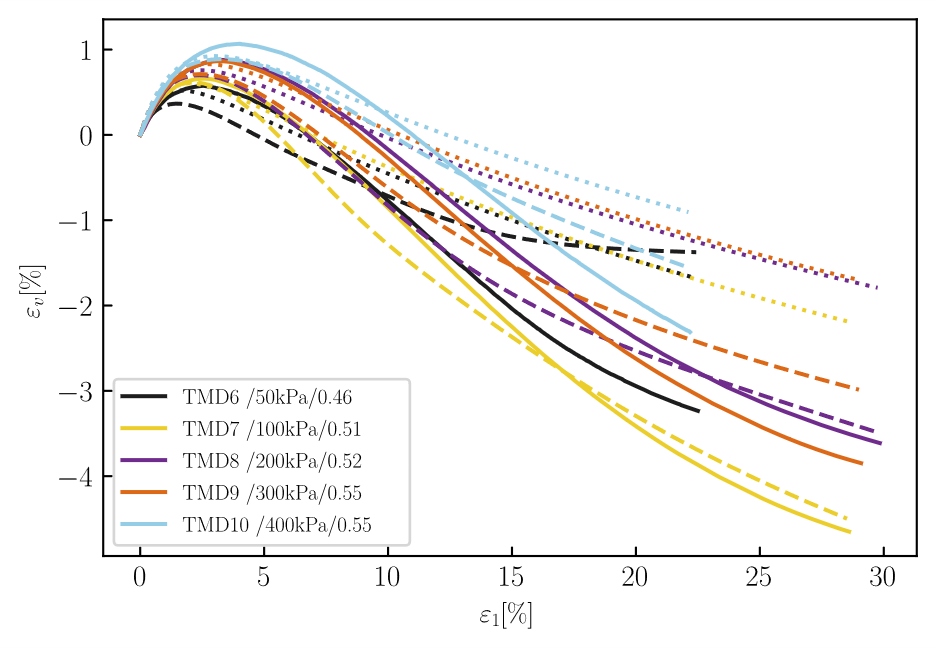

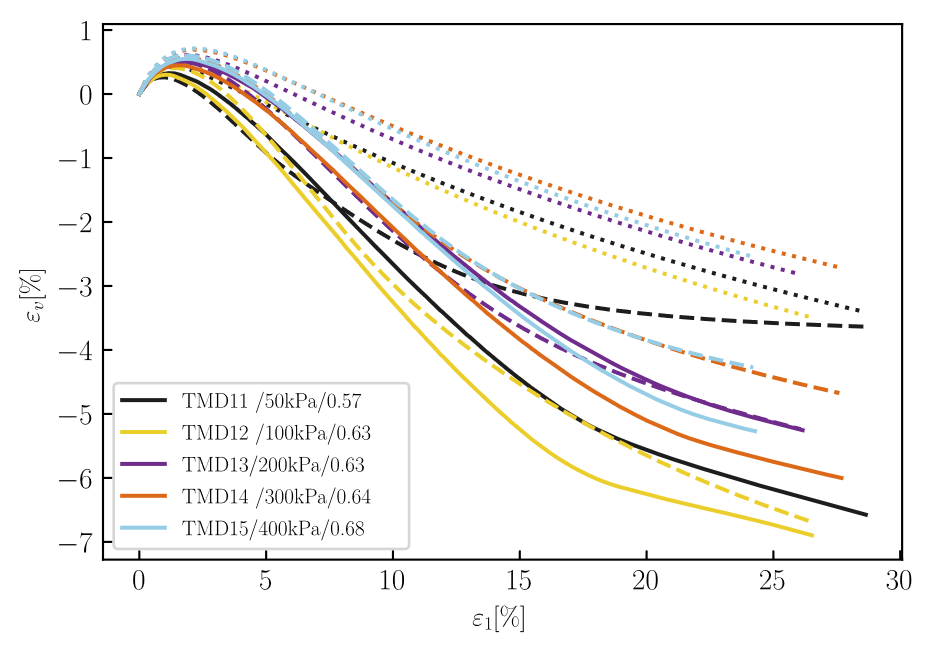


**(a)** **(b)** **(c)**


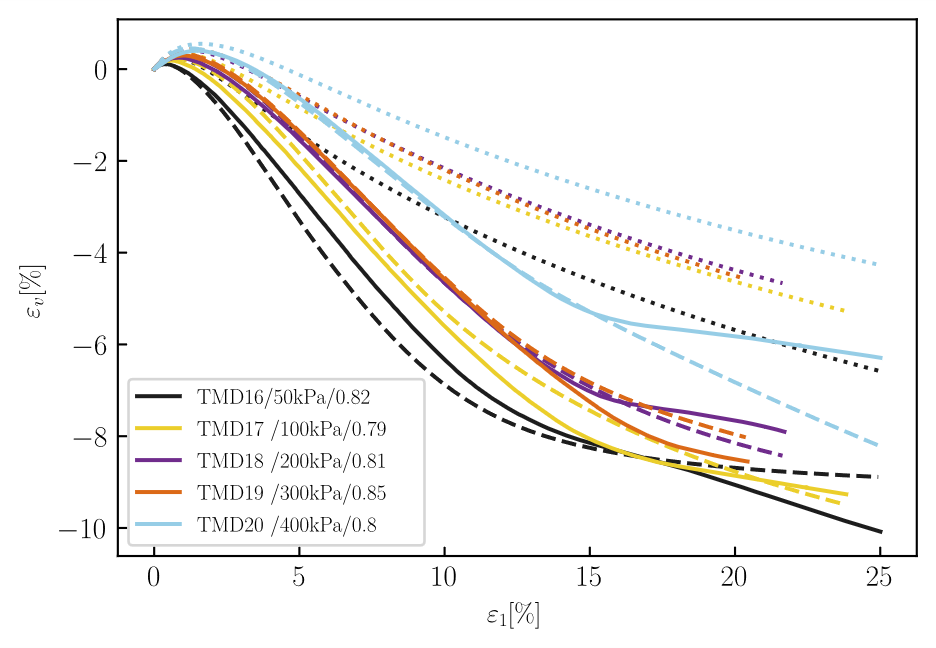

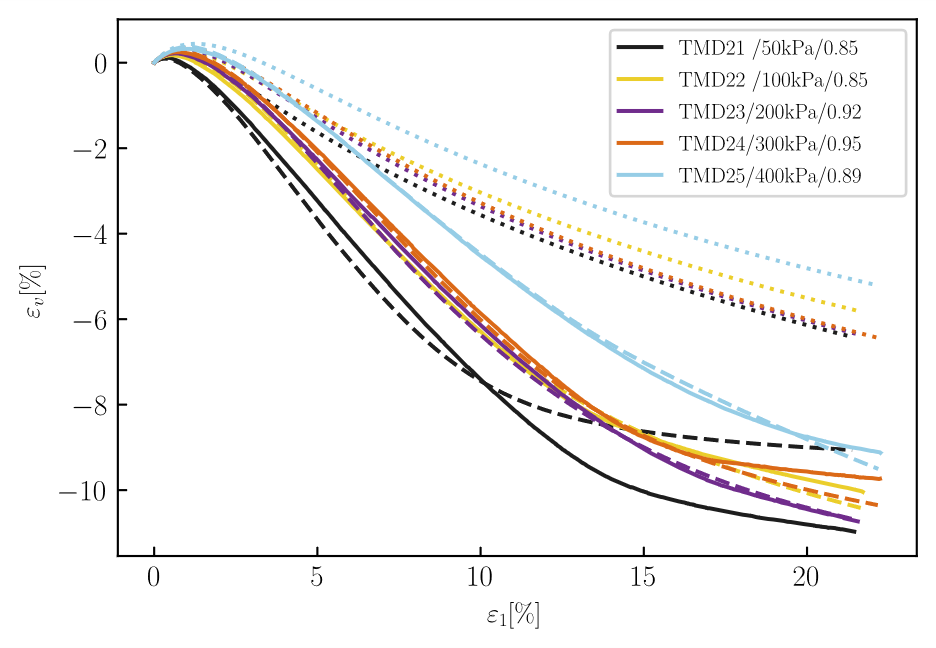


**(d)** **(e)**

**Fig. S14.** Volumetric strain $\varepsilon_{v}$ versus axial strain $\varepsilon_{1}$in drained simulations: (a) loose; (b) loose to medium dense; (c) medium dense; (d) medium dense to dense; (e) dense samples


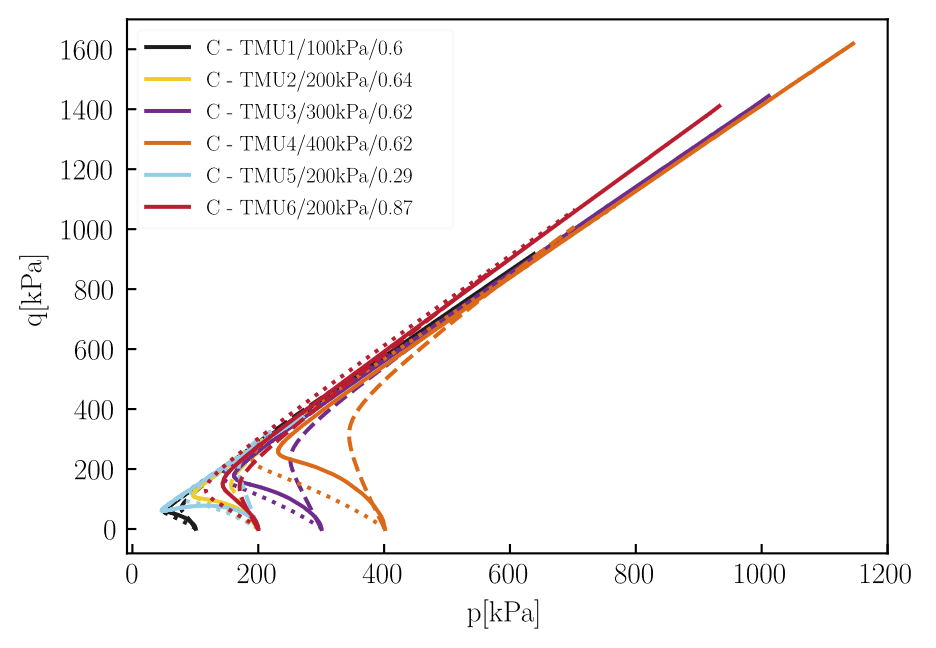

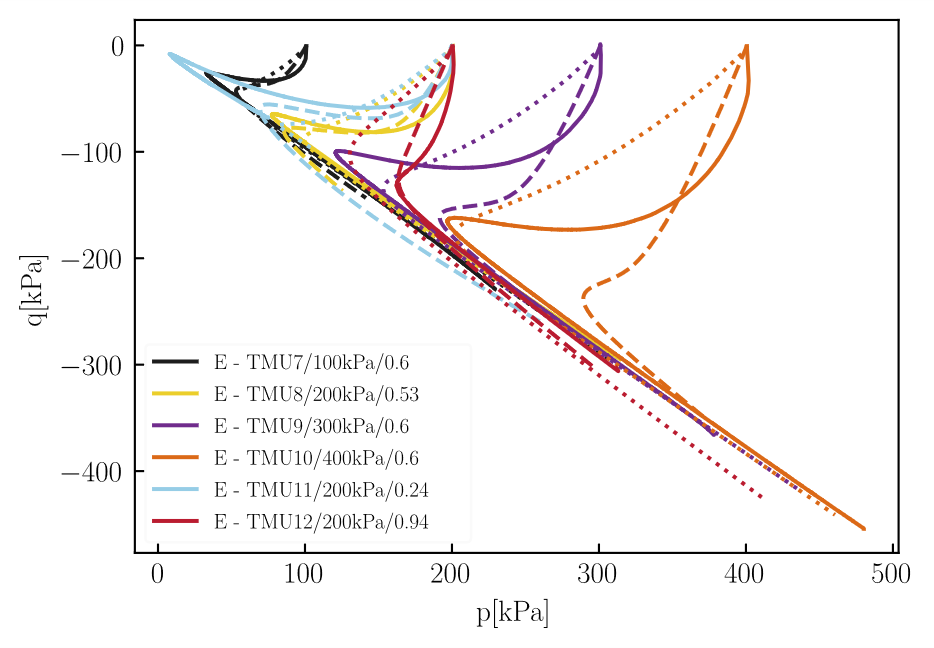


**(a)** **(b)**

**Fig. S15.** Effective stress paths in undrained simulations: (a) compression; (b) extension


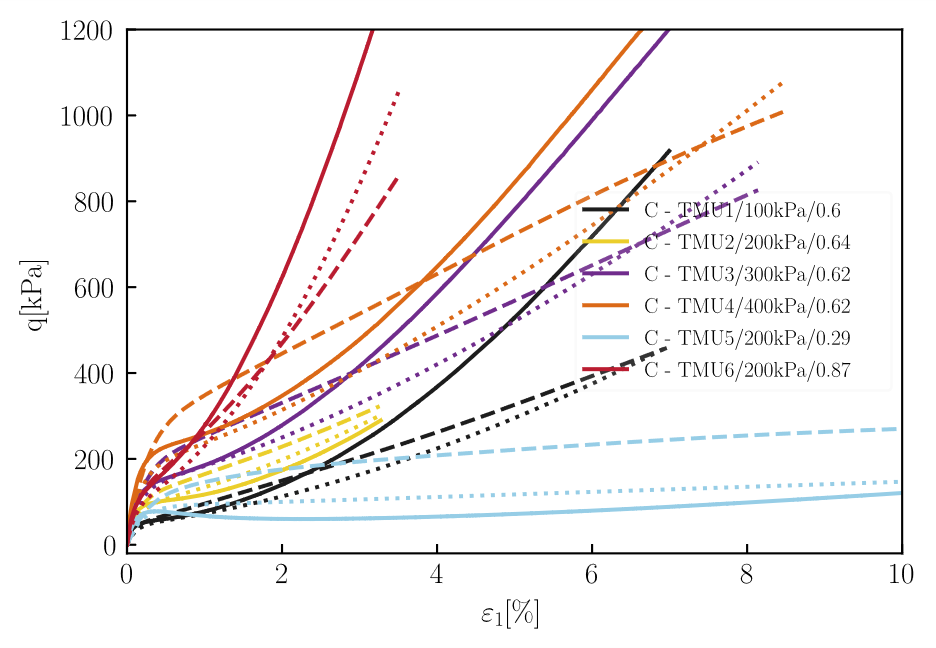

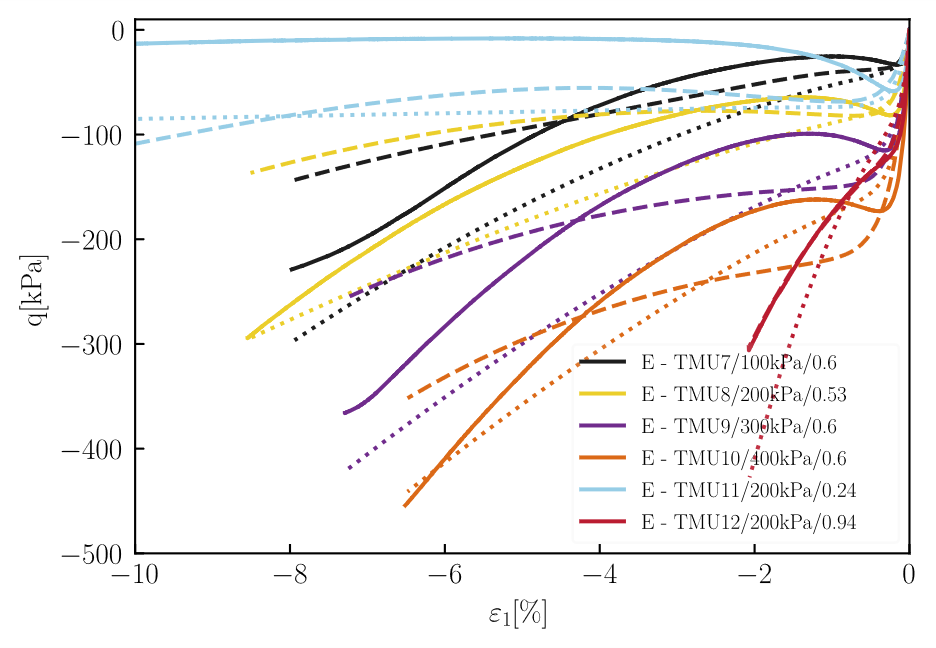


**(a)** **(b)**

**Fig. S16.** Stress-strain relationships in undrained simulations: (a) compression; (b) extension

### Results of Section 4.1.2.4


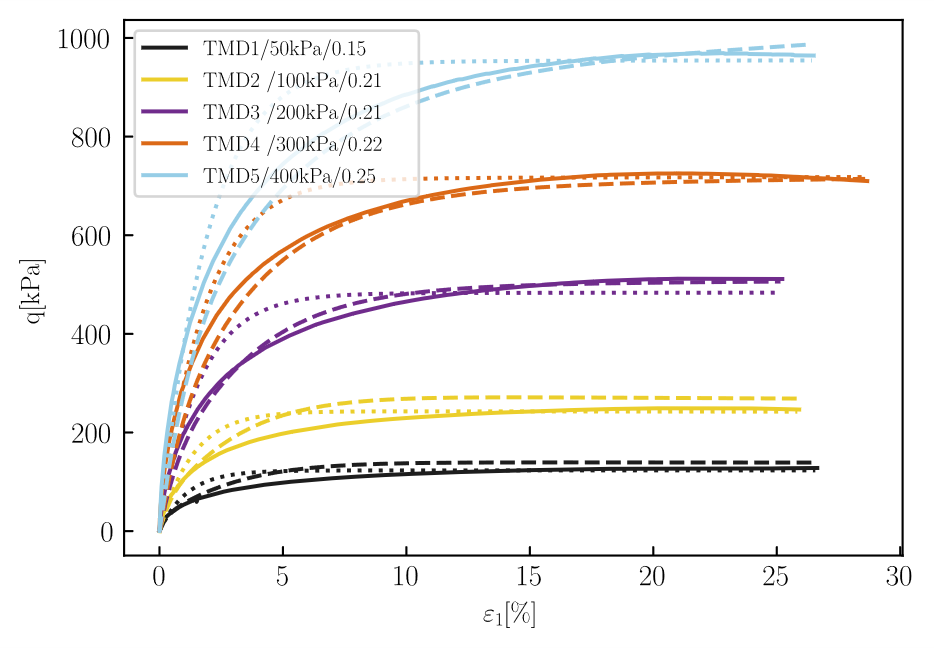

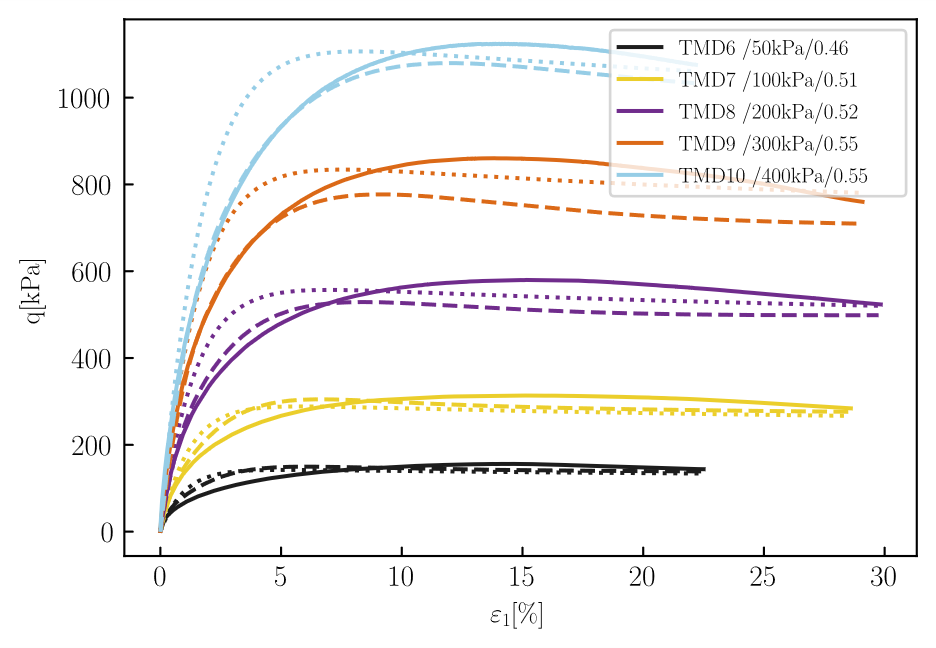

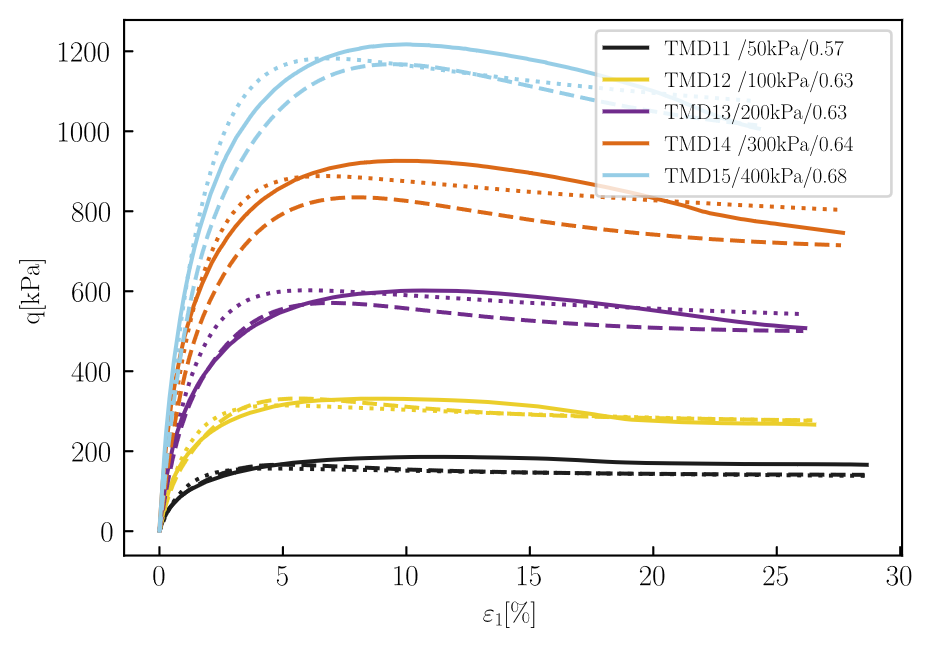


**(a)** **(b)** **(c)**


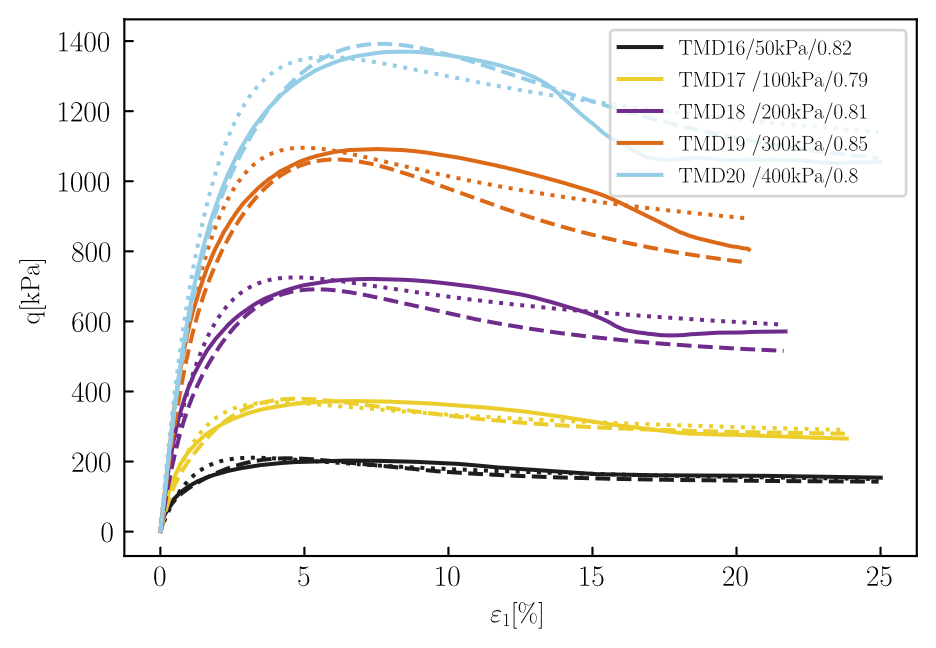

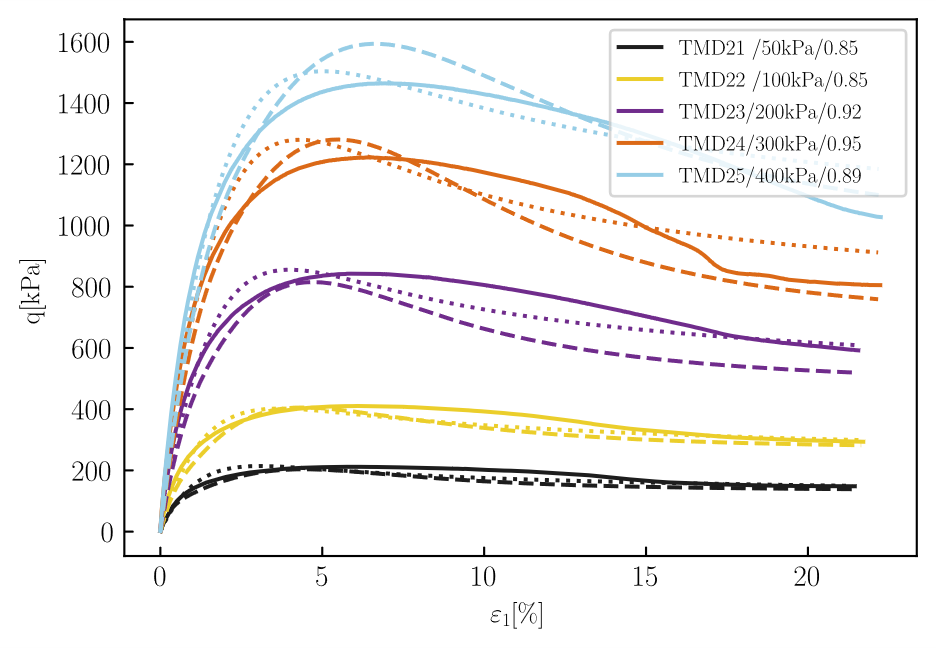


**(d)** **(e)**

**Fig. S17.** Deviatoric stress responses in drained simulations: (a) loose; (b) loose to medium dense; (c) medium dense; (d) medium dense to dense; (e) dense samples


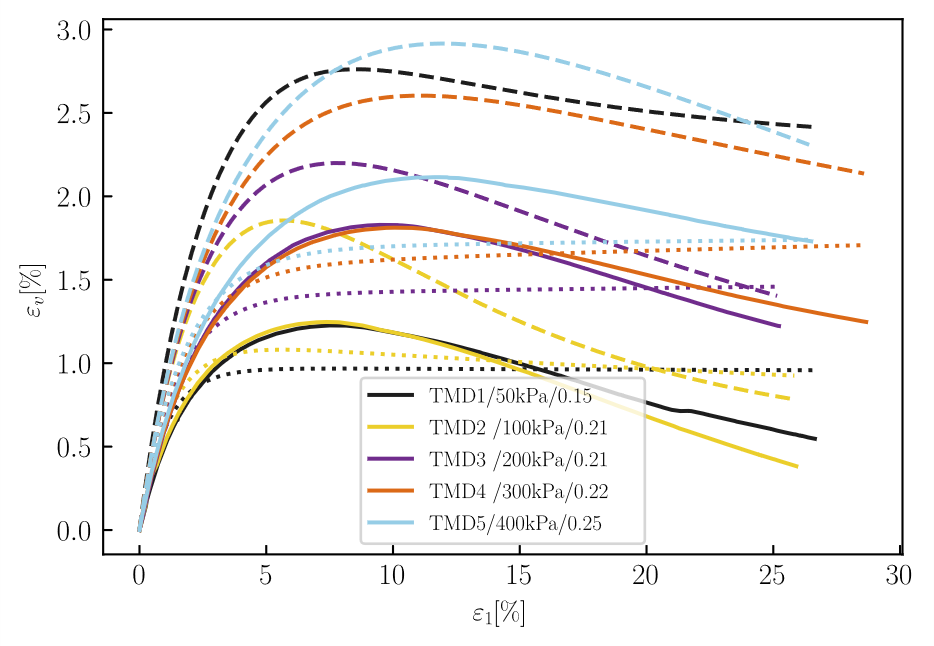

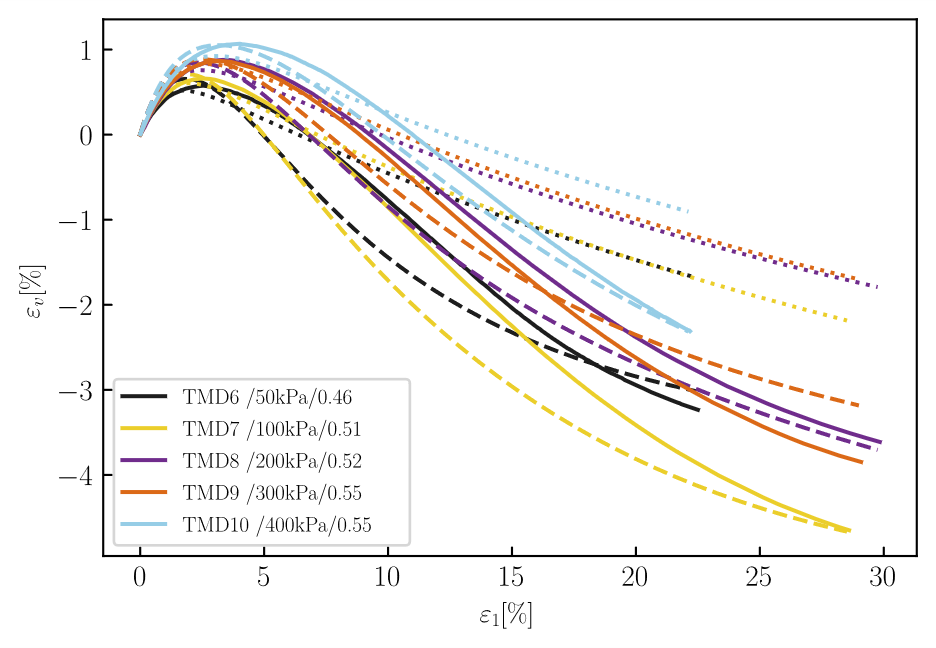

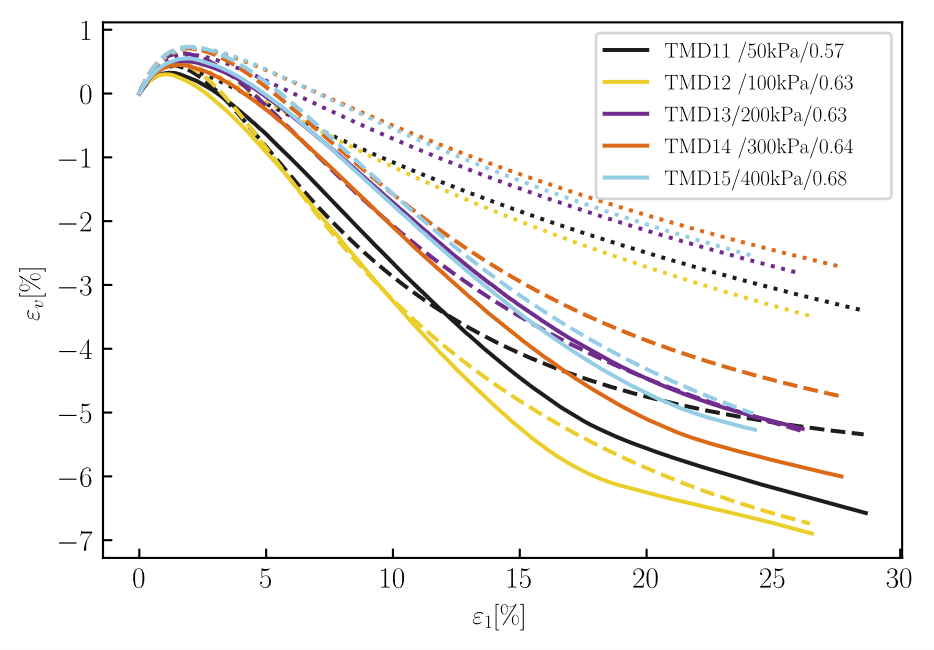


**(a)** **(b)** **(c)**


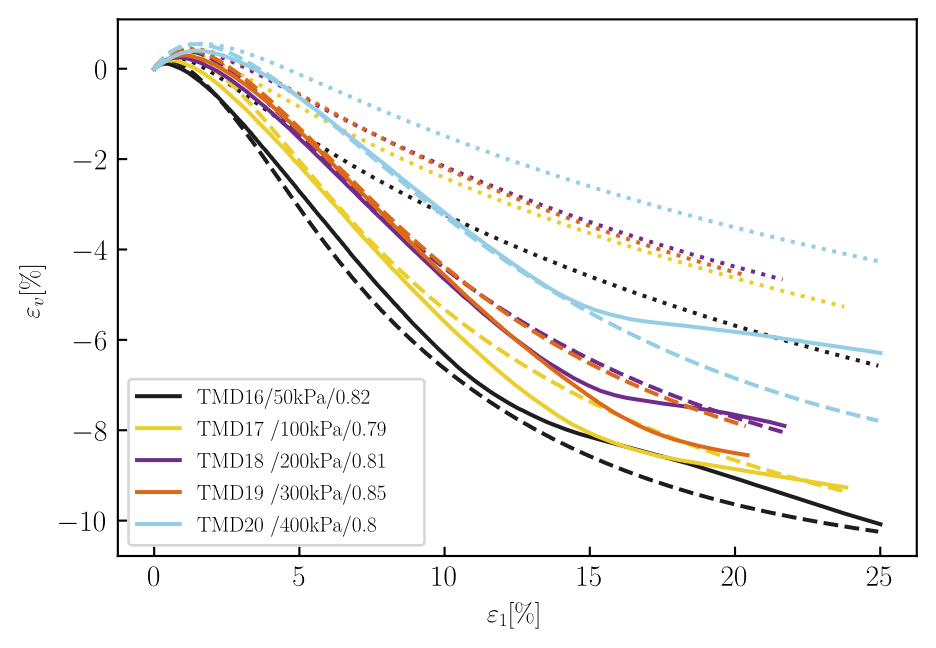

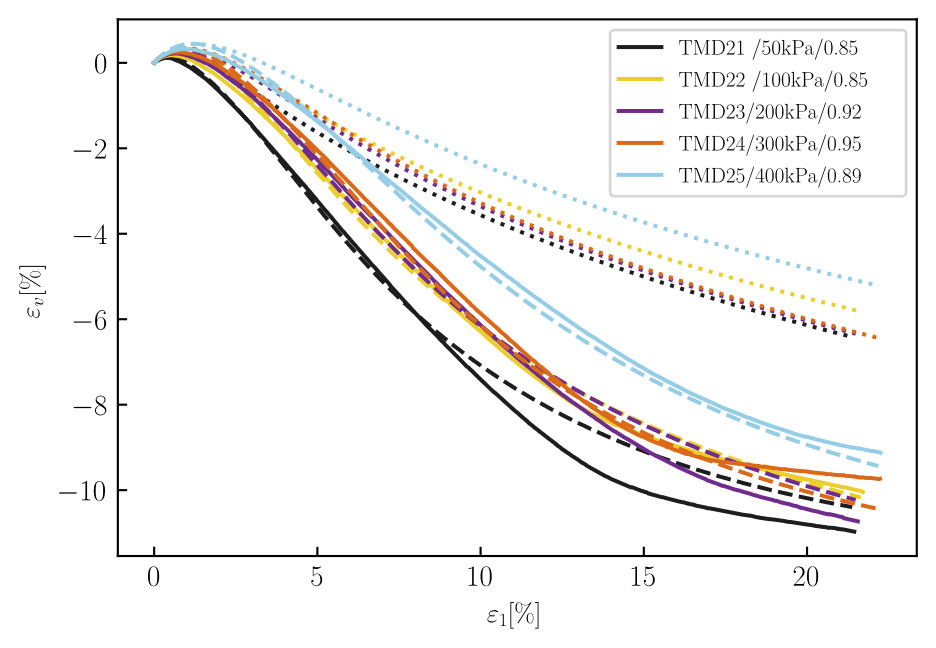


**(d)** **(e)**

**Fig. S18.** Volumetric strain $\varepsilon_{v}$ versus axial strain $\varepsilon_{1}$in drained simulations: (a) loose; (b) loose to medium dense; (c) medium dense; (d) medium dense to dense; (e) dense samples


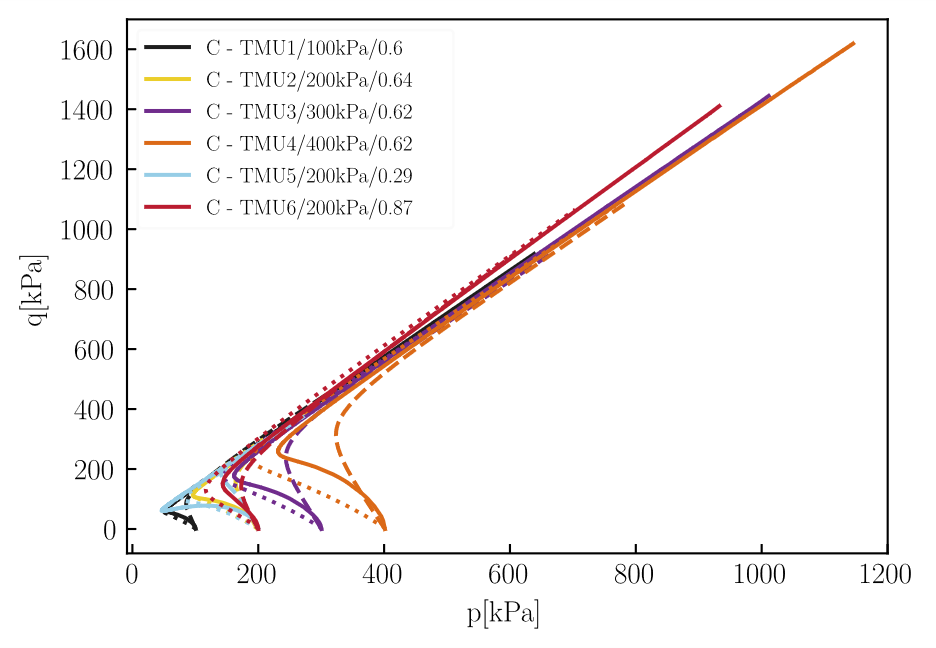

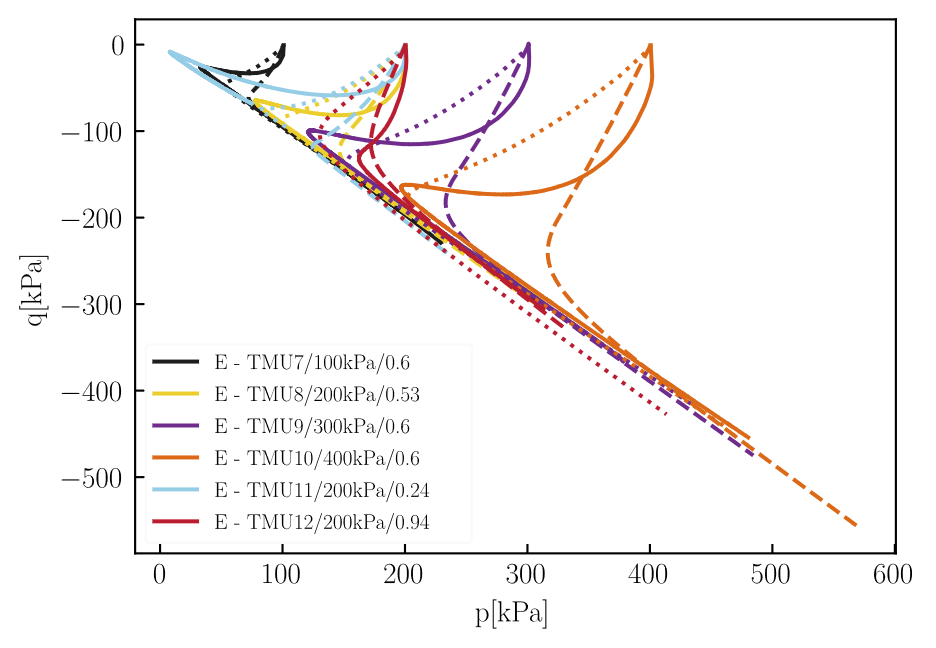


**(a)** **(b)**

**Fig. S19.** Effective stress paths in undrained simulations: (a) compression; (b) extension


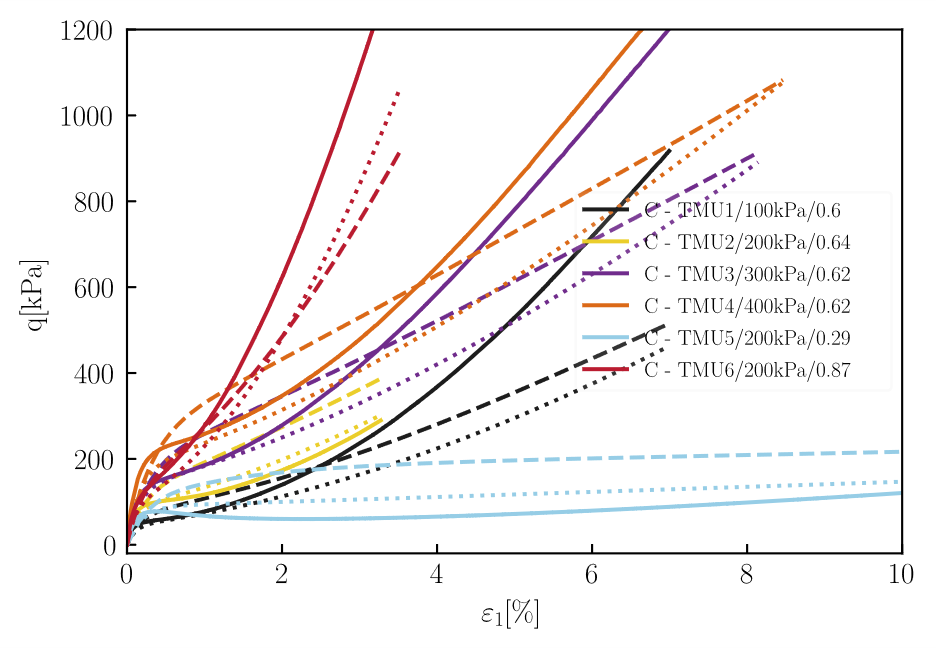

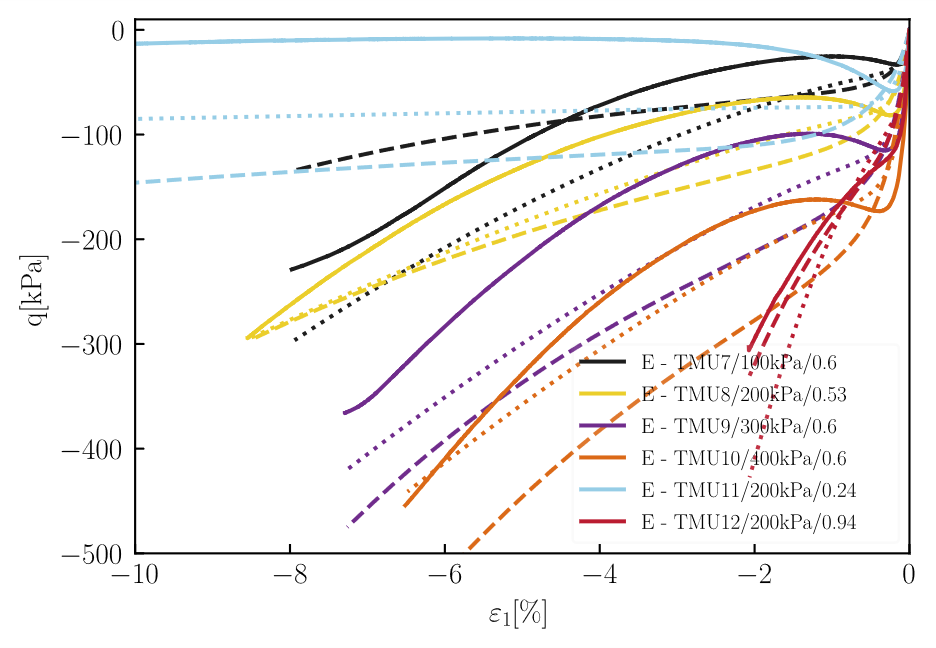


**(a)** **(b)**

**Fig. S20.** Stress-strain relationships in undrained simulations: (a) compression; (b) extension

### Results of Section 4.2.1


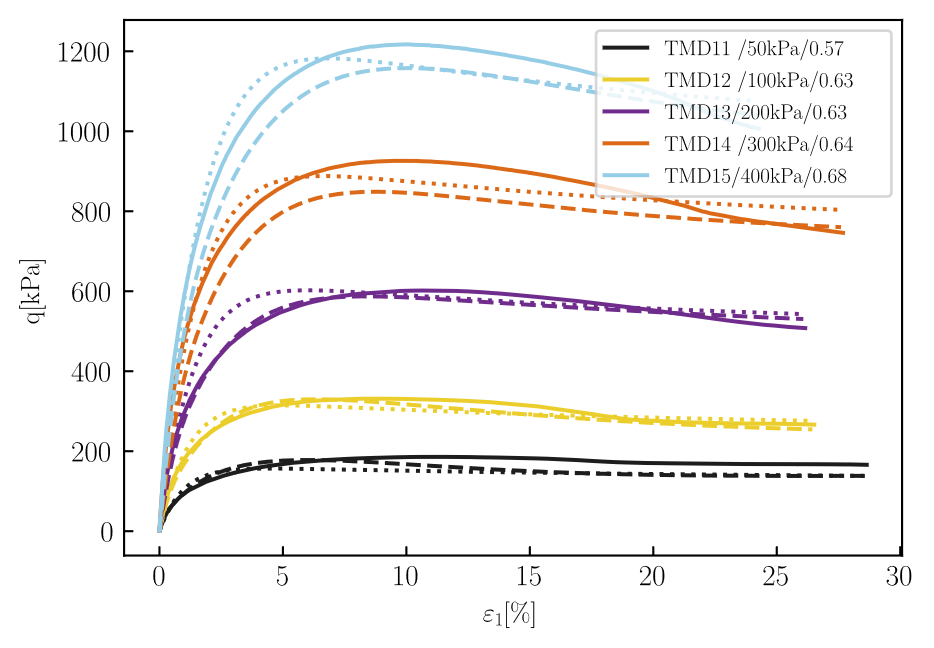

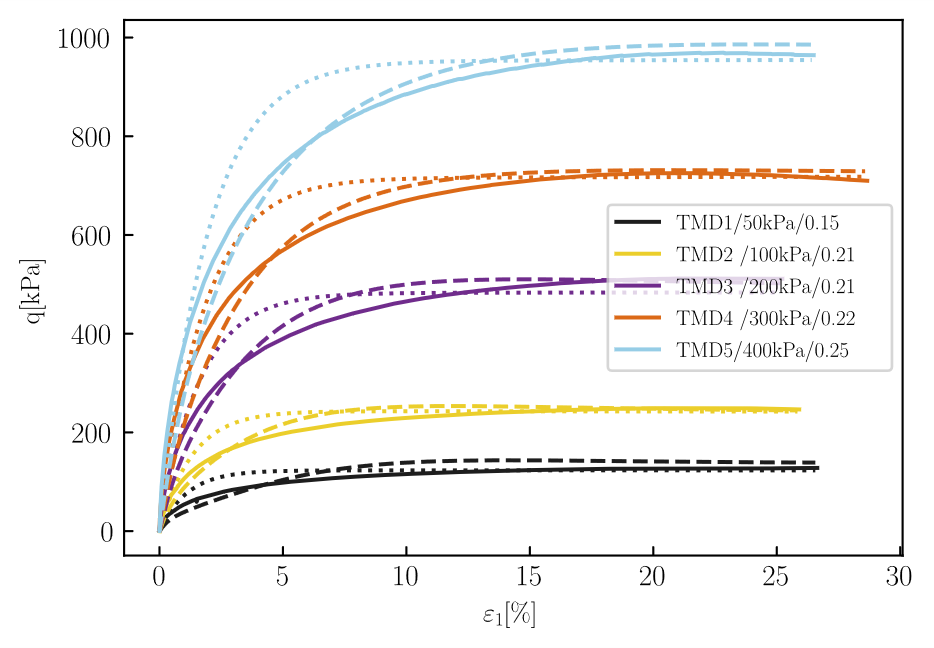

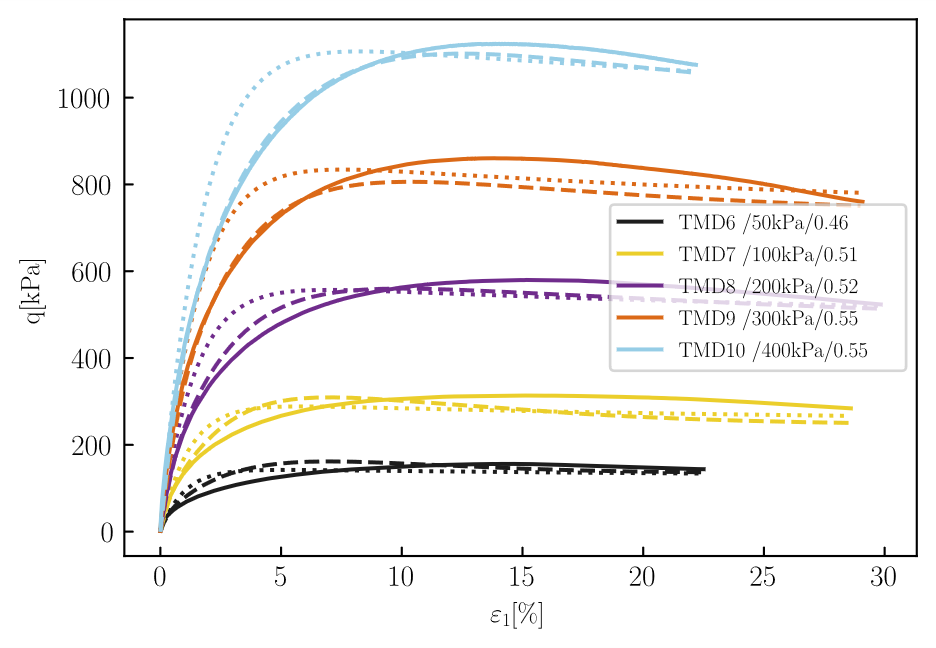


**(a)** **(b)** **(c)**


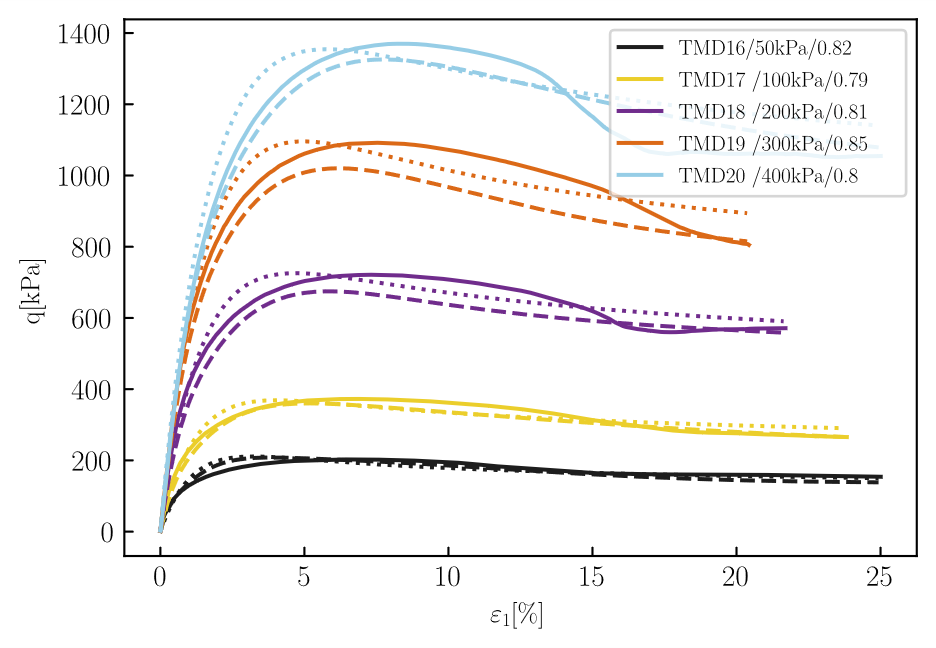

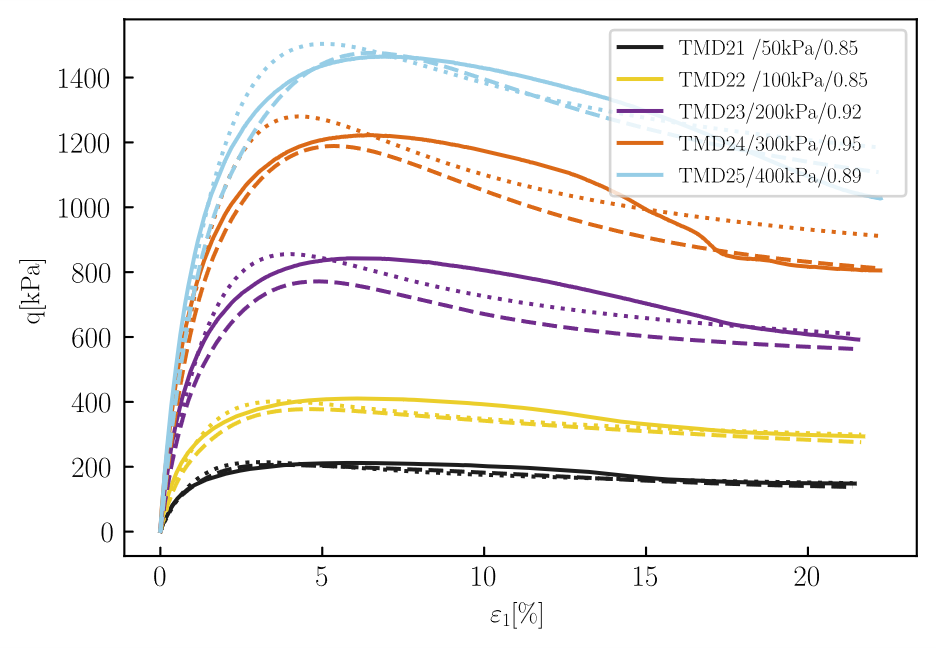


**(d)** **(e)**

**Fig. S21.** Deviatoric stress responses in drained simulations: (a) loose; (b) loose to medium dense; (c) medium dense; (d) medium dense to dense; (e) dense samples


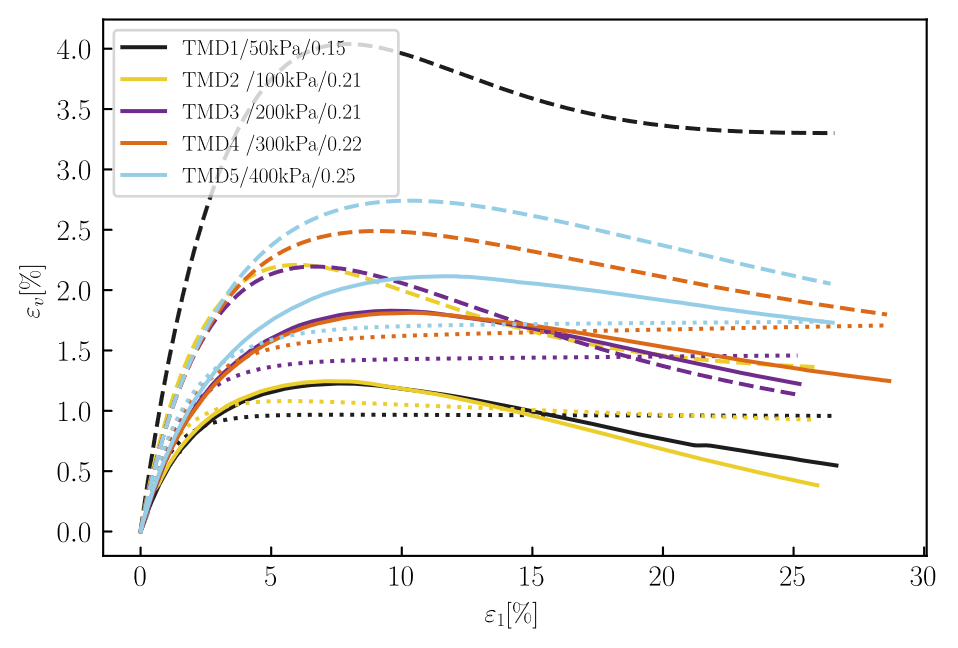

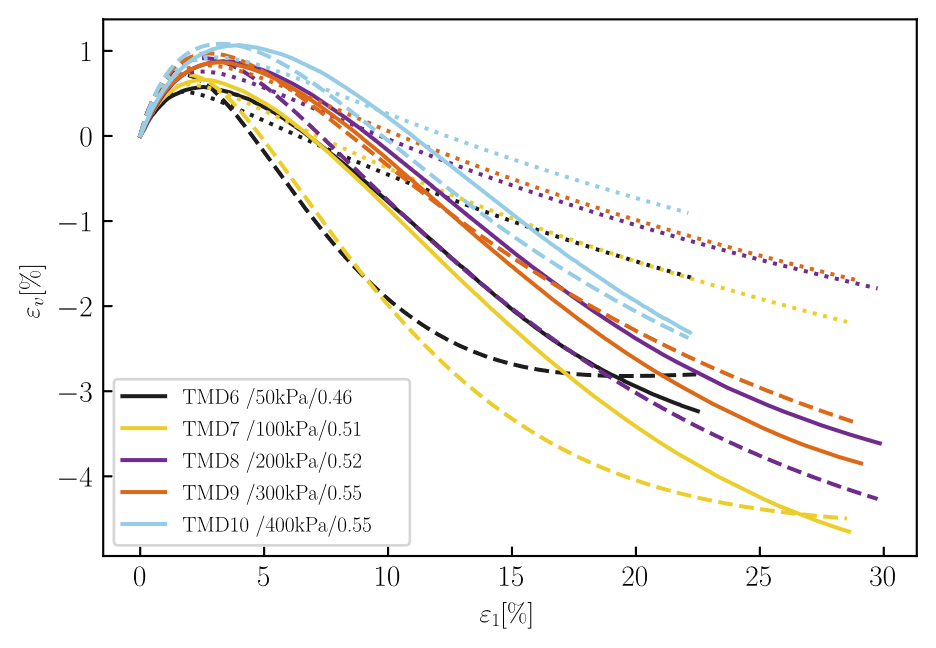

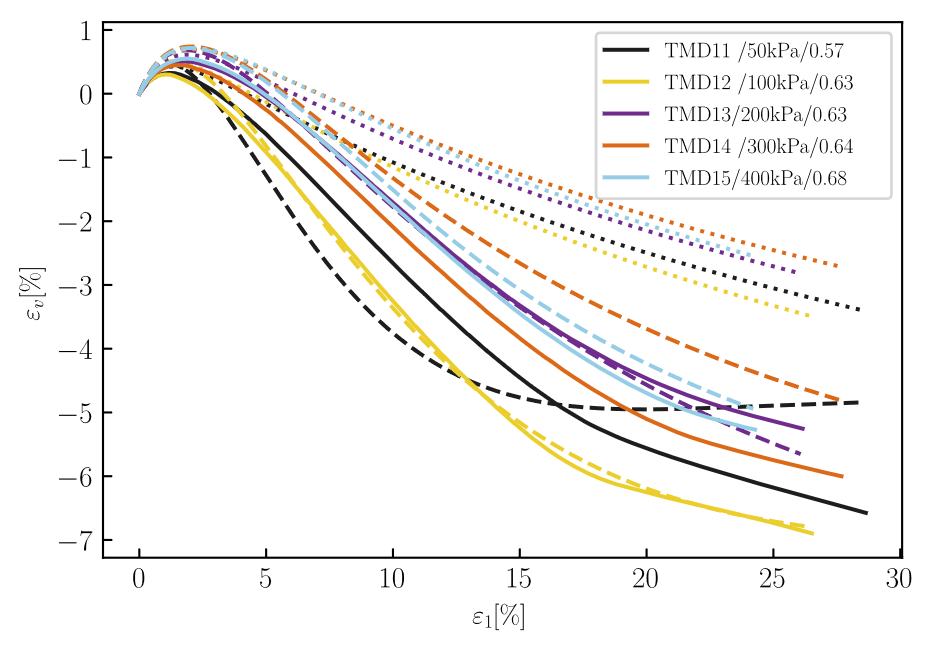


**(a)** **(b)** **(c)**


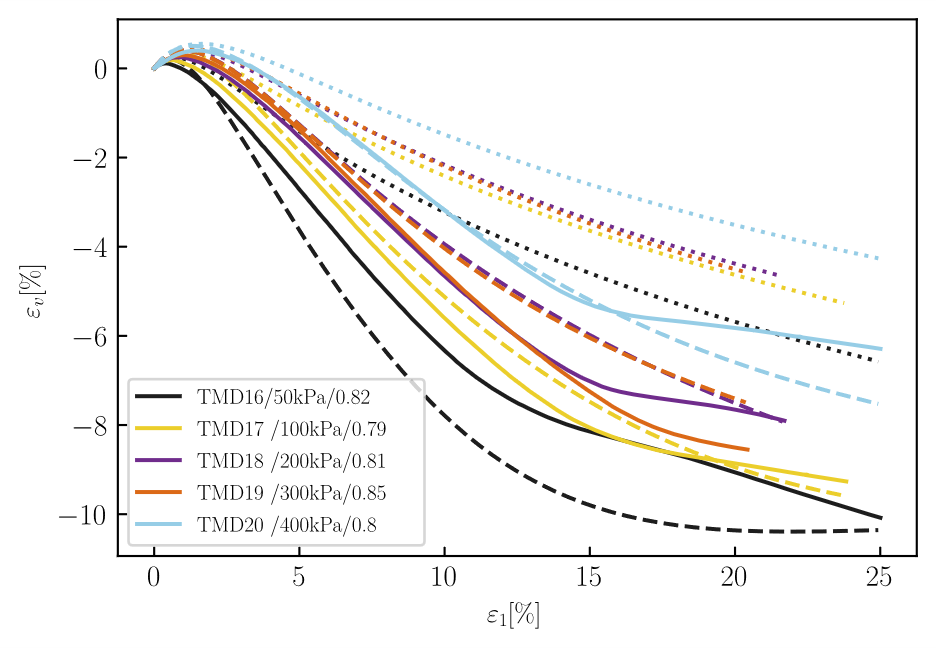

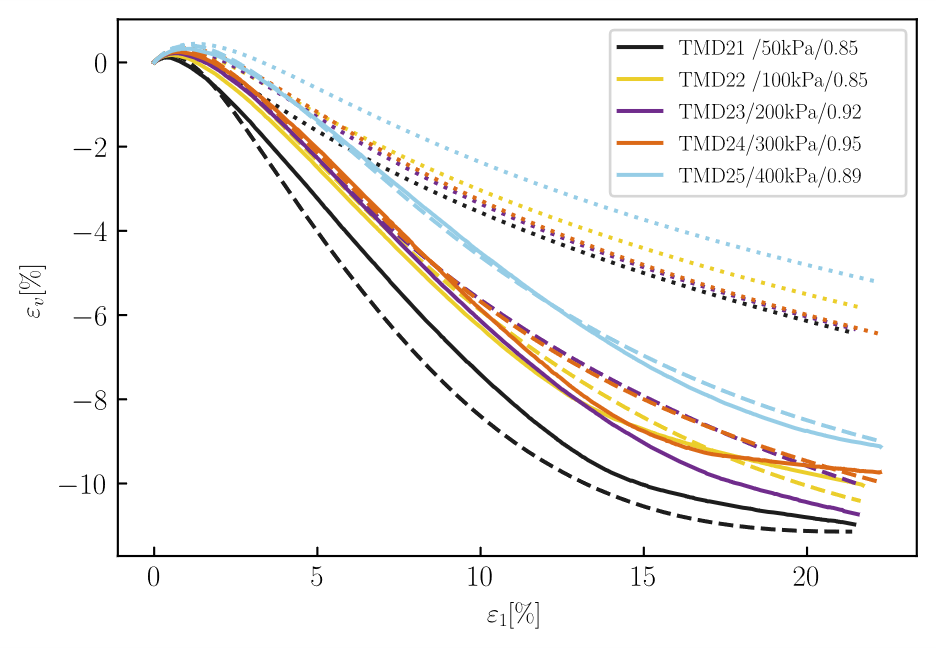


**(d)** **(e)**

**Fig. S22.** Volumetric strain $\varepsilon_{v}$ versus axial strain $\varepsilon_{1}$in drained simulations: (a) loose; (b) loose to medium dense; (c) medium dense; (d) medium dense to dense; (e) dense samples


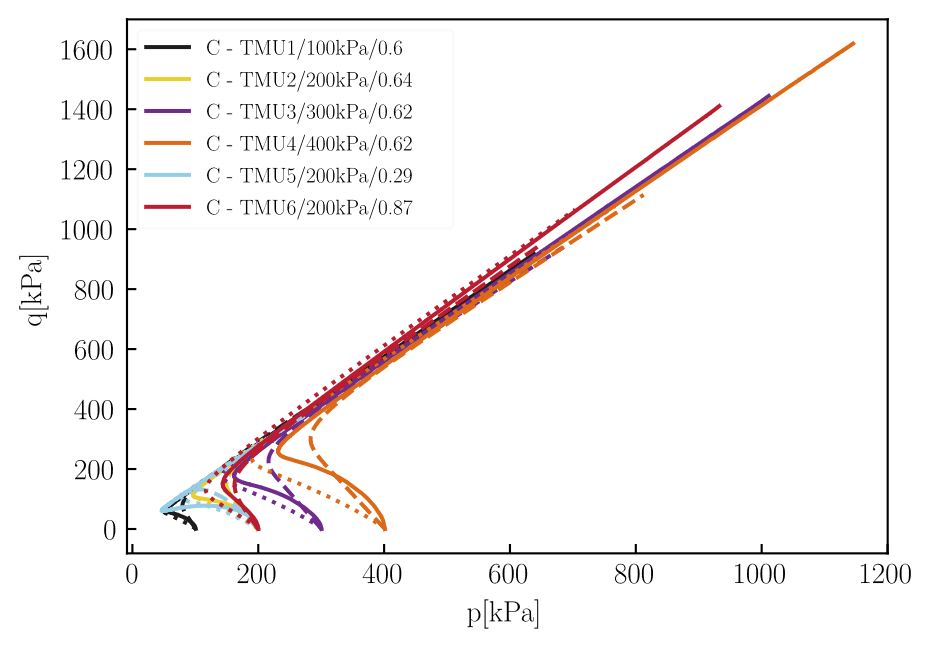

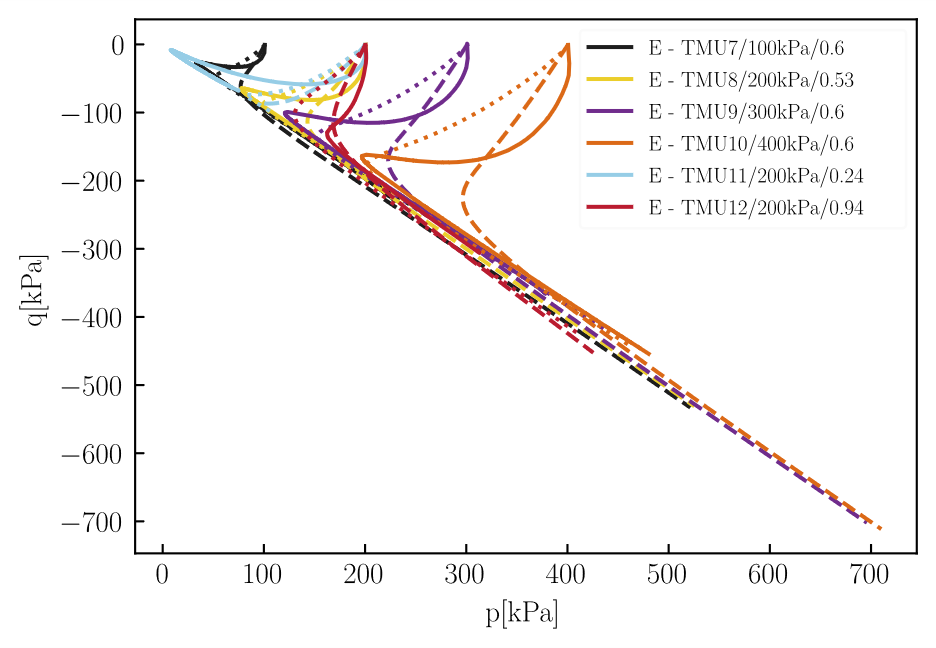


**(a)** **(b)**

**Fig. S23.** Effective stress paths in undrained simulations: (a) compression; (b) extension


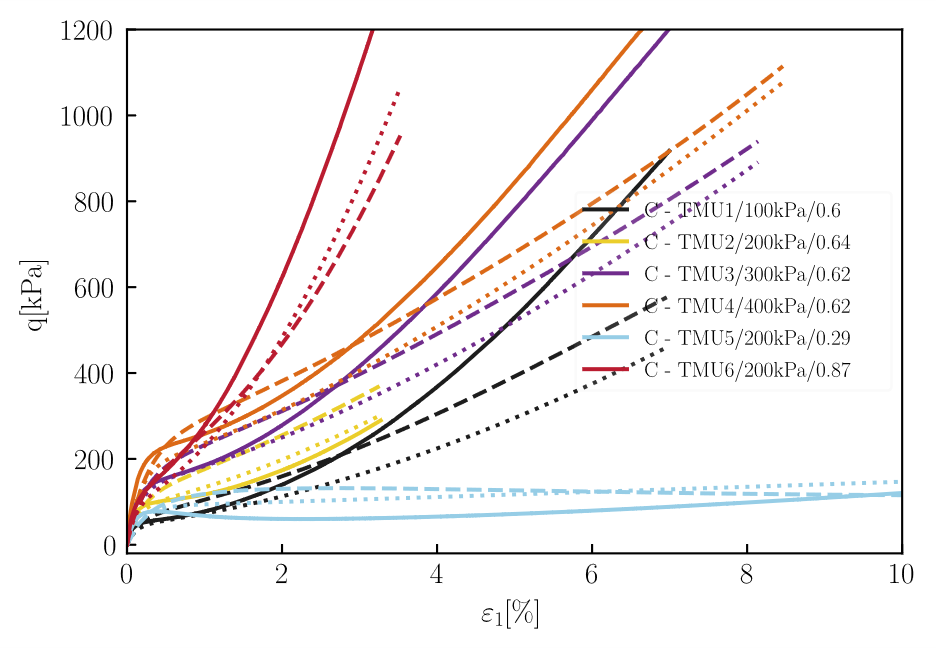

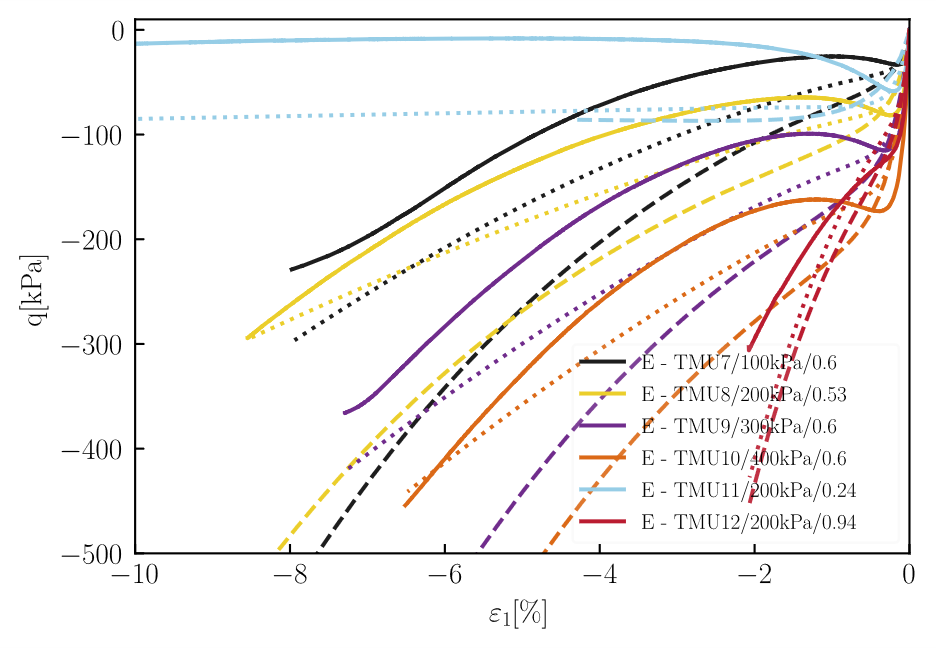


**(a)** **(b)**

**Fig. S24.** Stress-strain relationships in undrained simulations: (a) compression; (b) extension

### Results of Section 4.2.2


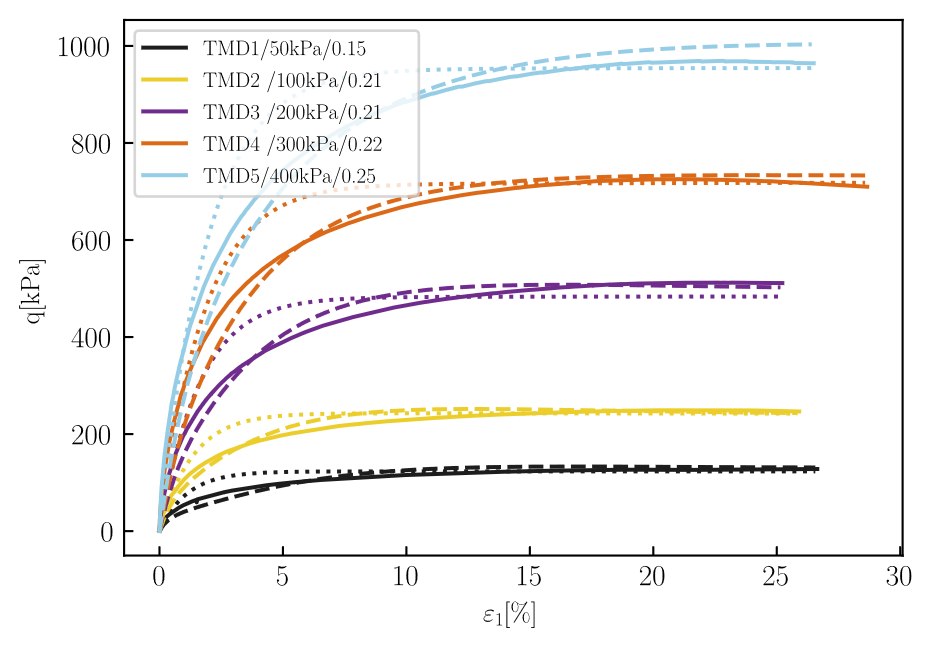

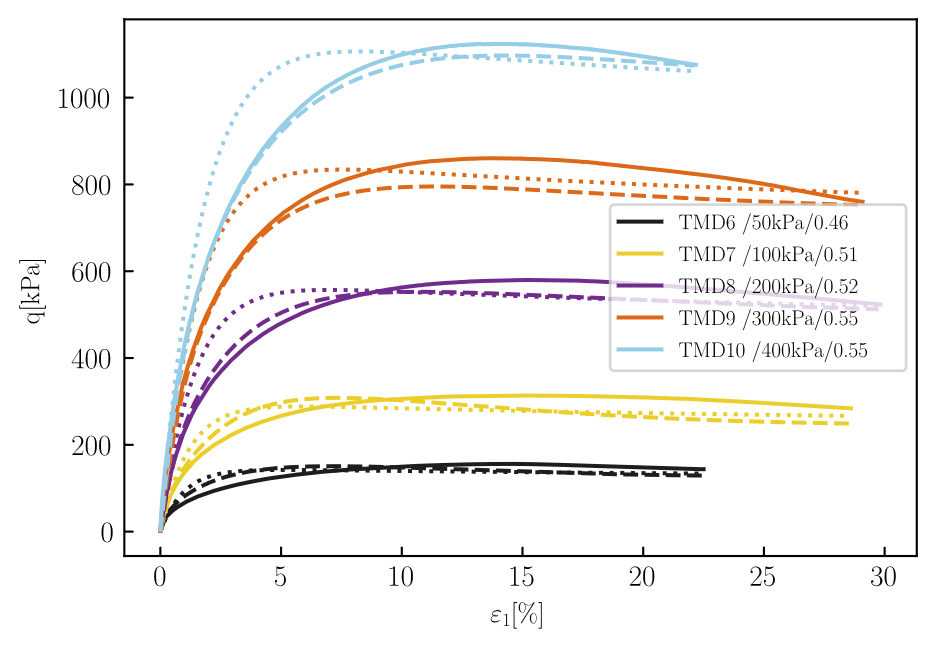

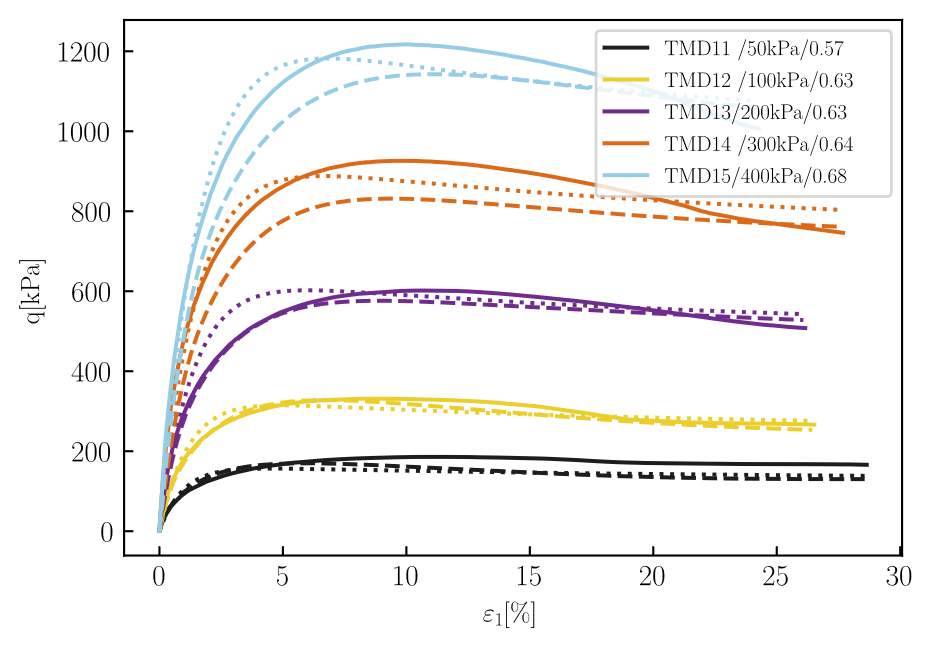


**(a)** **(b)** **(c)**


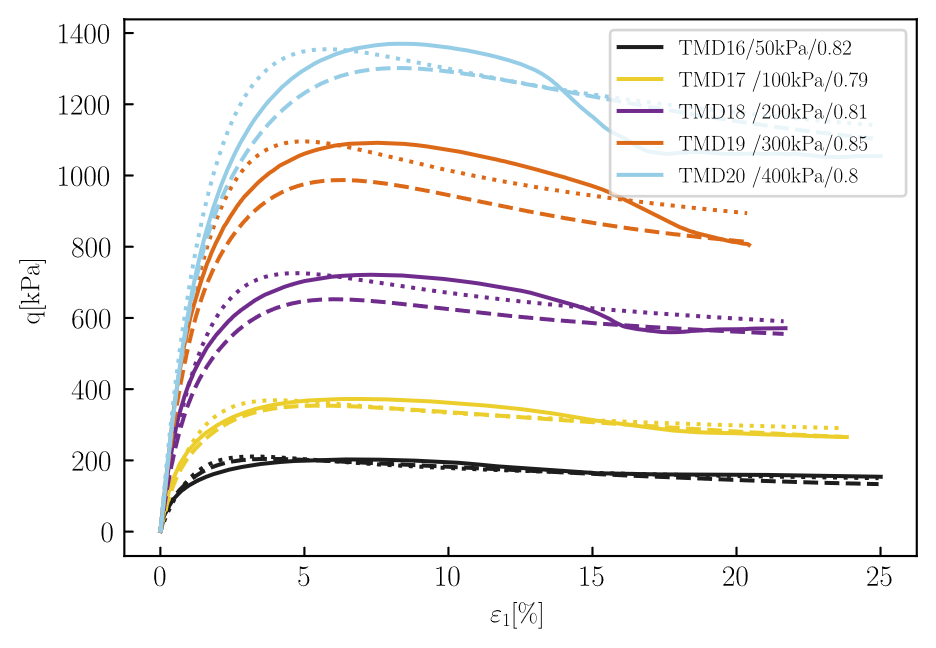

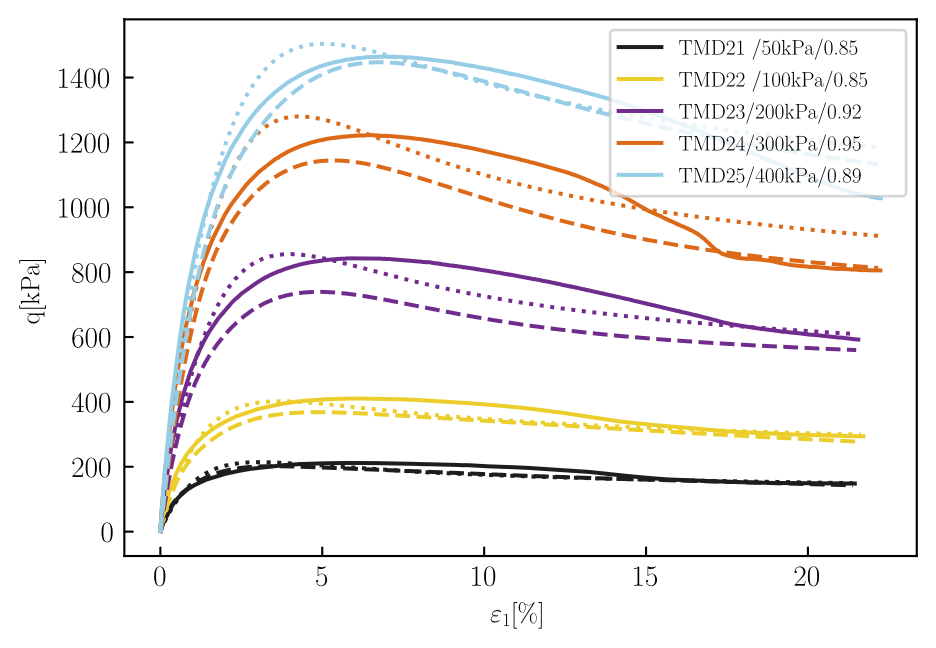


**(d)** **(e)**

**Fig. S25.** Deviatoric stress responses in drained simulations: (a) loose; (b) loose to medium dense; (c) medium dense; (d) medium dense to dense; (e) dense samples


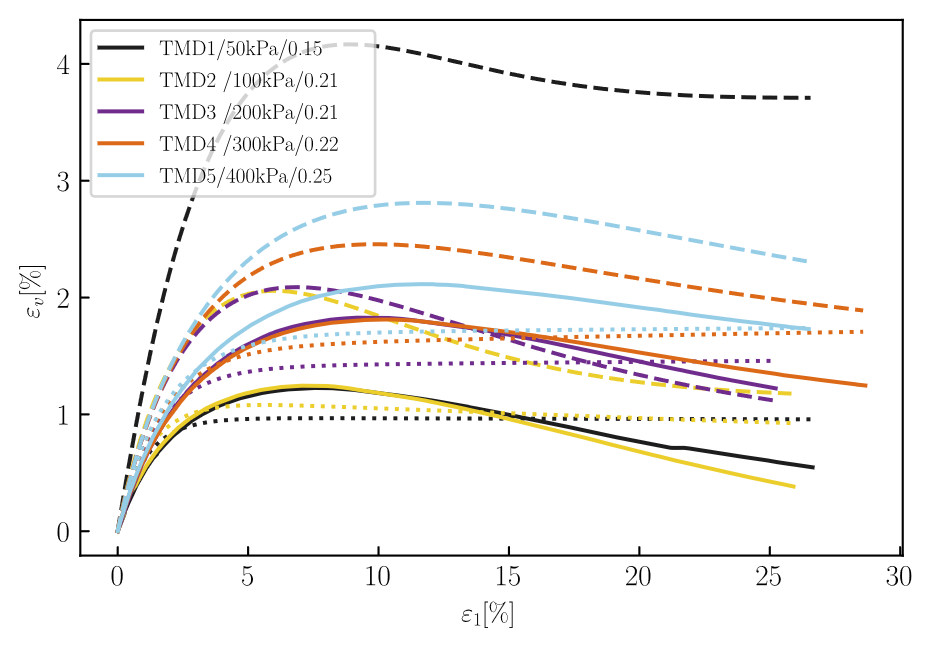

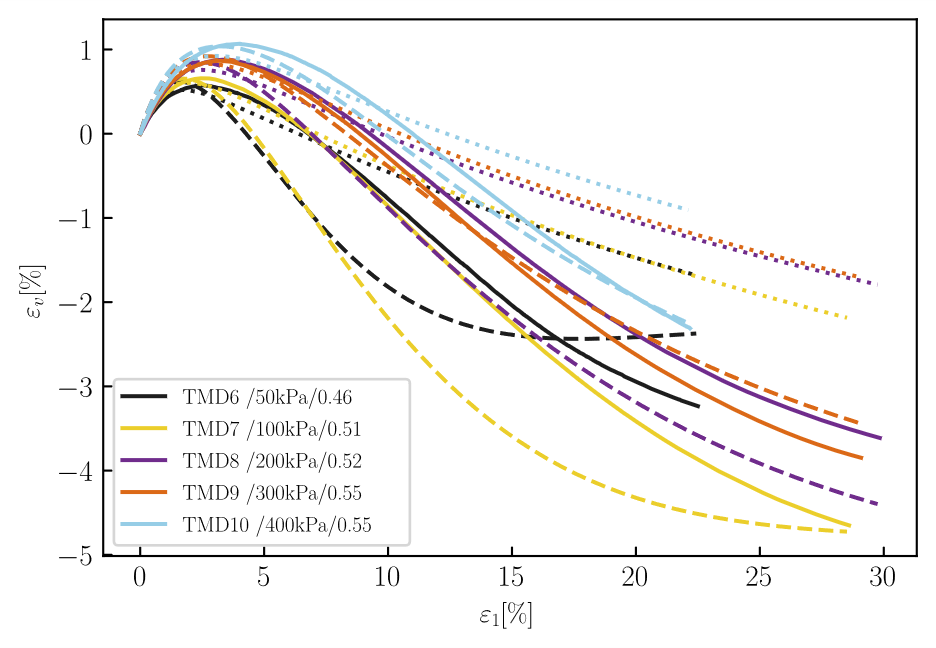

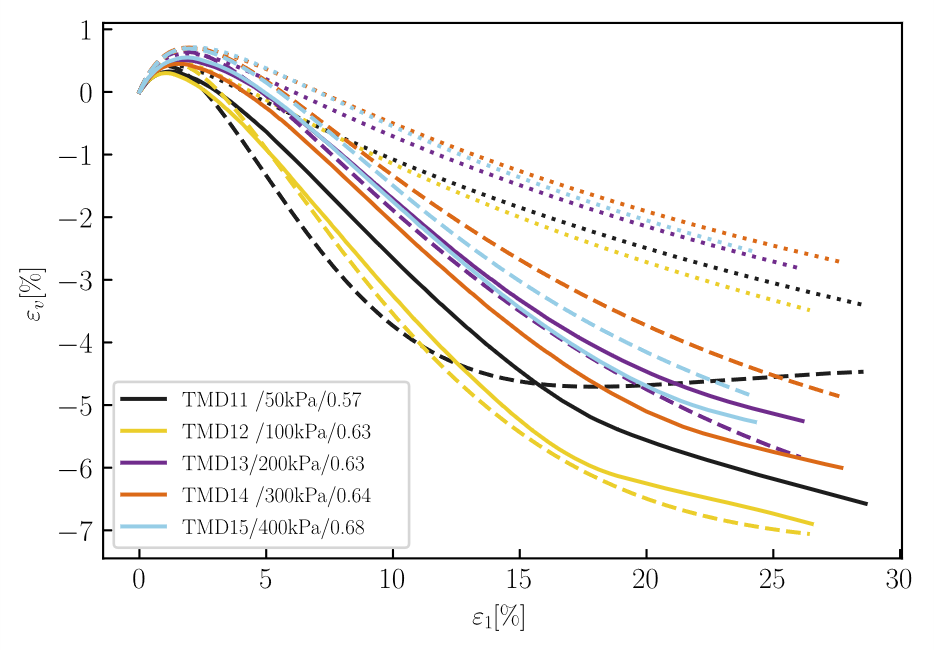


**(a)** **(b)** **(c)**


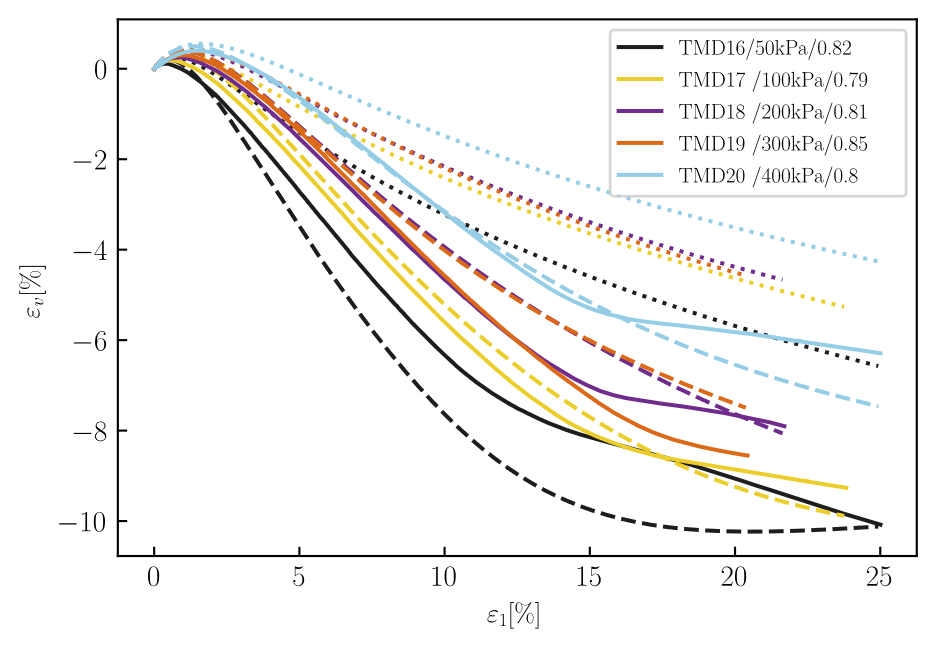

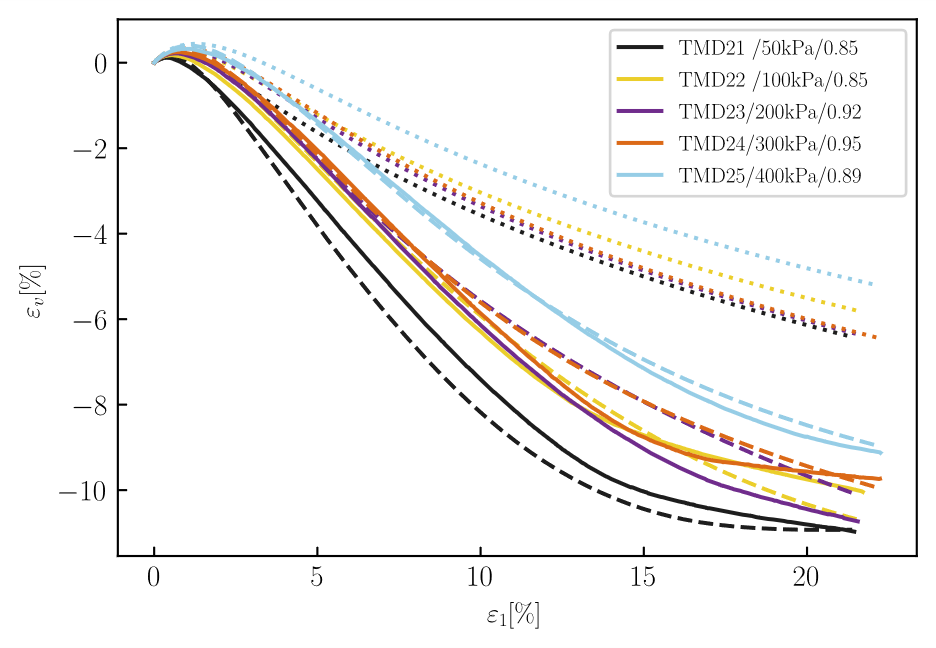


**(d)** **(e)**

**Fig. S26.** Volumetric strain $\varepsilon_{v}$ versus axial strain $\varepsilon_{1}$in drained simulations: (a) loose; (b) loose to medium dense; (c) medium dense; (d) medium dense to dense; (e) dense samples


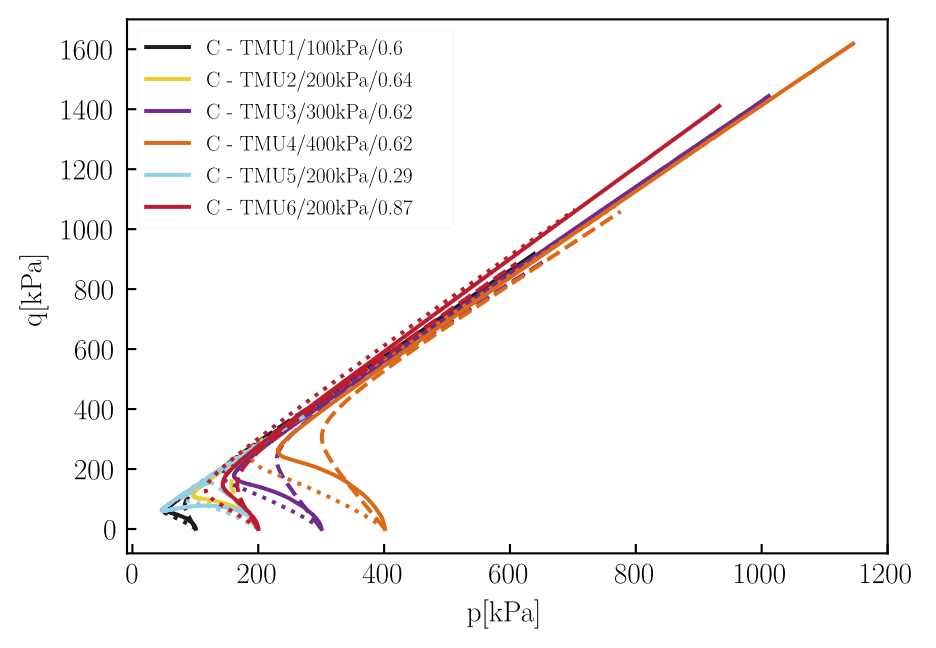

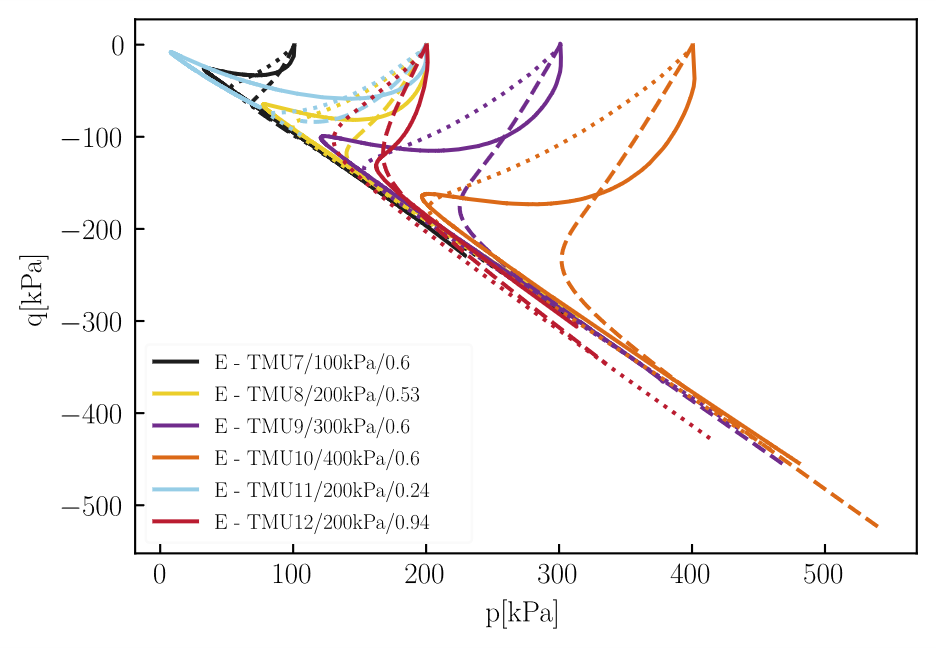


**(a)** **(b)**

**Fig. S27.** Effective stress paths in undrained simulations: (a) compression; (b) extension


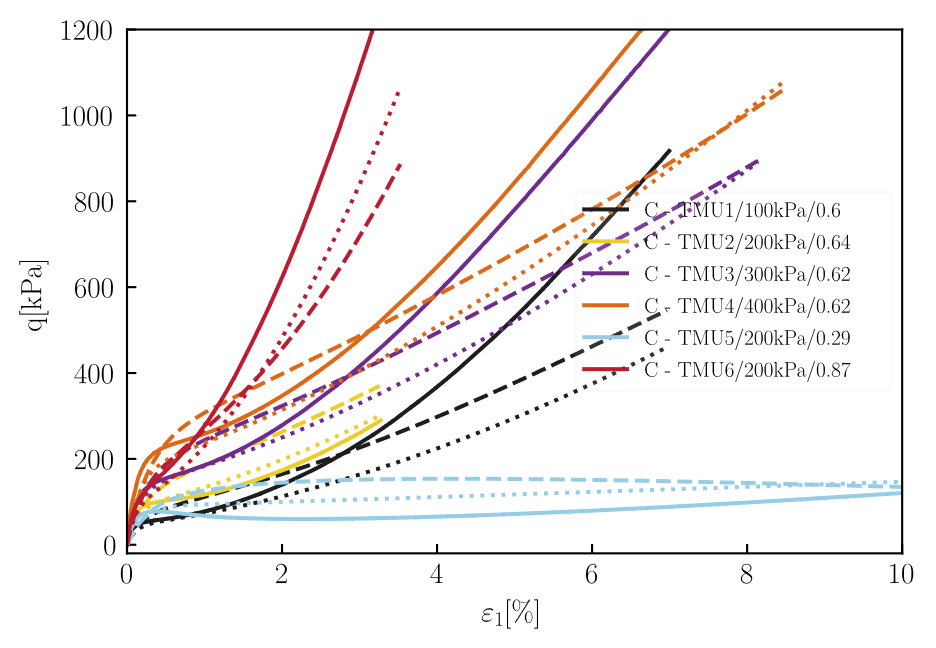

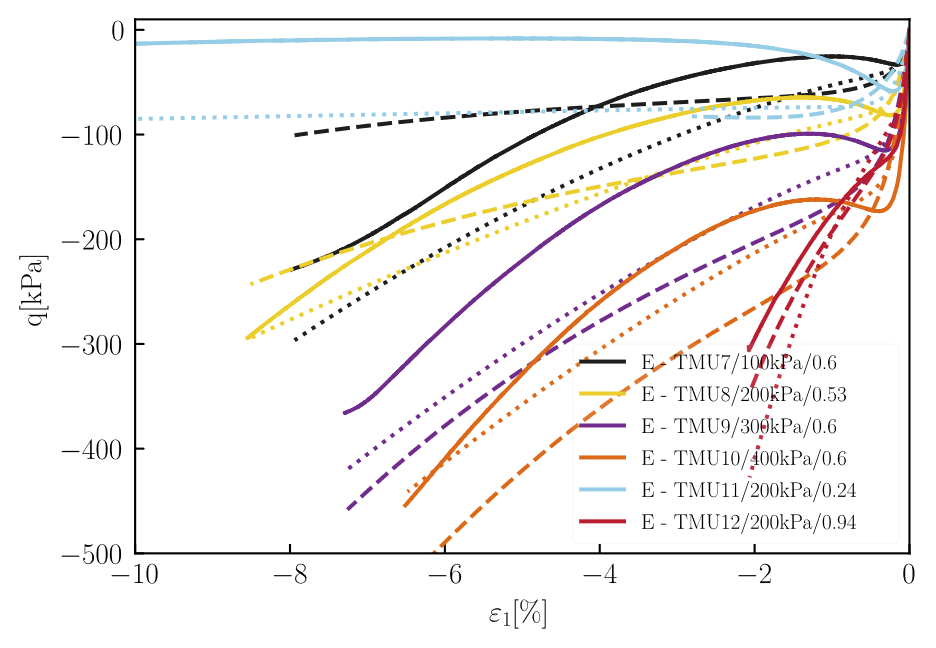


**(a)** **(b)**

**Fig. S28.** Stress-strain relationships in undrained simulations: (a) compression; (b) extension

### Results of Section 4.3.1


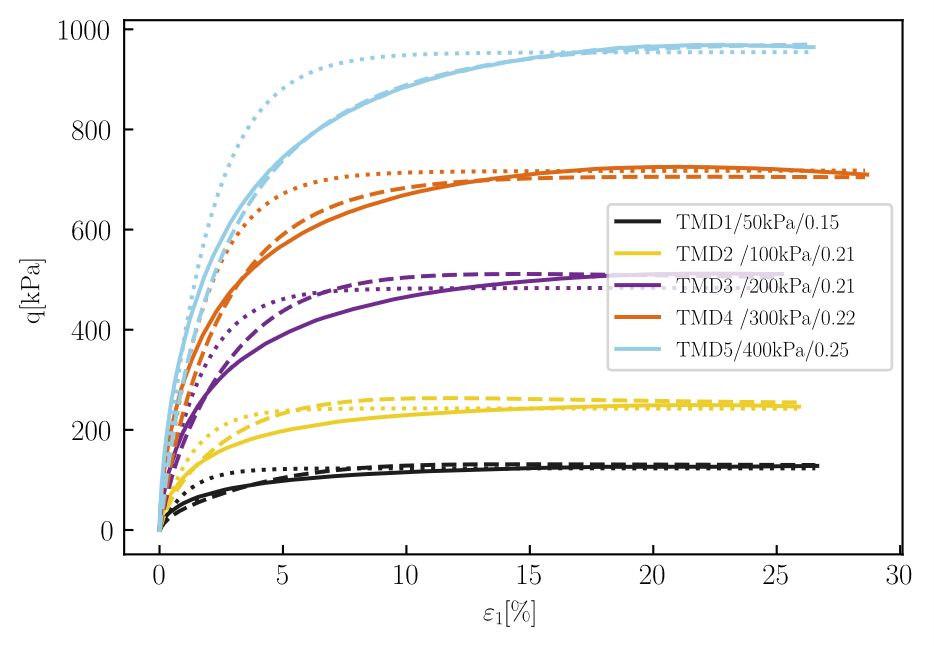

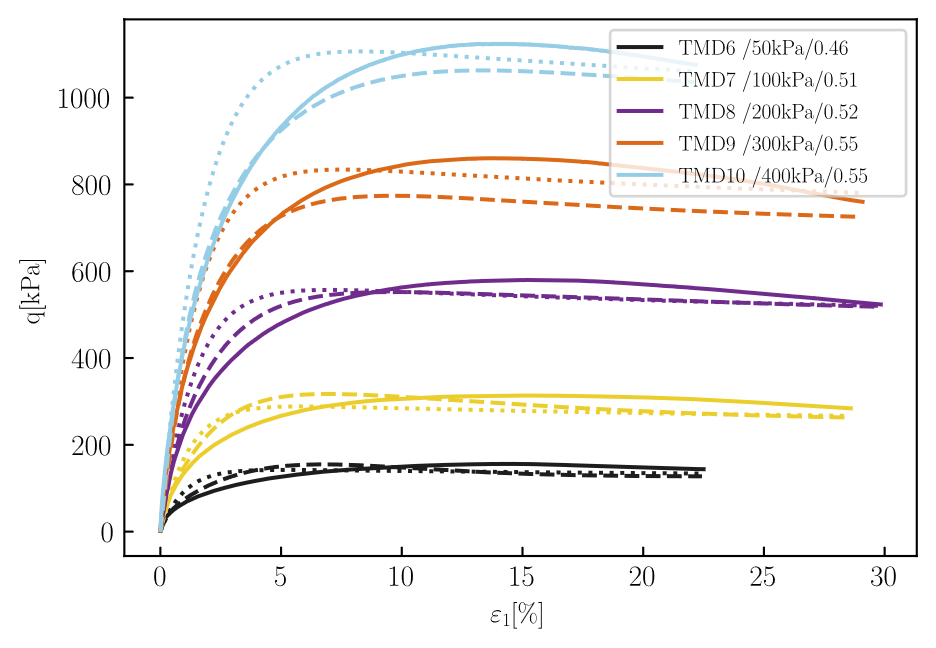


**(a)** **(b)** **(c)**

**(d)** **(e)**

**Fig. S29.** Deviatoric stress responses in drained simulations: (a) loose; (b) loose to medium dense; (c) medium dense; (d) medium dense to dense; (e) dense samples

**(a)** **(b)** **(c)**

**(d)** **(e)**

**Fig. S30.** Volumetric strain $\varepsilon_{v}$ versus axial strain $\varepsilon_{1}$in drained simulations: (a) loose; (b) loose to medium dense; (c) medium dense; (d) medium dense to dense; (e) dense samples

**(a)** **(b)**

**Fig. S31.** Effective stress paths in undrained simulations: (a) compression; (b) extension

**(a)** **(b)**

**Fig. S32**. Stress-strain relationships in undrained simulations: (a) compression; (b) extension

### Results of Section 4.3.2

**(a)** **(b)** **(c)**

**(d)** **(e)**

**Fig. S33**. Deviatoric stress responses in drained simulations: (a) loose; (b) loose to medium dense; (c) medium dense; (d) medium dense to dense; (e) dense samples

**(a)** **(b)** **(c)**

**(d)** **(e)**

**Fig. S34.** Volumetric strain $\varepsilon_{v}$ versus axial strain $\varepsilon_{1}$in drained simulations: (a) loose; (b) loose to medium dense; (c) medium dense; (d) medium dense to dense; (e) dense samples

**(a)** **(b)**

**Fig. S35.** Effective stress paths in undrained simulations: (a) compression; (b) extension

**(a)** **(b)**

**Fig. S36.** Stress-strain relationships in undrained simulations: (a) compression; (b) extension

### Adding new layers in fine-tuning model

In this section, we added a new hidden layer to the original structure of the pre-trained model and then performed fine-tuning. Except for the parameters connected to the newly added hidden layer, all other parameters were frozen. The fine-tuning settings are the same as those described in Section 4.1. The results of the simulations performed with the fine-tuned model are presented in this section.

#### Adding one layer before output layer

A new hidden layer consisting of nine nodes was added between the third hidden layer and the output layer. The activation function used was still the bent identity function. Due to the introduction of the new layer, the initial error increased, and the training quickly converged to a local minimum. The final simulation results of the model were mediocre, as shown in Figures A1 to A5.

**Fig. S37.** Loss evolution

**(a)** **(b)** **(c)**

**(d)** **(e)**

**Fig. S38.** Deviatoric stress responses in drained simulations: (a) loose; (b) loose to medium dense; (c) medium dense; (d) medium dense to dense; (e) dense samples

**(a)** **(b)** **(c)**

**(d)** **(e)**

**Fig. S39.** Volumetric strain $\varepsilon_{v}$ versus axial strain $\varepsilon_{1}$in drained simulations: (a) loose; (b) loose to medium dense; (c) medium dense; (d) medium dense to dense; (e) dense samples

**(a)** **(b)**

**Fig. S40.** Effective stress paths in undrained simulations: (a) compression; (b) extension

**(a)** **(b)**

**Fig. S41.** Stress-strain relationships in undrained simulations: (a) compression; (b) extension

#### Adding layers after original output layer with linear activation

After the output of the pre-trained model, a new hidden layer with nine nodes using linear activation was added. This configuration allows the new output of the PeNN to be a linear function of the original model’s output, making it possible to degenerate to the output of the pre-trained model. The training process and simulation results are depicted in Figures A6 to A10. Despite modifications in batch size and learning rate, the performance of the model remained poor and is not displayed further.

**Fig. S42.** Loss evolution

**(a)** **(b)** **(c)**

**(d)** **(e)**

**Fig. S43** Deviatoric stress responses in drained simulations: (a) loose; (b) loose to medium dense; (c) medium dense; (d) medium dense to dense; (e) dense samples

**(a)** **(b)** **(c)**

**(d)** **(e)**

**Fig. S44.** Volumetric strain $\varepsilon_{v}$ versus axial strain $\varepsilon_{1}$in drained simulations: (a) loose; (b) loose to medium dense; (c) medium dense; (d) medium dense to dense; (e) dense samples

**(a)** **(b)**

**Fig. S45.** Effective stress paths in undrained simulations: (a) compression; (b) extension

**(a)** **(b)**

**Fig. S46.** Stress-strain relationships in undrained simulations: (a) compression; (b) extension

#### Adding layers after original output layer with nonlinear activation

In this section, we explore the performance when the new output of the PeNN is a nonlinear function of the original model. The setup remains the same as in the previous section, except the newly added hidden layer employs a Tanh activation function. The training process and simulation results are illustrated in Figures A11 to A15. Similar to previous modifications, adjustments in batch size and learning rate were made, but the model’s performance was still very poor and is not displayed further.

**Fig. S47.** Loss evolution

**(a)** **(b)** **(c)**

**(d)** **(e)**

**Fig. S48.** Deviatoric stress responses in drained simulations: (a) loose; (b) loose to medium dense; (c) medium dense; (d) medium dense to dense; (e) dense samples

**(a)** **(b)** **(c)**

**(d)** **(e)**

**Fig. S49.** Volumetric strain $\varepsilon_{v}$ versus axial strain $\varepsilon_{1}$in drained simulations: (a) loose; (b) loose to medium dense; (c) medium dense; (d) medium dense to dense; (e) dense samples

**(a)** **(b)**

**Fig. S50.** Effective stress paths in undrained simulations: (a) compression; (b) extension

**(a)** **(b)**

**Fig. S51.** Stress-strain relationships in undrained simulations: (a) compression; (b) extension
